# Husbandry-Derived Loading and Multi-Criteria Lightweight Design of an Industrialised Three-Tier Breeding Rack for *Whitmania pigra* and *Hirudo nipponia*

**DOI:** 10.3390/ani16213479

**Published:** 2026-11-03

**Authors:** Xin Du

**Affiliations:** School of Mechanical Engineering, Jiangsu Ocean University, Lianyungang 222005, China; dx2017on@163.com

**Keywords:** industrialised leech farming, multi-tier breeding rack, lightweight design, husbandry-derived load case, combined entropy–AHP weighting, TOPSIS normalisation sensitivity, column buckling, corrosion allowance

## Abstract

Rearing *Whitmania pigra* and *Hirudo nipponia* relieves the hirudin feedstock shortage, and the rack sets its cost and the animals’ environment; earlier designs were judgement-based. Six husbandry requirements become load and geometric inputs; limits (*SF_y_* ≥ 2, *SF_b_* ≥ 2, and *δ* ≤ *L*/200) screen 36 tube and rank 17 bar schemes. The machine is three tiers of one pan, sized by the credible accident (pans full to their rims, 1417 L each, 47.1 kN), not by the 20–60 mm operating depth, where nothing approaches a limit. The three-ring frame is 74.9% of the baseline, so tubes and bars can save at most 25.1%; the tube number governs the mass (61.26%) and stress (74.32%), and strength governs 34 of 36 schemes. Three 30 × 30 × 2.5 mm tubes with eight 5 mm bars weigh 101.34 kg, 18.4% below baseline, at *SF_y_* = 2.09 and *SF_b_* = 3.35 on the 235 MPa minimum for Q235; the lightest section breaks at 315 mm and this one at 481 mm. The 1.1% lighter 25 × 25 mm section clears the criterion only where the capacity and scaling both favour it; the ±8.3% band on the connection factor exceeds the 4.6% margin, its lower end falling below 2.0; one measurement closes it.

## Simple Summary

*Whitmania pigra* and *Hirudo nipponia*, the two species reared for Chinese medicine, are held indoors to supply hirudin, a blood-clotting inhibitor no longer taken from the wild in quantity. The animals are kept in shallow water in circular pans stacked on tall steel racks, and the racks fix the water depth, temperature and working space they experience. They also consume most of the building’s steel, so a lighter rack cuts the cost of the medicine. Earlier designs were chosen by judgement and ignored two things: leeches need damp bedding above the waterline to lay their cocoons, and the steel stands permanently in a warm, humid room. We modelled one such rack for both species in Chinese indoor production, and ranked many tube sizes and numbers, letting the measurements decide each criterion’s weight. The best designs are lighter, and the level a pan reaches when full, not the depth the animals are kept at, sets how much steel can be removed. The outcome is a chart of the steel a safe rack needs at each level. No animal was used or measured here: the requirements come from published husbandry work, and the bedding allowance most needs an on-site measurement.

## 1. Introduction

### 1.1. Medicinal Leeches, Feeding Habits and the Demand for Artificial Rearing

Leeches such as *Whitmania pigra* and *Hirudo nipponia* are traditional Chinese medicinal animals whose salivary hirudin is the most potent natural thrombin inhibitor known. Hirudin binds thrombin specifically, blocking the conversion of fibrinogen to fibrin and thereby preventing thrombus formation, and it additionally exerts anti-inflammatory, anti-fibrotic and endothelial-protective effects [1,2]. The pharmacological value of the group rests on a well-characterised set of salivary peptides. Chromosome-level sequencing of the non-haematophagous leech *Whitmania pigra* has resolved the antithrombotic gene repertoire of the very species used in the present work [3], and Kunitz-type peptides such as WPK5 from the same species inhibit coagulation factor XIa with high specificity, showing that antithrombotic activity is not confined to hirudin itself [4]. Rising incidence of cardiovascular disease and renewed interest in natural therapeutics have increased demand for leech-derived Chinese patent medicines and health products, and leeches remain an officially recognised medicinal material in the current *Chinese Pharmacopoeia* [5], while wild populations have been depleted through overexploitation, making artificial farming indispensable for feedstock supply [1].

### 1.2. Industrialised Rearing Systems and the Problems Observed in Practice

Industrialised multi-tier rearing is itself one of the responses to these constraints. Conventional pond farming suffers from a one-crop-per-year cycle, exposure to extreme weather, and inefficient use of space because of its single-layer layout. Industrialised multi-tier farming shortens the cycle to two or more crops per year and substantially raises output per unit water volume [2]. The same transition is visible across aquaculture as a whole: recirculating systems decouple production from ambient conditions and make the energy demand of a facility quantifiable and, therefore, optimisable [6], while an efficiency analysis of Chinese deep-sea cage aquaculture shows that output growth now depends more on the engineering quality of the facilities than on the cultivated area [7]. In this context, the breeding rack becomes the principal load-bearing member: its steel consumption directly determines the construction cost, and its structural reliability governs long-term serviceability. Lightweight design of the rack, therefore, has direct engineering and economic value.

### 1.3. The Husbandry Duty of the Breeding Rack

Leech farming is, however, an animal-production activity before it is a structural one, and the facility fixes the physical environment in which the animals are held. Reviews of intensive and recirculating production treat the production method, not the species alone, as a determinant of welfare outcome, and identify environmental enrichment as decisive in such systems [8,9]; raising the number of tiers raises the effective stocking density, which measurably alters growth and the physiological stress response [10,11], while critical appraisals of recirculating facilities conclude that process logic and engineering, rather than biology, are frequently the limiting factor [12]. Health management adds a further constraint, since farmed medicinal leeches are susceptible to bacterial infections originating in their holding environment [13]. The rack is, therefore, not a neutral background structure: its stiffness and stability decide whether tier geometry, water depth and flow remain uniform across the farm, and, hence, whether every animal meets the same husbandry conditions; and because it is the largest single steel consumer of the facility, a lighter rack that preserves that uniformity is a husbandry advance and not merely a cost saving.

Leeches are carnivorous, and the two species relevant to Chinese production are fed differently. *Hirudo nipponia*, the blood-feeding species of the pair, is haematophagous and is fed on blood through a membrane or on a host, as *H. medicinalis* is in European production (Section 4.4), whereas *Whitmania pigra*, the non-haematophagous species whose genome has been sequenced [3], feeds on aquatic gastropods and small invertebrates. The distinction fixes both the load case and the cleaning duty: blood feeding leaves a residue that must be flushed promptly and imposes a high organic load, so a blood-fed unit needs frequent water exchange and unimpeded access to every surface, while a snail-fed unit carries the shell mass of its prey and produces a mineral detritus that settles in the pan. Each regime adds a live load varying with the feeding cycle, and each requires that the water in every tier be replaceable at the same rate, because the oxygen demand of the residue is proportional to its mass. Neither was represented in the earlier design, in which water was treated purely as added mass and feeding was not mentioned at all.

Cocoon deposition is the husbandry step the earlier design omitted most conspicuously. Gravid adults leave the water and deposit cocoons in moist, well-aerated substrate above the waterline—typically damp peat, coir or soil in a tray or a raised section of the pan; in the wild, the same function is served by waterfowl nests, which leeches use for reproduction and as a resting site [14], the animals attaching to porous media rather than smooth surfaces [15]. The docking station must, therefore, provide a wetted but unsaturated substrate layer, a water level deliberately kept below it, and a porous surface that does not dry out between waterings. The last follows from the geometry: the water must stay shallow, of the order of a few centimetres, if the substrate is to sit above it inside the same vessel. The operating depth is, consequently, not a free process variable—it is fixed by reproductive behaviour, and it fixes the mass the rack carries. That coupling is why depth is carried through the analysis as a parameter rather than assumed.

The stocking density must likewise be bounded rather than maximised. Adding tiers raises the number of animals per unit floor area and alters growth and the stress response [10,11], so a density ceiling must be fixed before the rack is dimensioned, because it determines the tray area, the water volume and, hence, the load. The structural objective is, therefore, reformulated: not to maximise animals per square metre, but to raise the load-bearing efficiency—the same husbandry duty with less steel—subject to that ceiling and to uniform conditions across tiers.

Nine husbandry requirements govern the design, and they are stated here in full before being converted into structural terms. They are (1) water renewal and unobstructed access to every pan; (2) maintenance of water quality, which fixes the volume a pan must hold at its operating depth and the daily exchange of it; (3) a rearing room held at 22–28 °C and above 80% relative humidity; (4) dissolved oxygen above the species requirement, which fixes the wetted surface area per tier; (5) a wetted but unsaturated cocooning substrate above the water line; (6) conditions that are uniform across tiers; (7) the feeding regime of the species; (8) a stocking-density ceiling; and (9) the handling loads imposed by harvesting and grading. Requirements (1)–(6) are converted into the structural inputs listed in Section 2.2, five of them into numerical values and the sixth, (6), into a geometric constraint; requirements (7)–(9) are named in Section 4.5(4) among the quantities this study does not quantify.

### 1.4. Lightweight Design of Load-Bearing Structures in Aquaculture

Several studies have addressed the mechanical analysis and optimisation of aquaculture load-bearing structures. Tian et al. [16] developed a parametric finite element framework for a 40 m diameter deep-sea floating-raft cage, applying wind, wave and current loads through secondary development of ANSYS 2020 R1 and then optimising the structure with a genetic algorithm; the cage mass was reduced by 17.98% while maintaining structural strength. Zhang et al. [17] proposed a novel deep-sea cage combining a spatial grid structure with H-shaped steel members, validated it by numerical simulation and field measurement in the South China Sea, and reported a uniform stress distribution, low steel consumption and a reserve strength ratio of 3.28. Wang et al. [18] studied a rotatable horizontal aquaculture cage using single-factor experiments and response surface methodology to relate cage radius, cone angle and length to displacement and volume, forming three optimisation schemes that reduced displacement by 10.07% and increased volume by 44.70%. Attention has also begun to move from the net to the supporting structure: Li et al. [19] carried out a conceptual design and structural performance analysis of an innovative deep-sea aquaculture platform, and Shen et al. [20] quantified the hydrodynamic response of a fixed aquaculture platform carrying a horizontal cylindrical cage under combined waves and currents, showing that the platform rather than the net can govern the design. Collectively, these studies show that parametric modelling combined with surrogate models or intelligent algorithms has become the mainstream approach for optimising aquaculture structures and that the load-bearing frame is the component in which the mass can most realistically be removed.

Methodologically, structural lightweight design is inherently a multi-objective decision problem requiring a trade-off among mass, stiffness, strength, cost and manufacturability. Jiang et al. [21] combined NSGA-II with entropy-weighted TOPSIS (technique for order preference by similarity to an ideal solution) for the lightweight design of a dump truck carriage, first generating Pareto solutions from a Kriging surrogate model and NSGA-II and then selecting the optimum by entropy-weighted TOPSIS, achieving a 3.7% mass reduction. Chu et al. [22] integrated intuitionistic fuzzy number theory, the entropy method and TOPSIS to quantify subjective judgements and uncertainty when selecting lightweight schemes for automotive safety structures. In the machine-tool field, Chen et al. [23] established a TOPSIS model with alternative distance measures for structural priority design, and Li et al. [24] obtained candidate bed structures through sensitivity analysis and multi-objective optimisation before selecting the optimum by TOPSIS. The same recipe has since spread through mechanical design: surrogate-assisted lightweight optimisation of an electric towing winch couples Kriging, NSGA-III and TOPSIS [25]; multi-objective lightweight optimisation of an aluminium alloy vehicle subframe confronts the mass–stiffness conflict directly [26]; load-distribution optimisation of a steel storage rack by a genetic algorithm shows that member arrangement, rather than section enlargement, drives the load-bearing capacity of rack-like structures [27]; and a comparative evaluation of steel-welded and modular aluminium rack structures demonstrates that load-bearing performance has to be weighed against cost efficiency instead of being optimised in isolation [28]. On the weighting side, combined ANP–entropy–TOPSIS frameworks are used to obtain defensible criteria weights when expert judgement and measured data have to be reconciled [29], and entropy weighting combined with gray-TOPSIS has been applied to machining-process selection, where the criteria carry different units [30]. These works jointly demonstrate that, in a discrete design space, ranking Pareto solutions by an objectively weighted multi-criteria method effectively removes manual intervention [21].

### 1.5. Research Gaps, Objectives and Contributions

Despite this substantial body of work, four gaps remain in the lightweight design of aquaculture load-bearing structures. First, the objectivity of the weighting is insufficient. Many engineering design studies still use empirical weights, and some even add quantities with different physical dimensions—mass in kg, deformation in mm, and stress in MPa—directly by nominal weights. The resulting actual contribution can diverge sharply from the nominal weight, so that the optimum is governed by the magnitudes of the units rather than by engineering intent. Second, the effect mechanism of the design variables is not quantified. Most studies judge visually from line charts which factor matters more, without analysis of variance, and, therefore, cannot detect interactions; conclusions that hold only at certain factor levels are easily generalised incorrectly. Third, the verification scope is incomplete. Aquaculture facilities operate permanently in humid, immersed conditions, and their columns are slender compression members, yet existing studies generally perform only static strength checks and omit the identification of the intermediate-column buckling regime and the effect of corrosion allowance on lightweight conclusions. The omission is consequential in both directions: cold-formed and built-up steel columns of the kind used here can lose capacity through local–global interaction well before the yield stress is reached [31], and coating durability under continuous condensation and immersion determines how much of the nominal section actually survives the service life [32]. A lightweight conclusion drawn from the as-new section can, therefore, be reversed once stability and corrosion are treated as design constraints. At the same time, second-order response surface models fitted and screened by analysis of variance have become standard in mechanical design optimisation [33], yet they are rarely used to expose interactions among the geometric variables of an aquaculture frame.

A fourth and more fundamental gap is that the husbandry requirements of the species are never carried into the structural problem. Rack-design studies—including the author’s earlier unpublished design of the rack examined here—treat the water as a dead load and set aside the biology that decides how much water there is and what else the structure must carry. The load case is, consequently, under-specified: the operating water depth is not reported, the water mass is not computed from the vessel geometry, the moist substrate in which the cocoons are deposited is absent from the model, the additional load imposed by feeding is not considered, and no density ceiling accompanies the stated aim of raising stocking density. A structural optimum obtained from such a load case cannot be transferred to a farm, however rigorous the numerical work behind it. The present paper closes this gap by deriving the load case explicitly from husbandry requirements and by expressing the outcome as a function of the single process variable that the producer actually controls, namely, the operating water depth.

**Figure 1:**
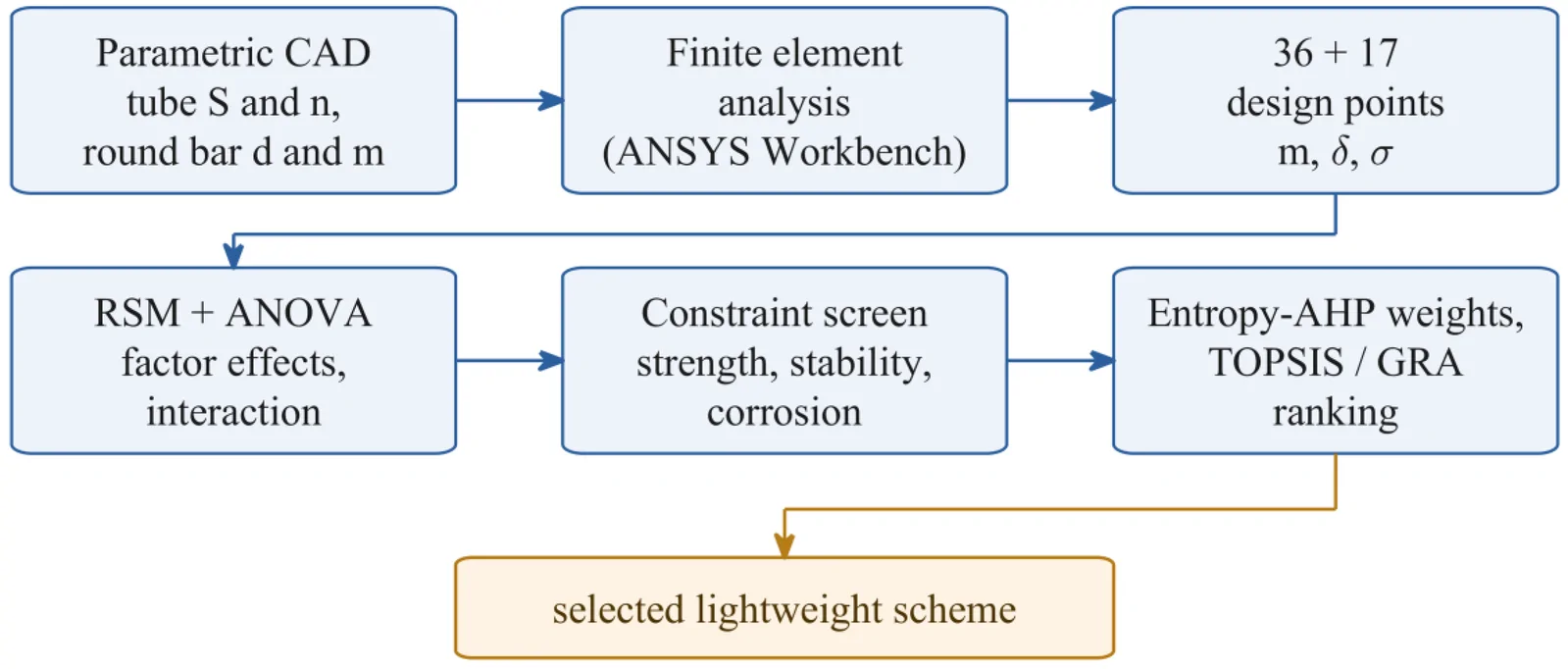
Workflow of the proposed lightweight design procedure.

To address these gaps, this paper takes a three-tier annular industrialised leech breeding rack as the object and establishes a complete lightweight design procedure comprising parametric FEA, response surface and variance analysis, combined-weight multi-criteria decision making, and stability and serviceability checks. The main contributions are as follows:

1. **A husbandry-derived load model.** Six of the nine requirements in Section 1.3—water renewal, water quality, temperature, dissolved oxygen, the cocooning substrate and the uniformity of conditions across tiers—are translated into explicit structural design inputs, as presented in Section 2.2, five as numerical values and the sixth, (6), as a geometric constraint, and the load case is expressed in terms of the operating water depth, the tray geometry and the substrate layer. The remaining three, the feeding regime, the stocking-density ceiling and the handling loads of harvesting and grading, are declared as not quantified (Section 4.5(4)) rather than passed off as inputs. The load case that the original finite element solutions actually represent is reconstructed and stated, rather than left implicit.
2. **A constraint-first decision procedure with a multi-directional sensitivity analysis.** Mandatory strength, stability and deflection constraints are applied before any ranking, so that preference weighting operates only within the feasible set. AHP, entropy and combined weights are reported separately rather than only in combination, and the sensitivity of the outcome is examined with respect to the preference judgement (4913 judgement matrices), the data (leave-one-scheme-out entropy weights), the criterion set, the alternative set and the normalisation convention. The last of these changes the ranking substantially (Spearman *ρ* = 0.172), which identifies the normalisation convention as the dominant methodological choice in this class of problem.
3. **A quantified response mechanism with the analysis-of-variance caveat respected.** Two-factor ANOVA and second-order response surface regression quantify the contributions of the tube edge length and tube number to the mass, deformation and stress; because a single-replicate factorial provides no pure error, the evidence for interaction is substantiated independently by a stratified simple-effect comparison and by the fraction of the residual sum of squares explained by the fitted interaction term.
4. **A corrected, physically interpretable mass decomposition model.** The round-bar contribution is written explicitly instead of being absorbed into the constant term, so that the model reproduces the tabulated mass of the eight-tube and eight-bar configuration, and it is validated against thirteen schemes that took no part in its calibration. The mass that can be reached within this structural form is bounded twice: the frame fixes an arithmetic ceiling of 25.1%, and the lightest standard section that survives every criterion realises 18.4%.
5. **A depth-resolved limit-state design chart that carries its own verification protocol.** Intermediate-column buckling regime identification, a comparison of the strength and stability margins in the design case, and a corrosion allowance carried into the admissible water level replace the qualitative treatment in earlier work. The result is a chart from which the water level at which each standard section is broken can be read directly, so that a design case stated as a level returns the section rather than the reverse. The 99.21 kg extreme is carried as a predicted candidate whose connection effect is bounded by solved substitution factors; the three-dimensional solid-contact protocol that resolves it is specified with its acceptance criteria. The overall technical route is shown in Figure 1.

## 2. Materials and Methods

### 2.1. The Rearing System and Rack Configuration

The object of study is a three-tier annular industrialised leech breeding rack. One tier is one structural unit: three annular rings of radii 255, 625 and 995 mm, 24 radial beams welded to them, and vertical square steel tubes 890 mm long that carry the load of that tier down to the tier below or, in the bottom unit, to the floor. The baseline unit has six 50 × 50 × 2.5 mm tubes; the section and the number of those tubes are the design variables in Section 2.5, and they are the same in all three units. Round steel bars are welded to the tubes and tie the mesh frame into an integral body. The three units are stacked vertically, and each carries one rearing pan, so one machine is three tiers, three pans and three rack units, and the baseline unit mass of 124.14 kg yields 372.42 kg of bare steel for the machine. The load path is, therefore, not shared between tiers: the pans load their own tier, and the tubes of each unit carry the weight of that unit and of everything above it, which is why the column check in Section 2.8(2) is written on the whole-machine load. The structural objective is not to maximise the animals per unit floor area but to raise the load-bearing efficiency—the same husbandry duty with less steel—subject to a stocking-density ceiling specified by the operator (Section 4.5(4)), to equivalent water depth, temperature and renewal across the three tiers, and to access for feeding, cleaning and harvest. In structural terms, the rack mass is minimised subject to the strength, stability and deflection constraints in Section 2.8 at the declared operating depth. The density ceiling enters only as an operator input (Section 4.5(4)); Equation (1), therefore, carries the operating depth, the substrate allowance and the self-weight, and the biomass term is the operator’s to supply.

The rearing pans are moulded in glass-fibre-reinforced plastic (GFRP), one per tier: 67.09 kg each and 201.27 kg for the three. Their as-built geometry is measured (Section 2.3), not inferred from the rendering. The foundation is a concrete floor. The material properties are listed in Table 1.

The yield strength adopted for every strength check in this paper is 235 MPa, the minimum value specified for Q235 at a wall thickness of 2.5 mm, and the ultimate strength is taken as 460 MPa. The earlier design from which this work starts used 250 MPa, 6.4% higher, and that figure is used for no check here: a margin quoted against a typical rather than a certified minimum is a margin the mill certificate does not have to deliver. The 235 MPa is a reference strength for the safety-factor checks of Equation (2) and not a code design strength—GB 50017 [34] yields *f* = 215 MPa for Q235 at this thickness, 8.5% below it, so a check against the design strength would reduce every strength margin reported below by a further 8.5%—and it remains an assumption about the delivered steel rather than a measured property. Adopting the nominal minimum in place of the earlier reference is not a bookkeeping change. It lowers every strength safety factor by 6.0%; it lowers the critical load of every section, by 0.8% for the 20 × 20 mm tube, whose slenderness sits just below the transition, and by 5.7% for the 60 × 60 mm one, because the Johnson parabola of Equation (13) is written in *σ_y_* as well as in *λ*; and in the design case, in Section 2.3, it removes from the admissible set the scheme that the higher reference recommended and moves the recommendation one standard section up (Section 3.9 and Section 3.10(4)). All buckling checks and all safety factors in Section 3.8, Section 3.9 and Section 3.10 are, therefore, on 235 MPa, and no result in this paper carries a second strength basis. For an isotropic linear-elastic material, only the Young’s modulus and the Poisson’s ratio are required; the bulk and shear moduli are derived columns, and the values in Table 1 are consistent with them: *K* = *E*/[3(1 − 2*ν*)] and *G* = *E*/[2(1 + *ν*)]. The glass-fibre-reinforced plastic pans are far stiffer than the steel frame at the load levels involved, and their role in the analysis is to distribute the water load onto the mesh deck.

### 2.2. Husbandry Requirements Translated into Structural Design Inputs

The rack can be dimensioned only once the requirements of the animals have been converted into quantities that a structural model can accept. Of the nine requirements identified in Section 1.3, five convert into a numerical design input and one fixes a geometric constraint, and all six are listed in Table 2 with the value adopted and the basis of that value. The remaining three—the feeding regime, the stocking-density ceiling and the handling loads of harvesting and grading—are named in Section 4.5(4) among the quantities this study does not quantify, and they are deliberately kept out of Table 2 so that nothing in it reads as an input when it is not one.

**Table 2:** Husbandry requirements of the two cultured species, *Whitmania pigra* and *Hirudo nipponia*, that convert into a structural design input, the input derived from each, and the basis of each input.

Husbandry Requirement	Structural Design Input	Adopted Value	Basis
Water renewal and cleaning of every tier	Unobstructed access to each pan; no member crossing the pan rim	Clear opening ≥ 150 mm in front of every tier	Engineering assumption for manual access at feeding and harvest; the handling load itself is not quantified (Section 4.5(4))
Maintenance of water quality (ammonia, nitrite)	Water volume held per pan and daily exchange rate	Water volume at the deepest operating depth of 60 mm: 24.5 L per pan, which lies inside the conical bottom in Section 2.3 (184.7 L on the flat-plan axis in Table 3); exchange ≥20% of the water actually held, 0.8 L·d^−1^ per pan at 20 mm and 4.9 L·d^−1^ at 60 mm	Engineering assumption, to be confirmed by the operator (Section 4.5(4))
Room temperature 22–28 °C, relative humidity > 80%	Exposure classification and corrosion rate	Indoor humid atmosphere; unprotected Q235 steel loses 0.05–0.15 mm·a^−1^	Engineering assumption; GB 50017 [34]
Dissolved oxygen above the species requirement	Water surface area per tier; absence of stagnant zones	Geometric constraint: identical pan geometry on all three tiers, no numerical input	Assumption
Cocoon deposition in moist substrate above the waterline	Additional vertical load; upper bound on the operating water depth	25 mm moist layer, wet bulk density 600 kg·m^−3^, over 30% of the pan plan area → 13.9 kg per pan, 41.6 kg over the three pans (25 mm × 600 kg·m^−3^ × 0.30 × 3.0791 m^2^ = 13.86 kg)	Moisture content measured for broodstock substrate, 70–75% [35]; dry bulk density 165 kg·m^−3^ follows from it; layer thickness an engineering assumption
Uniform conditions across tiers	Frame stiffness expressed as water-depth uniformity	Deflection utilisation ≤100% of *L*/200 in the design case	Engineering criterion; Section 2.8

Note: The substrate entry yields 13.9 kg per pan, and one tier carries one pan, so the machine carries 41.6 kg, which is the allowance that enters Equation (1).

**Table 3:** Water mass and total vertical load as functions of the water level. The rack contribution is three units of the recommended scheme, 3 × 101.34 kg; the substrate column adds the 41.6 kg allowance in Section 2.2. The level is measured on the flat-plan axis of Equation (1), that is, as *A_in_*·*h*, and it is a load parameter rather than a depth: the lowest 160 mm is conical, so *A_in_*·*h* exceeds the volume the pan holds, by 7.5-fold at 60 mm, where the cone carries 24.5 L and the machine 6.09 kN rather than the 10.80 kN of the table. The rows are retained on this axis because the design case and the admissible levels in Section 3.9 are tabulated on it; the loads carried over the operating range are quoted from the cone volume in Section 2.3, and the design case is the measured envelope of 1417.3 L rather than the 1678 kg the expression would yield at 545 mm.

Flat-Plan Level *h*/mm (*V*/*A_in_*)	Flat-Plan Water *A_in_*·*h* per Pan/kg	Water, Three Pans/kg	*W* with Substrate/kN	*W* Without Substrate/kN
20	61.6	184.7	7.18	6.77
60	184.7	554.2	10.80	10.39
100	307.9	923.7	14.43	14.02
200	615.8	1847.4	23.49	23.08
300	923.7	2771.2	32.55	32.14
400	1231.6	3694.9	41.61	41.20
460 (design case: envelope capacity, 1417.3 L = 460.3 mm)	1417.3	4251.9	47.08	46.67

Four features in Table 2 deserve emphasis. First, it separates inputs fixed by the species or the process from values that are engineering assumptions adopted here and still to be confirmed by the operator: the exposure classification, the substrate allowance, the minimum clear opening and the dissolved-oxygen requirement belong to the second group and are labelled as such, so that the design can be re-evaluated when measured data become available. Second, the operating water depth is the primary design variable in the load sense: bounded above by the requirement that the substrate remain above the waterline, it determines the water volume and, hence, the mass carried per tier, so a rack design that does not state the depth at which it is safe is meaningless, and the results below are presented as a function of depth rather than for a single assumed case. Third, the substrate layer is a structural load and not a fixture: it is carried in addition to the water and, sitting above the waterline, raises the centre of gravity of the tier load; in the absence of a measured substrate mass, a conservative allowance is adopted and its influence on the safety factors is quantified in Section 3.9 rather than assumed negligible. Fourth, the bedding is a materials choice, as well as a load, because the medium that sets the bulk density is the medium the animals actually receive: two commercially processed media with electrical conductivities of 9.47 and 10.14 dS·m^−1^ led to complete broodstock mortality within two to four days and no cocoons at all, against 50–60% survival over 75 days with normal cocoon output on unamended peats at 3.69 and 1.19 dS·m^−1^ [35]. The substrate must, therefore, be specified together with its measured density, and Section 4.4 returns to what that implies for other species.

### 2.3. Load Model: Measured Pan Geometry, Water Level and Total Vertical Load

Pan geometry, as built. The pans are not the shallow cylinders the rendering suggests. Each is a moulded GFRP vessel with a cylindrical barrel of 2.00 m outer diameter and 385 mm height standing on a conical bottom that tapers from the same 2.00 m at its upper edge to 0.40 m over 160 mm, so the rim sits 545 mm above the lowest point. Two capacities follow from those measurements, and they are not the same number. The envelope of the external geometry is 1417.3 L—1209.5 L in the barrel and 207.8 L in the cone—and it is what a tape measure returns. The water the pan actually holds is smaller due to the material composition of the vessel: 67.09 kg of laminate at 2600 kg·m^−3^, the density in Table 1, occupies 25.8 L, so the wetted capacity is 1391.5 L, 1.8% below the envelope, and this corresponds to a 4.6 mm moulded wall. The design case adopts the envelope, for a reason stated rather than implied: it is the only one of the two that follows from measurements alone, the wall thickness being inferred from the pan mass rather than measured, and it errs on the heavy side. Section 3.10(4) reports what the wetted capacity produces, because 1.8% is of the same order as the strength margin that decides the section, and the fill test in Section 4.5(vi) closes the difference. The plan area of the full section is *A_in_* = π*D_in_*^2^/4 = 3.0791 m^2^ for *D_in_* = 1.98 m, the nominal circle is 10 mm inside the rim, so a water layer of 1 mm over that area is 3.079 kg per pan and 9.237 kg over the machine. Because the cone occupies the lowest 160 mm, the volume held at a given level is smaller than *A_in_*·*h*, and the two diverge as the level falls: the cone holds 4.0 L at 20 mm, 24.5 L at 60 mm and 70.2 L at 100 mm, against 61.6, 184.7 and 307.9 L on the flat-plan axis, so that expression overstates the water 7.5-fold at the deepest operating depth and by 18% at the rim, where it yields 1678 kg against the 1417 kg of the external envelope. The flat-plan expression is retained as a conservative envelope and as the axis on which the design case and the admissible levels in Section 3.9 are tabulated, but it is not the water the pan holds over the operating range, and the load quoted there is taken from the cone volume. The design case below uses the measured envelope rather than the flat-plan one, and its equivalent depth on this axis is 1417.3/3.0791 = 460 mm. The 13.9 kg substrate allowance in Section 2.2 and the 67.09 kg pan mass are additional to the water in every case. These quantities are summarised in Table 3.

Total vertical load. With *n_vessel_* = 3 pans and three rack units of mass *m_rack_* each, the total gravity load carried by the machine is

(1) $$W(h)=g[\rho_{w}A_{in}hn_{vessel}+m_{vessel}+m_{substrate}+3m_{rack}]$$

where *h* is the water level above the lowest point of the pan, *m_vessel_* = 201.27 kg, the substrate allowance is 41.6 kg when present, and *m_rack_* is the unit mass of the scheme under consideration, taken from Table A1. Table 3 evaluates Equation (1) over the range used in Section 3.9 and in the design case.

Two load levels, and one of them is an accident. The machine has a declared operating range of 20–60 mm of water, at which the load is small: 5.5–6.1 kN with the substrate and 5.1–5.7 kN without it, the water being that inside the conical bottom and not the flat-plan envelope, which would put the same range at 7.2–10.8 kN. The exchange duty in Table 2 is 20% of the water actually held rather than a fixed volume, so it scales with the depth—0.8 L per pan per day at 20 mm and 4.9 L at 60 mm—and no depth in the range is excluded by it. It also has a credible accidental level, confirmed with the operators and supported by the level-limit protection in the operating procedure: a stuck inlet valve, a blocked outlet during cleaning or an overflow during washing puts a pan at its rim. The rack is dimensioned on that accidental level, because a water-holding structure that is checked only at its operating depth is checked at the one condition in which nothing can go wrong. The design case is, therefore, all three pans standing full to their rims—the accident applied to every tier at once, which is the credible form of it here because the three pans are fed from one supply line under a single level-limit protection, so a failure of that protection fills all three. Its water is 3 × 1417.3 L = 4252 kg, equivalent to a layer of 460 mm over the plan area of each pan; with the substrate, the recommended scheme’s own mass and the three pans, it yields *W_d_* = 47.1 kN. No overload factor is applied on top of it: a 1.5× factor on an accidental level would be a second accident, and the case is already the credible maximum. The envelope is conservative in a way worth stating. Were the accident confined to one pan, the water would be 1417 kg and *W_d_* 19.3 kN, and the strength check would be unchanged, because the peak stress is a per-pan quantity scaled by the water in that pan: *SF_y_* stays at 2.09 for the recommendation and 1.48 for the candidate, and only the stability margins and the shape of the chart in Section 3.9 move. The recommendation, therefore, does not rest on the three-pan reading.

**Figure 2:**
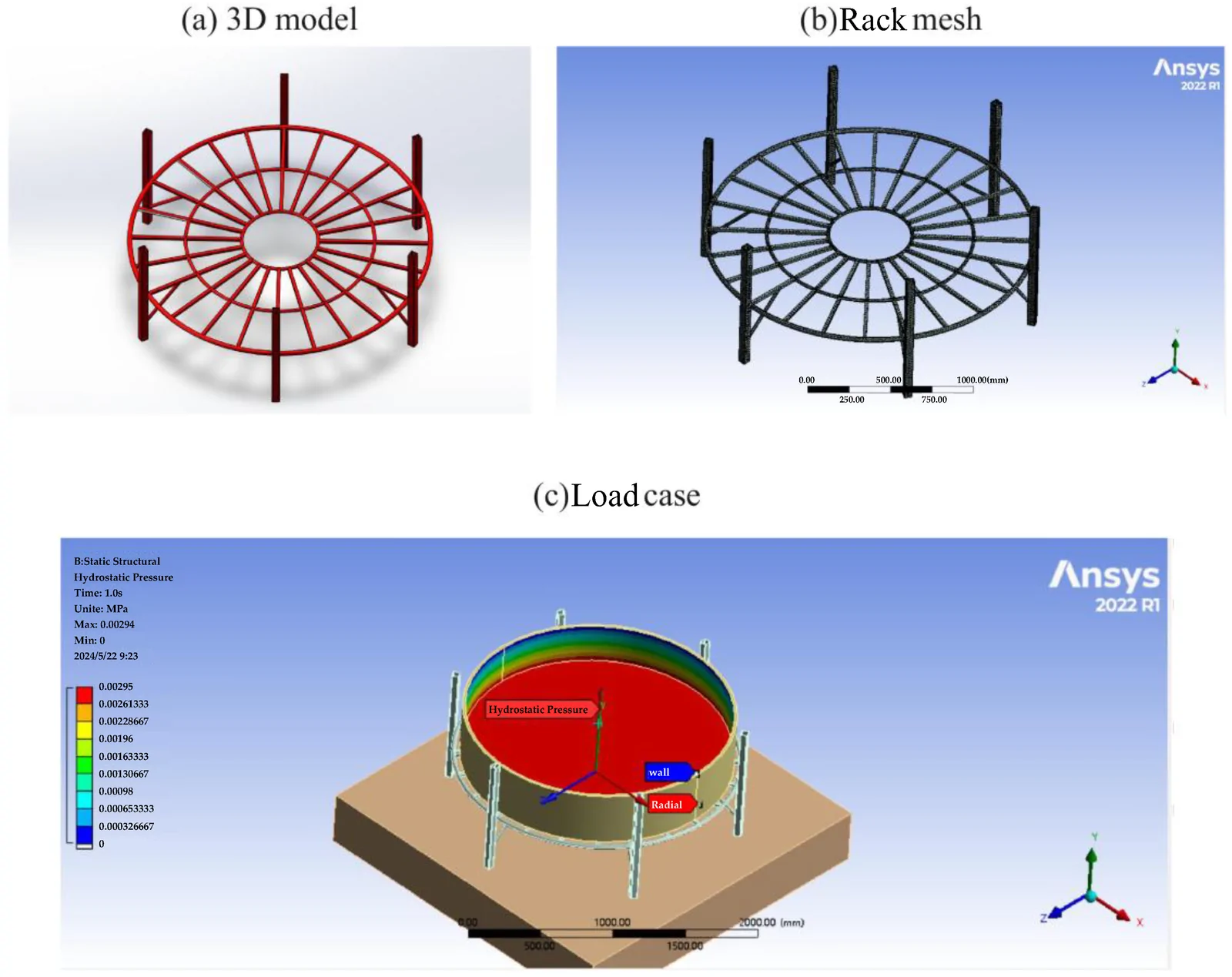
Finite element model: (**a**) 3D model; (**b**) rack mesh; (**c**) applied load, a maximum of 0.00294 MPa, that is, a 300 mm water column, which is the quantity from which the load basis in Section 2.3 is read.

The load basis of the finite element results and what it does and does not establish. The archived solutions were obtained with hydrostatic pressure applied to one pan up to the rim of the pan as modelled, and the elevation of that free surface was not reported in the original work. It can be read off the pressure contour: the legend in Figure 2c shows a maximum of 0.00294 MPa, and 2.94 kPa is the pressure at the base of a 300 mm water column, so the modelled pan is 300 mm deep and the free surface sits at its rim. The inference is single-valued rather than a best fit: the modelled pan is flat-bottomed, so its maximum pressure is the pressure at the base, and 2.94 kPa admits one depth and no other. The model represents one tier, the pan being supported on that tier’s rings and the tier’s tubes standing on the concrete floor, so it carries one pan and not three. Two consequences follow, and both are stated rather than assumed as follows:

The strength and deflection results are scaled to the design case by the ratio of the water carried, 1417.3/815.2 = 1.739, where 815.2 kg is the water in the modelled pan at 300 mm over its 2.7172 m^2^ plan area. The scaling is exact for the hydrostatic part of a static linear-elastic solution, and it is applied to the whole solution, but the dead load—the pan’s 67.09 kg and the rack’s own 101.34 kg—does not grow with the water, so the scaled value is an upper bound on the design-case stress and every strength margin below is the lower end of an interval. The other end follows from carrying the dead load at its own mass: the load ratio is then (1417.3 + 168.4)/(815.2 + 168.4) = 1.612, *σ* = 104.2 MPa and *SF_y_* = 2.26 instead of 112.3 MPa and 2.09. The peak stress sits in the deck under the water load, so the true value is nearer the upper bound and the checks use it; Section 3.10(4) shows that the section chosen is the same at either end.
The modelled pan is smaller than the as-built one (1.86 m inner diameter against 1.98 m) and is flat-bottomed. The support radii are unchanged, so the mesh stress—where the peak occurs—is governed by the water mass transferred to the rings, which the scaling preserves; the pan’s own plate stress is not resolved by this model, and Section 4.5 lists the as-built pan as the geometry to be re-solved first.

There is no free parameter left in this chain: the archived load is now known from the contour legend, the modelled geometry is known, and the design case is the measured envelope in Section 2.3. Appendix C records this derivation.

What depends on the design case and what does not. The ranking of the 36 schemes, their relative masses and the weighting results in Section 2.6 are unaffected, because they compare schemes at one load. What the design case fixes is the absolute margin, and with it the admissible water level of each scheme, which is the quantity Section 3.9 tabulates.

### 2.4. Finite Element Model

Three-dimensional parametric modelling was performed in SolidWorks 2016, and the geometry was imported into the Static Structural module in ANSYS Workbench, where the finite element analysis (FEA) was carried out. The model is one-tiered: one pan on its three rings and 24 beams, the tier’s tubes landing on the concrete floor, which is the load path of the top unit; the two units below carry an additional axial load from above, which Section 2.8(2) checks separately. For contact definition, a frictional contact with a coefficient of 0.2 was assigned between the water tanks and the rack, as well as a rough contact between the rack and the concrete floor. Tetrahedral elements were used, with mesh sizes of 50, 20 and 100 mm for the tanks, rack and concrete floor, respectively, generating 184,693 nodes and 94,468 elements. The solver settings are summarised in Table 4, and the finite element model, mesh and applied load are shown in Figure 2.

Convergence. One refinement step was performed for the maximum total deformation, and the result changed by 0.30572%; two meshes bound the discretisation error at that level but do not constitute a convergence study, and the stress, being the more sensitive quantity, is treated accordingly below. The peak equivalent stress carries a different status. It is located at a geometric discontinuity in the mesh structure, where the solution is more sensitive to the element size than the global deformation is, so it is given two well-defined roles. First, it serves as a comparative indicator: all 36 schemes were solved on the same mesh with the same element-size distribution, which makes their ranking by peak stress internally consistent and is what the selection procedure in Section 3.4 requires. Second, where an absolute strength margin is required, it is computed from a peak stress anchored on the solved response of the round-bar family and carrying a stated uncertainty band (Section 3.10) rather than from the raw finite element maximum. A local mesh refinement at that location is step (ii) of the verification protocol in Section 4.5.

Provenance and availability of the finite element data set. The 53 finite element solutions analysed here are the author’s earlier unpublished work on this rack, carried out with the geometry described in Section 2.1. They were produced before the husbandry requirements in Section 2.2 had been assembled, which is why the water depth and water mass were not recorded in that work; the load case they represent is recovered from the pressure contour in Section 2.3. The 53 solutions are reproduced in full in Table A1 and Table A3, and the model files, mesh and solver settings are available from the corresponding author on reasonable request. Where this paper refers to “the earlier design” or “the original design”, it refers to this unpublished work and to no third party’s published result.

The load case analysed here is the gravity load of Equation (1) in the design case in Section 2.3. Wind, seismic action, maintenance live loads, tray-handling loads and water-level fluctuation lie outside it, and Section 4.5(4) quantifies each of them as an addition to *W*.

### 2.5. Parametric Design Space

The finite element results show that the mesh frame is an integrated welded assembly that would be costly to reconfigure, whereas the square tubes and round bars are standard sections whose size and number are readily adjusted. Two groups of geometric parameters were, therefore, selected as design variables.

**(1) Square-tube subsystem.** Edge length *S* ∈ {20, 25, 30, 35, 40, 45, 50, 55, 60} mm (9 levels, wall thickness fixed at 2.5 mm) and tube number *n* ∈ {8, 6, 4, 3} (4 levels), resulting in 9 × 4 = 36 full factorial design points. Scheme index *i* = 4(*r* − 1) + *c*, where *r* and *c* are index edge length and tube number, respectively.

**(2) Round-bar subsystem.** With the square tubes fixed at 25 × 25 mm and *n* = 8, diameter *d* ∈ {5, 10, 15, 20} mm and bar number *k* ∈ {8, 6, 4, 2} result in 16 design points, to which one further configuration without round bars was added, resulting in 17 in total.

### 2.6. Multi-Criteria Decision-Making Model

**(1) Mandatory constraints are applied before any ranking.** The three evaluation criteria—mass, *m*, (kg), maximum total deformation, *δ*, (10^−2^ mm) and maximum equivalent stress, *σ*, (MPa)—are all cost-type, but they are not interchangeable. Strength, stability and deflection are *requirements*; the relative importance attached to mass, deformation and stress is a *preference*. The two are separated here. A scheme is admitted to the decision only if it satisfies, at the operating load, *W*, of Equation (1),

(2) $$SF_{y}=\frac{\sigma_{y}}{\sigma }\geq 2.0,SF_{b}=\frac{\Sigma P_{cr}}{W}\geq 2.0,\delta \leq \frac{L_{span}}{200}=9.80\;\mathrm{mm}$$

The three thresholds are the customary values for agricultural steelwork and are treated as mandatory; they are not traded against one another. They are also applied to an accidental action rather than to a persistent design situation, and that is deliberately stricter than the combination rules require: GB 50068 [36] provides an accidental combination of a reduced importance factor and takes the accompanying actions at their frequent rather than their design values, whereas the factors above are carried unchanged into the pan-full case. The margins quoted in this paper are, therefore, lower bounds on the code comparison, as well as on the load, and the conservatism is stated here rather than left for the reader to discount. Within the feasible set, the objective is the **minimum rack mass**, because the mass governs the cost in this application. The weighted ranking is used afterwards and only to compare the feasible schemes on their non-mass attributes and to document the preference structure: if the screen removes a scheme, the weighting cannot reintroduce it, so the reader can see how much of the recommendation rests on computation and how much on judgement. All 36 square-tube schemes are feasible at the *operating* depth, so none is excluded there, but at the accidental design case in Section 2.3, the screen removes eight of them (Section 3.4), and it remains binding at larger depths, which is what determines the answer there. The screen is applied on the nominal wall; the corrosion allowance is not stacked on top of it, for the reason Section 3.8(4) provides—an accidental action and a ten-year service allowance are separate actions—and its effect is reported instead as the reduction in the admissible water level in Section 3.10(5).

**(2) Range normalisation of the criteria.** For criterion *j*, with *x_max_* and *x_min_*, the maximum and minimum over the design set,

(3) $$r_{ij}=\frac{x_{max,j}-x_{ij}}{x_{max,j}-x_{min,j}}$$

**(3) Normalisation convention V: vector normalisation with a cost-type ideal.** The criterion matrix is normalised to the unit column norm,

(4) $$v_{ij}=\frac{x_{ij}}{\sqrt{\sum_{i=1}^{n}{ij}_{2}^{x}}}$$

the weighted matrix is *ṽ_ij_* = *w_j_ v_ij_*, and because every criterion is the cost-type, the positive ideal solution is the column-wise minimum and the negative ideal solution the column-wise maximum:

(5) $$P^{+}=\{{min}_{i}{\overset{\sim }{v}}_{ij}\},P^{-}=\{{max}_{i}{\overset{\sim }{v}}_{ij}\}$$

**(4) Normalisation convention R: range normalisation with a benefit-type ideal.** The normalised values of Equation (3) are used directly, larger being preferred, so that the weighted matrix is *r̃_ij_* = *w_j_ r_ij_* and

(6) $$P^{+}=\{{max}_{i}{\overset{\sim }{r}}_{ij}\},P^{-}=\{{min}_{i}{\overset{\sim }{r}}_{ij}\}$$

In both conventions, the separation measures and the relative closeness are

(7) $$i_{\pm }^{D}=\sqrt{\sum_{j=1}^{m}{\left({\overset{\sim }{z}}_{ij}-j_{\pm }^{P}\right)}^{2}},C_{i}=\frac{i_{-}^{D}}{i_{+}^{D}+i_{-}^{D}}$$

where $\overset{\sim }{z}$ is the weighted matrix of the convention in use, and a larger *C_i_* indicates a better scheme. The two conventions are kept strictly separate so that each positive ideal solution has a defined meaning; the complete *C_i_* values and rankings of all 36 schemes are reported in Table A2, and the disagreement between the conventions is quantified in Section 3.5.

**(5) Entropy weighting (objective).** A probability matrix, *p_ij_* = *r_ij_*/Σ*r_ij_*, is constructed from the range-normalised matrix. The information entropy and entropy weight of criterion *j* are

(8) $$e_{j}=-\frac{1}{lnn}\sum_{i=1}^{n}p_{ij}lnp_{ij},j_{E}^{w}=\frac{1-e_{j}}{\sum_{j=1}^{m}\left(1-e_{j}\right)}$$

The entropy weight reflects the dispersion of the criterion data: greater dispersion means that the criterion carries more decision information and receives a larger weight [21,22]. Because it is a property of the sample, it is re-estimated whenever the data set changes, and the consequences are examined by leave-one-scheme-out analysis in Section 3.5.

**(6) Analytic hierarchy process (subjective), with the judgement basis stated.** The judgement matrix was constructed from engineering intent. Mass carries the highest weight because it drives the direct material cost; peak equivalent stress ranks second because it governs the strength limit state and, through corrosion, the service life; deformation ranks lowest because at these load levels it is an order of magnitude below the *L*/200 limit and its husbandry significance is uniformity rather than safety. Taking

$$A=[\begin{array}{ccc} 1 & 3 & 2\\ \frac{1}{3} & 1 & \frac{1}{2}\\ \frac{1}{2} & 2 & 1 \end{array}]$$

yields *λ_max_* = 3.0092, a consistency index CI = 0.0046 and a consistency ratio CR = 0.0079 < 0.10, so the matrix passes the consistency test, with the subjective weight vector *w*^A = (0.5396, 0.1634, 0.2970)ᵀ. It is a single-expert judgement and is carried as such: every off-diagonal Saaty-scale entry is enumerated over the 17 admissible values and only consistent matrices are retained (Section 3.5).

**(7) Combined weighting.** A linear combination of an objective and a subjective weight vector is admissible only if the two carry information of the same kind, and they do not: the entropy weight measures how much a criterion discriminates among the alternatives actually examined, and the AHP weight states how much it matters when the decision is made. They coincide here in ordering the criteria—mass first, stress second, and deformation third—which is the empirical justification for combining them, and the combination is used as a *reference case* rather than as the definition of the result:

(9) $$w_{j}=\alpha j_{E}^{w}+(1-\alpha )j_{A}^{w},\alpha \in [0,1]$$

For completeness, the AHP-only and entropy-only rankings are reported alongside the combined ranking rather than being folded into it, and α is swept over its whole range in Section 3.5.

**(8) Gray relational analysis (cross-check).** A difference sequence was constructed from the complement of the range-normalised matrix, gray relational coefficients were computed with a distinguishing coefficient of 0.5, and the coefficients were combined using the combined weights to yield the gray relational grade, *G_i_*, which is compared with the TOPSIS result in Section 3.5.

**(9) Sensitivity protocol.** Four distinct sources of uncertainty are examined, because sweeping the combination coefficient, α, alone would test only one of them.

*(a) Preference uncertainty.* The AHP judgement is a single-expert assessment, so the whole admissible space of consistent judgement matrices is explored. Each of the three upper-triangular entries is allowed to take any of the 17 Saaty-scale values, yielding 17^3^ = 4913 matrices; the consistency ratio is recomputed for each, and the matrices with CR ≤ 0.10 are retained. The distribution of the top-ranked scheme and of the rank of the reference scheme over that admissible set is reported.

*(b) Data uncertainty.* The entropy weights are re-estimated 36 times, each time omitting one scheme from the set, and the resulting spread of weights and of the top-ranked scheme is reported. This tests whether the entropy weights are controlled by a single influential alternative.

*(c) Criterion-set uncertainty.* Each of the three criteria is deleted in turn, and the weights are rebuilt from the remaining two; the resulting ranking is reported. This tests whether the conclusion depends on a criterion that has been included without justification.

*(d) Alternative-set and normalisation uncertainty.* Four subsets of the design space are formed—the four lightest schemes removed, the four heaviest removed, the four highest-stress schemes removed, and the whole 20 mm or 60 mm edge-length level removed—and each is re-ranked with re-estimated entropy weights. In addition, the entire ranking is recomputed under normalisation convention R in Section 2.6(4) and compared with convention V using the Spearman rank correlation coefficient.

### 2.7. Response Surface Regression and Analysis of Variance

Because a full factorial experiment with a single replicate provides zero residual degrees of freedom, the interaction cannot be tested directly. Two complementary treatments were, therefore, adopted.

**(1) Two-factor additive ANOVA and what its residual can and cannot show.** Edge length, *S*, was treated as a 9-level factor and tube number, *n*, as a 4-level factor, and an additive model without interaction was fitted. The residual sum of squares then contains the interaction together with lack of fit and any pure error, with (9 − 1)(4 − 1) = 24 degrees of freedom, allowing *F* tests of the two main effects:

(10) $$SS_{A}=4\sum_{i=1}^{9}({\bar{y}}_{i\cdot }-\bar{y}{)}^{2},SS_{B}=9\sum_{j=1}^{4}({\bar{y}}_{\cdot j}-\bar{y}{)}^{2},SS_{E}=SS_{T}-SS_{A}-SS_{B}$$

Contributions are reported as *SS*/*SS_T_*. Because the underlying experiment is a full factorial solved once per point, there is no independent replicate and, therefore, no pure-error estimate; the residual cannot be decomposed into interaction and error, and an *F* test on the residual mean square tests the two main effects against a mixture of interaction, lack of fit and noise. The *F* tests in Table 5 are reported on that understanding, and no claim of statistical significance is attached to the residual itself.

**(2) Stratified simple effects, as an independent check on interaction.** Because the additive ANOVA cannot isolate the interaction, the effect of edge length is computed separately at each level of tube number—that is, as a set of simple effects rather than a pooled main effect. If edge length acted additively, its effect would be the same at every tube count; the ratio of the largest to the smallest simple effect is, therefore, a direct, assumption-light measure of interaction that does not depend on a sum-of-squares decomposition. A second, independent indication is obtained from the fitted response surface: the fraction of *SS_T_* carried by the interaction term *ab* in Equation (11) can be compared with the residual of the additive model, and if the interaction term accounts for most of that residual the two analyses are mutually consistent.

**(3) Second-order response surface regression.** Using the coded variables *a* = (*S* − 40)/20 and *b* = (*n* − 5.25)/2.5, the full quadratic model

(11) $$y=\beta_{0}+\beta_{1}a+\beta_{2}b+\beta_{11}a^{2}+\beta_{22}b^{2}+\beta_{12}ab$$

was fitted, and leave-one-out (LOO) cross-validation was used to estimate the predictive coefficient of determination, *R^2^_pred_*. The response surface is used here as a description of the solved data rather than as a surrogate for design, with its two roles separated by quantity: it reproduces mass to *R^2^_pred_* = 0.999957 and the peak stress to *R^2^_pred_* = 0.5939 (Section 3.2). Every design point reported in this paper was solved.

### 2.8. Structural Checks: Buckling Regime, Corrosion Allowance and Serviceability

**(1) Buckling regime and the effective length factor.** The square tubes are compression members welded to the annular rings near both ends. The section properties are

(12) $$I=\frac{S^{4}-(S-2t{)}^{4}}{12},r=\sqrt{I/A},A=S^{2}-(S-2t{)}^{2},\lambda =\frac{KL}{r}$$

The elastic–inelastic transition slenderness is *C_c_* = √(2π^2^*E*/*σ_y_*) = 129.61. For *λ* ≥ *C_c_*, the Euler formula, *σ_cr_* = π^2^*E*/*λ*^2^, applies; for *λ* < *C_c_*, the Johnson parabola

(13) $$\sigma_{cr}=\sigma_{y}[1-\frac{\sigma_{y}\lambda^{2}}{4\pi^{2}E}]$$

is used. The AISC 360 column curve [37], *F_cr_* = 0.658^(*λ_c_*^2^)·*σ_y_*, with *λ_c_* = (*λ*/π)√(*σ_y_*/*E*) was adopted as a cross-check that accounts for residual stresses and initial crookedness. The critical load is *P_cr_* = *σ_cr_*·*A*.

The effective length factor is the largest single lever in this chain. Each column is a vertical tube of length *L* = 890 mm, welded to the mid-pan annular ring above and to the base ring below, so the physical restraint lies between *K* = 0.5 and *K* = 1.0, and no rotational stiffness has been measured; *K* is, therefore, a value assigned rather than described, and the assignment is deliberately pessimistic. The analysis uses *K* = 1.0, the fully pinned end, with *K* = 0.85 and 0.70 reported as the plausible range of partial to substantial fixity (Section 3.8(1)). The lever is large: *P_cr_* of the 20 × 20 mm section rises from 22.51 kN at *K* = 1.0 to 32.00 kN at *K* = 0.70, an increase of 42.2%.

**(2) Distribution of the vertical load between the columns.** The gravity load reaches the columns through the mesh deck and the annular rings, and the base plate is bolted to a concrete floor far stiffer than the frame, so the axial force per column is taken as *W*/*n*. Where the column count does not divide the ring positions or where one column is shortened by fabrication tolerance, this is optimistic; its influence is bounded by re-evaluating the stability safety factor with the most heavily loaded column at 1.2 times the mean (Section 3.8(1)). The eccentricity of the vertical load relative to the column axes is not modelled separately, because the global bending it induces—where the peak stress occurs—is already contained in the finite element solution used for *SF_y_*.

**(3) Utilisation ratios.** The strength utilisation is *σ_max_*/*σ_y_* and stiffness utilisation *δ*/(*L_span_*/200), with *L_span_* the largest ring diameter of 1960 mm, so the allowable deflection is 9.80 mm, and the stability utilisation is the column axial force over *P_cr_*. Stiffness is deliberately reported as a ratio and not in millimetres: at the depths used in leech culture, a deflection of a few tenths of a millimetre matters only through the uniformity of the water depth between tiers, an effect quantified by comparing the deflection with the depth itself (Section 3.8(3)).

**(4) Corrosion allowance, design life and exposure assumptions.** The rearing room is assumed held at 22–28 °C and above 80% relative humidity with the frame splashed at every water exchange; on the ISO 9223 scale [38], this is corrosivity category C3 and, following GB 50017 [34], an indoor humid atmosphere. A design life of 10 years is adopted as an engineering assumption to be confirmed by the operator. For an unprotected Q235 surface, the uniform corrosion rate is 0.05–0.15 mm·a^−1^, so a 2.5 mm wall loses 0.50–1.50 mm over that life and becomes unserviceable; the checks are made against the 235 MPa nominal minimum in Section 2.1, so no further reduction for grade has to be carried here. For a hot-dip galvanised surface, 65–85 μm of zinc at a loss rate of 3–10 μm·a^−1^ yields a protection life of about 6.5–28 years, and that is a range of conditions, not a guarantee: the 10-year life is supported only if the coating is at least 65 μm and is maintained, a condition stated in Section 4.5(7) rather than assumed. The structural consequence is evaluated by reducing the wall to *t_eff_* = 1.75 mm—a one-sided loss of 0.75 mm—and recomputing the section properties from Equation (12). The 0.75 mm point is the mid-point of the 0.50–1.50 mm range, and the unprotected rate spans over ten years, which is taken on one face because the bore of a closed tube is not wetted and taken as a local-loss allowance for a coating breach rather than uniform thinning, since the coating protects the member for 6.5–28 years, and any breach is repaired at the next annual inspection. The coating mass follows from

(14) $$f_{plate}=\frac{2t_{Zn}\rho_{Zn}}{t\rho_{s}},f_{bar}=\frac{4t_{Zn}\rho_{Zn}}{d\rho_{s}}$$

So the plate value is 5.82% at *t* = 2.5 mm and independent of the section size, whereas the bar value is 5.82% at 5 mm, 2.91% at 10 mm, 1.94% at 15 mm and 1.46% at 20 mm. Table A4 resolves the coating member group by member group; the masses quoted in this paper are bare-steel masses, and applying the 5.82% plate value to every member would overstate the coating on the 20 mm bars by a factor of four.

## 3. Results and Analysis

### 3.1. Finite Element Results for the Baseline Design

**Figure 3:**
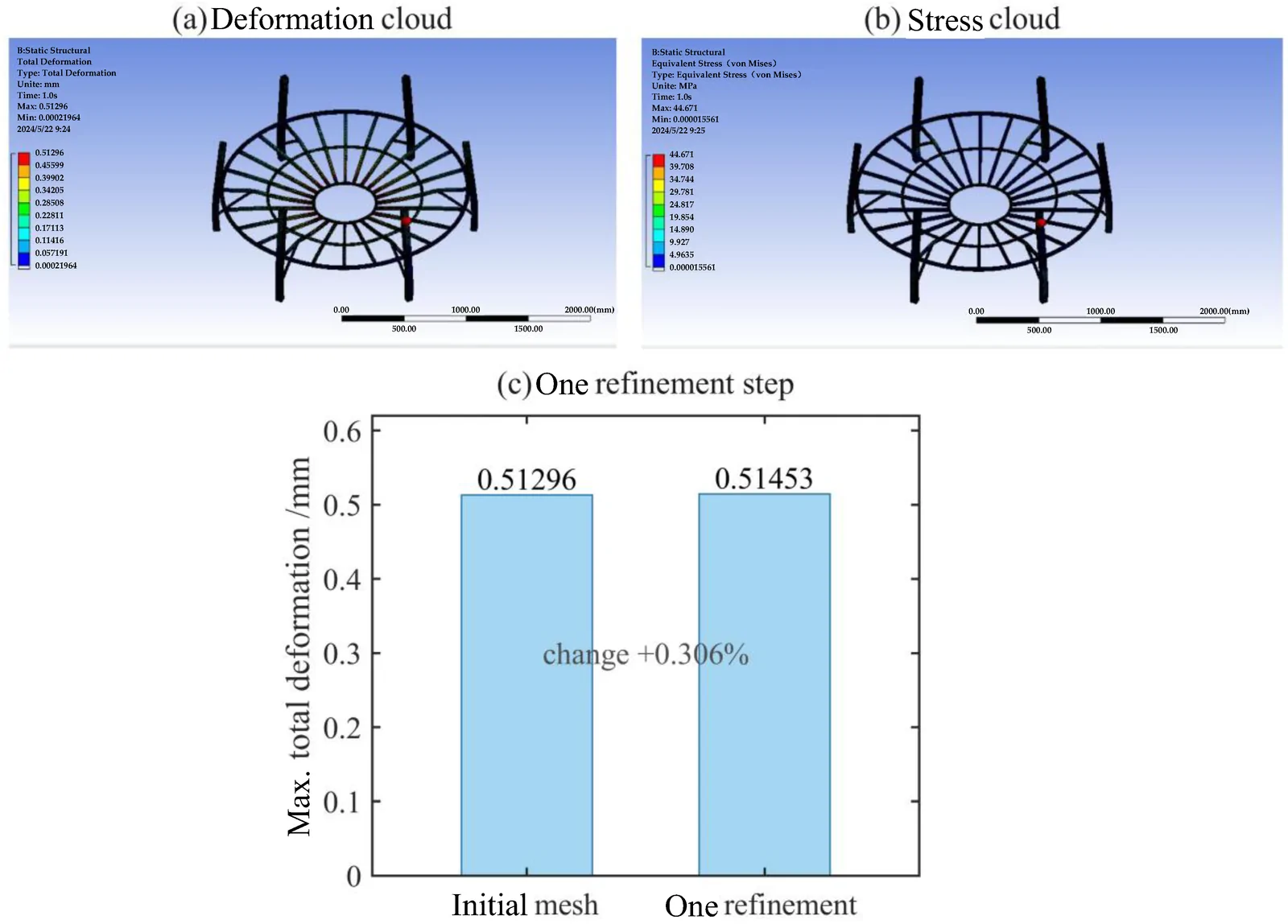
Baseline finite element results: (**a**) total deformation; (**b**) equivalent stress; (**c**) mesh refinement (one refinement step).

For the baseline design (six 50 × 50 × 2.5 mm square tubes and eight 20 mm round bars), the total mass is 124.14 kg, the maximum total deformation 0.513 mm, located at the middle of the inner ring, and the maximum equivalent stress 44.67 MPa, located at the centre of the mesh deck. Substituting the section properties into Equations (12) and (13) with *λ* = 45.83 < *C_c_* = 129.61 shows that the members are intermediate columns, with a Johnson critical stress of 220.31 MPa and a critical load of 104.65 kN per tube. Relative to the 235 MPa nominal minimum, the baseline has a yield safety factor of 5.26, an ultimate safety factor of 10.30 and a material utilisation of 19.0%. Both responses are, therefore, between a factor of five and a factor of ten below the capacity of the members, which is the quantitative form of the redundancy that the earlier design work had inferred only from the appearance of the contours. The total deformation field, the equivalent stress field and the single refinement step used to check the deformation are shown in Figure 3.

### 3.2. Parametric Response of the Square Tubes: Analysis of Variance, Interaction Structure and Response Surfaces

**Figure 4:**
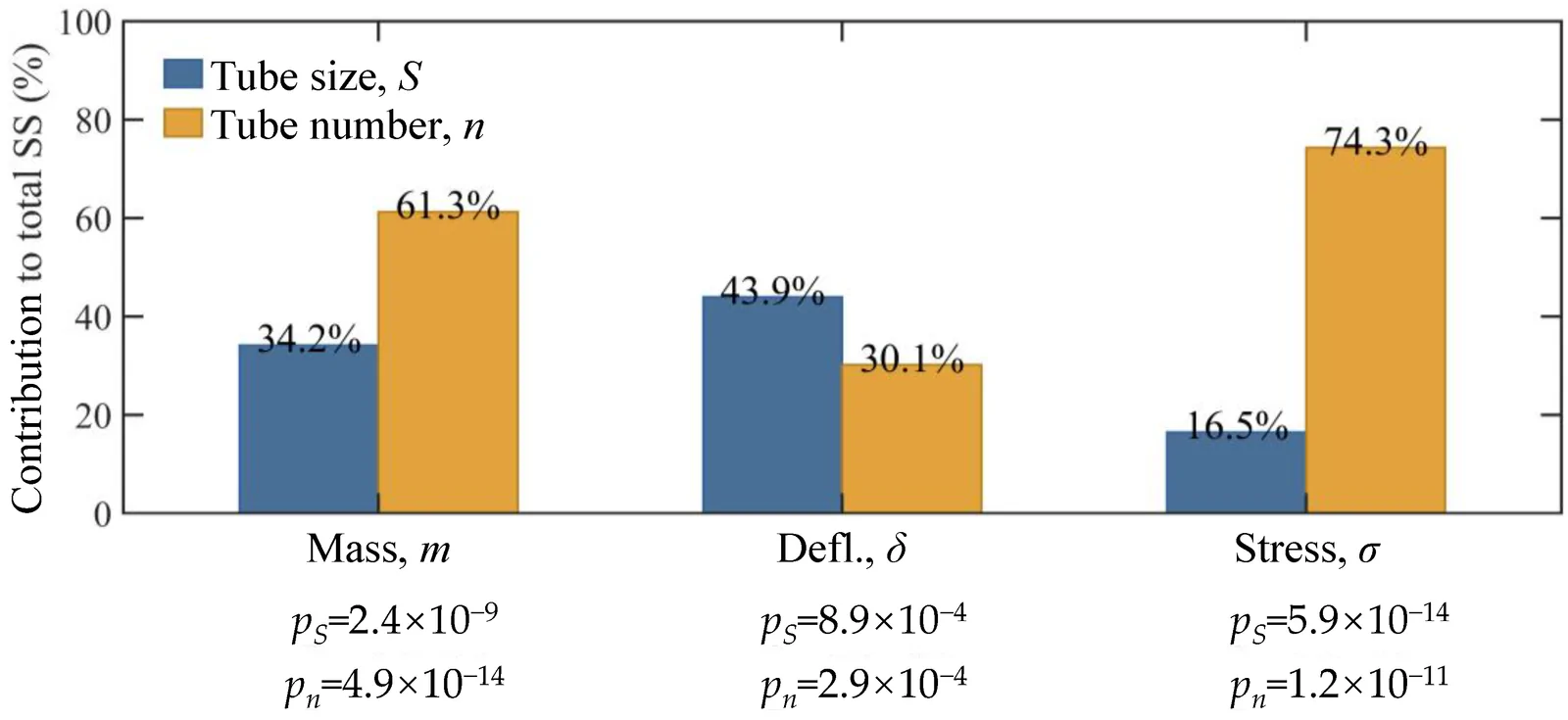
ANOVA contributions of the tube edge length and tube number to the mass, deformation and stress.

The finite element results for the 36 square-tube design points are listed in Table A1. A two-factor additive analysis of variance following Equation (10) was carried out on the three responses; the results are presented in Table 5, and the factor contributions are compared in Figure 4.

Three statements follow.

First, for the mass, the tube number contributes 61.26% and the edge length 34.16%, both of which are significant: removing two tubes lowers the mass by about 8.1 kg at the 50 mm section (3.9 kg at 20 mm), whereas shortening the edge by 5 mm lowers it by about 2.8 kg on average—the ordering expected from length × area × density.

Second, for the maximum equivalent stress, the tube number dominates (74.32% against 16.52%). Enlarging the section, therefore, cannot by itself lower the peak stress: the number of load paths fixes the unsupported span of the annular frame, and the span, not the section, controls the bending stress.

Third, for the deformation, the two contributions are comparable (43.93% and 30.13%), and the residual is as large as 25.94%. In a single-replicate factorial, the residual contains interaction, lack of fit and noise together, so a large residual shows the additive model is inadequate without by itself demonstrating an interaction. Two diagnostics that do not depend on the residual decomposition are, therefore, used.

The first is a stratified simple-effect comparison: The range of response produced by changing edge length from 20 to 60 mm at a fixed tube number. The deformation range is 8.58 × 10^−2^ mm at *n* = 8 but 77.26 × 10^−2^ mm at *n* = 3—a ratio of 9.0—whereas the stress ratio is only 1.7 (19.66 against 34.33 MPa) and is inverted for mass (22.80 kg at *n* = 8 against 8.53 kg at *n* = 3). Were edge length additive, its effect on deformation would be the same at every tube count. It is not: the influence of edge length on deformation is conditional on the number of load paths, and the earlier conclusion drawn from line charts—that tube size has little effect on deformation while tube number has a large one—holds only in the *n* = 8 column, where deformation is already saturated by eight supports.

The second diagnostic is the fitted response surface. The second-order model for maximum total deformation, that is, the fitted instance of Equation (11), is

(15) $$\delta =49.14-15.38a-13.78b+16.55a^{2}+8.96b^{2}+15.06ab$$

with *R*^2^ = 0.8797 and *R^2^_pred_* = 0.7945. The interaction term *ab* has a coefficient of 15.06 (*p* = 3.29 × 10^−7^), accounting for 17.05% of the total sum of squares, and *a*^2^ for 11.19% (*p* = 1.05 × 10^−5^). Those are marginal (Type III) sums of squares of the second-order surface and cannot be read as shares of the 25.94% residual of the additive model: the two models are not nested, and the arithmetic shows it—17.05 + 11.19 = 28.24% against a residual of 25.94%. What can be read off is a nested comparison, and it is decisive: adding *ab* to a surface already containing *a*, *b*, *a*^2^ and *b*^2^ lowers the unexplained sum of squares from 29.08% to 12.03% of the total, a partial *F*(1, 31) = 43.9 (*p* < 10^−6^). The two diagnostics agree, in the way Section 2.7(2) anticipated: a structured residual, most of which one interaction term accounts for once the curvature is in the model. The remaining 12.03% is attributed to the lack of fit and to the absence of a pure-error estimate, and it is reported separately from the significance claims for the main effects. The interaction is visible directly in the data as well: at *n* = 8, the eight supports make the frame stiff enough that the edge length has almost nothing left to change, and only at lower tube counts does the section matter.

**Figure 5:**
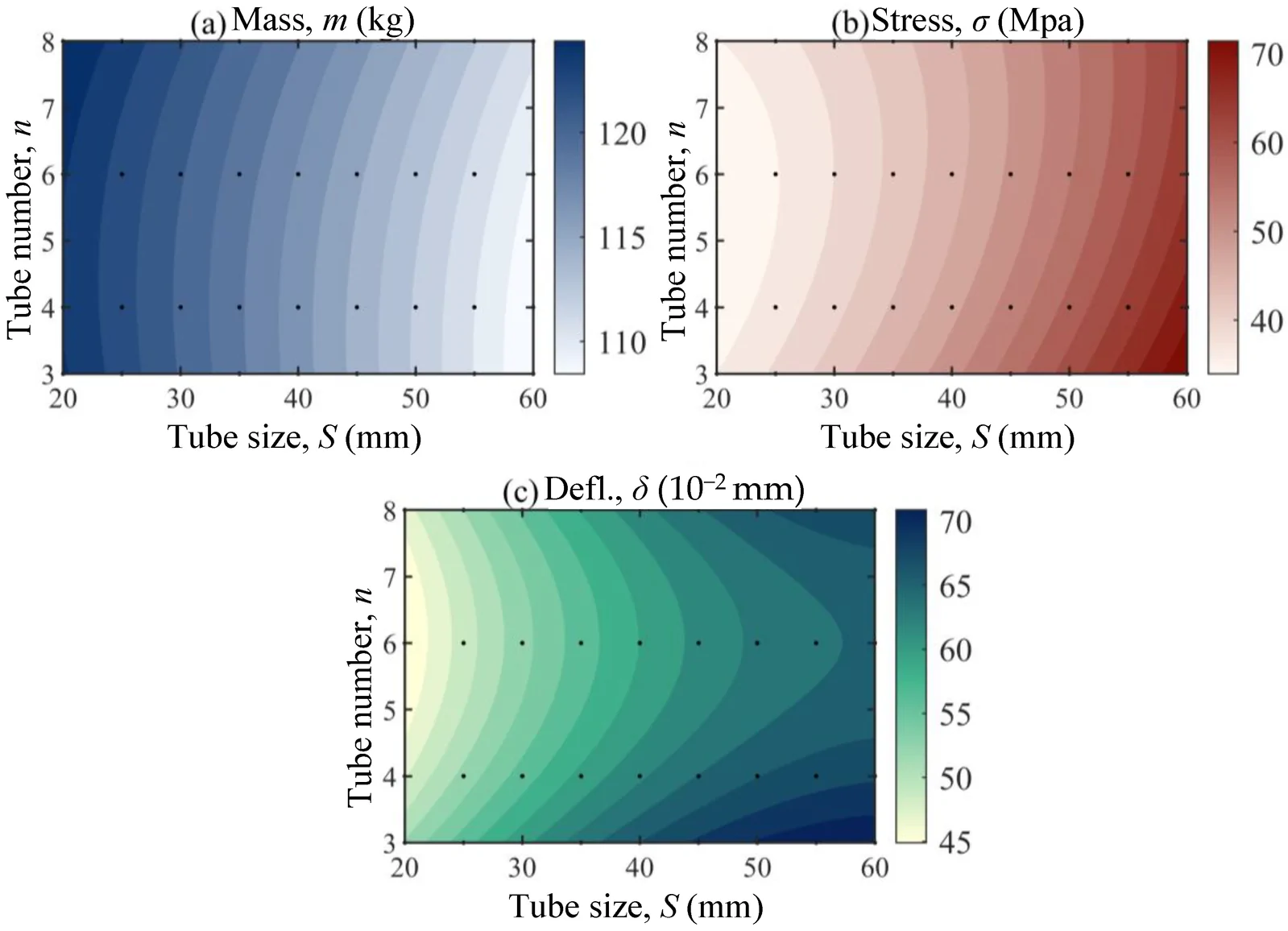
Second-order response surface contours for mass, equivalent stress and maximum deformation (black dots: 36 design points).

The mass surface behaves quite differently: *R*^2^ = 0.999973, with *R^2^_pred_* = 0.999957 and an interaction contribution of only 4.57%, and the remainder is essentially linear—which is what makes the closed-form mass model in Section 3.3 possible. The stress surface reaches only *R*^2^ = 0.7316 (*R^2^_pred_* = 0.5939), the quantitative expression of the local character of the peak stress; a second-order surface is no substitute for the direct finite element re-analysis of that quantity, and none is used here. The coefficient set is presented in Table 6 and the contours in Figure 5.

### 3.3. Mass Decomposition Model

Because the mass response is almost perfectly linear in the two design variables, it admits a closed-form model. Linear regression on the 36 square-tube solutions, with the round-bar contribution written as an explicit term rather than absorbed into the constant, yields

(16) $$m=m_{frame}+n(\alpha +\beta A)+kc_{1}d^{2}$$

where *A* = *S*^2^ − (*S* − 2*t*)^2^ is the tube cross-sectional area in mm^2^, *n* is the number of tubes, *k* is the number of round bars and *d* is their diameter in mm; the fitted coefficients are *m_frame_* = 92.97 kg, *α* = 0.6939 kg, *β* = 7.104 × 10^−3^ kg·mm^−2^ and *c*_1_ = 2.1166 × 10^−3^ kg·mm^−2^, and the fit to the 36 calibration points has *R*^2^ = 0.999946, with a largest single deviation of 0.200%—0.23 kg, on Scheme 1 in Table A1, whose tabulated mass is 115.01 kg—and no other point above 0.14%. The coefficients *α* and *β* are regression coefficients and are unrelated to the coded variables *a* and *b* in Equation (11); *c*_1_ is the fitted coefficient of the round-bar term.

Writing the bar term explicitly is not a cosmetic change. In the earlier formulation, the bar mass was buried in the constant term, so the equation was valid for one bar configuration only, and it also produced a numerical contradiction: evaluated at the configuration of the earlier final design—eight 25 × 25 mm tubes together with eight 20 mm bars, that is, Scheme 13 in Table A3—the earlier equation returned 118.03 kg, whereas the same configuration was quoted elsewhere in that work as 111.74 kg. The difference is exactly the bar substitution (6.77 kg for the eight 20 mm bars against 0.42 kg for the eight 5 mm bars), and it disappears once the term is written out. In the corrected form, the eight-bar calibration set (Schemes 1, 5, 9 and 13 in Table A3) fixes *c*_1_, and the equation then reproduces all 36 tube solutions and the remaining thirteen bar solutions.

The physical interpretation of the coefficients survives and is now unambiguous: *β* = *ρL_eff_* results in an equivalent tube length *L_eff_* = 905 mm, 1.7% away from the 890 mm design value; *α* = 0.6939 kg per tube represents welded joints and similar secondary mass; and *m_frame_* = 92.97 kg is the three-ring welded mesh frame, which does not depend on either design variable.

Validation on schemes withheld from the calibration. The bar term was calibrated on the four eight-bar rows of the round-bar family, so the remaining thirteen rows in Table A3—which vary the bar number from two to six at diameters of 5, 10, 15 and 20 mm and include the control configuration without bars—were withheld and used as a test set. Equation (16) reproduces all thirteen to within 0.047% and to a median of 0.016%, as listed in Table 7. Because those rows change the bar number and, therefore, probe the additivity of the bar term, they constitute a genuine independent test of the form assumed in Equation (16): they exercise the bar term and the constant, which is exactly the range over which the equation is used. Four of the points that the earlier work presented as independent validation were, in fact, members of the calibration set, and one of them was compared against a predicted value that the equation does not yield—the contradiction noted above. The thirteen points in Table 7 are those that genuinely took no part in the fit, and the configuration that exposed the error is now reproduced to 0.016%.

**Figure 6:**
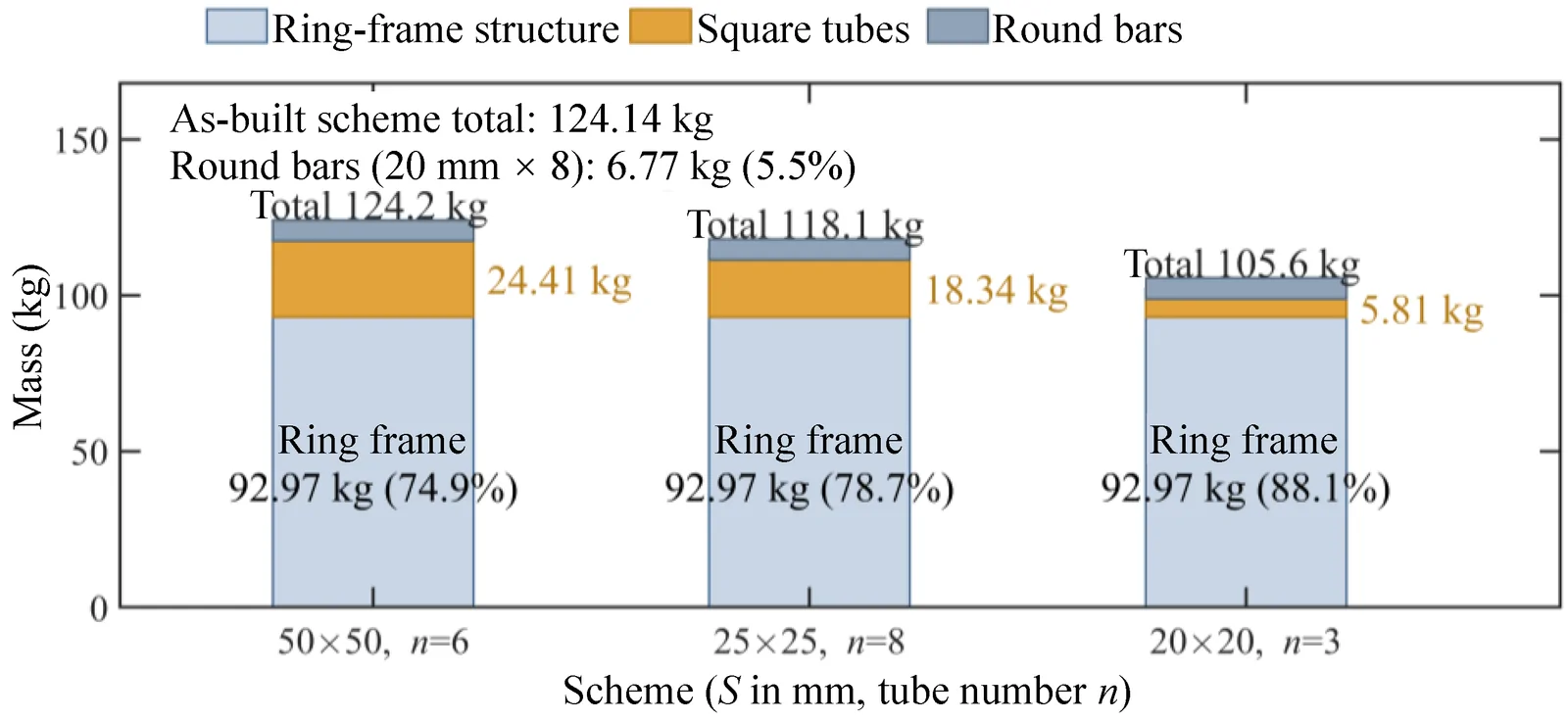
Mass composition of the rack: ring-frame structure, square tubes and round bars.

Decomposition of the baseline mass and the ceiling it imposes. Applied to the baseline, Equation (16) splits the 124.14 kg into a three-ring mesh frame of 92.97 kg (74.9%), six square tubes contributing 24.41 kg (19.7%) and eight 20 mm round bars contributing 6.77 kg (5.5%); the composition is shown in Figure 6. Two consequences follow.

The first is a design ceiling. The optimised subsystem—tubes and bars together—accounts for 25.1% of the rack mass, so no amount of refinement inside it can reduce the total by more than 25.1%, and that figure is an arithmetic limit attained only by deleting the subsystem altogether. Three numbers have to be kept apart, because they answer three different questions: 25.1% is the ceiling—it is what the frame leaves available, and it is not attainable; 20.43% (98.78 kg) is the limit in which the tubes are optimised and the bars contribute nothing, which is physically meaningless but bounds the family from above; and 20.1% (99.21 kg, 20.08%) is what the lightest tube and the lightest bar actually realise at *n* = 3, the subsystem falling from 31.18 kg to 6.24 kg, and it is the only one of the three that corresponds to a buildable rack—although, as Section 3.7 shows, not to an admissible one. A fourth figure is, therefore, needed to close the account: 18.4% (101.34 kg) is what the design that actually passes the screen realises, and the 1.7 points between it and 20.08% are the price of the strength criterion at the credible accidental level. The attainable saving is, therefore, 18.4% of the baseline, nearly three quarters of the ceiling: 0.3 points of the remainder are the bar set, 1.7 are the strength criterion at the credible accidental level, and the rest is what the frame withholds.

The second is a design pointer. The frame, which is 74.9% of the mass, was excluded from the parametric space precisely because it is a welded assembly that cannot readily be re-sectioned. The present result converts that practical constraint into a quantitative statement of what it costs: further mass reduction in this structural form has to be sought in the topology and the member layout of the frame itself—for example, by replacing the solid welded rings with a trussed ring or by making the mesh deck participate in bending—rather than in the sections of the columns. Any such change lies outside the scope of the present study and is left as the obvious next step.

### 3.4. Multi-Criteria Evaluation Under Three Weighting Paths

**Figure 7:**
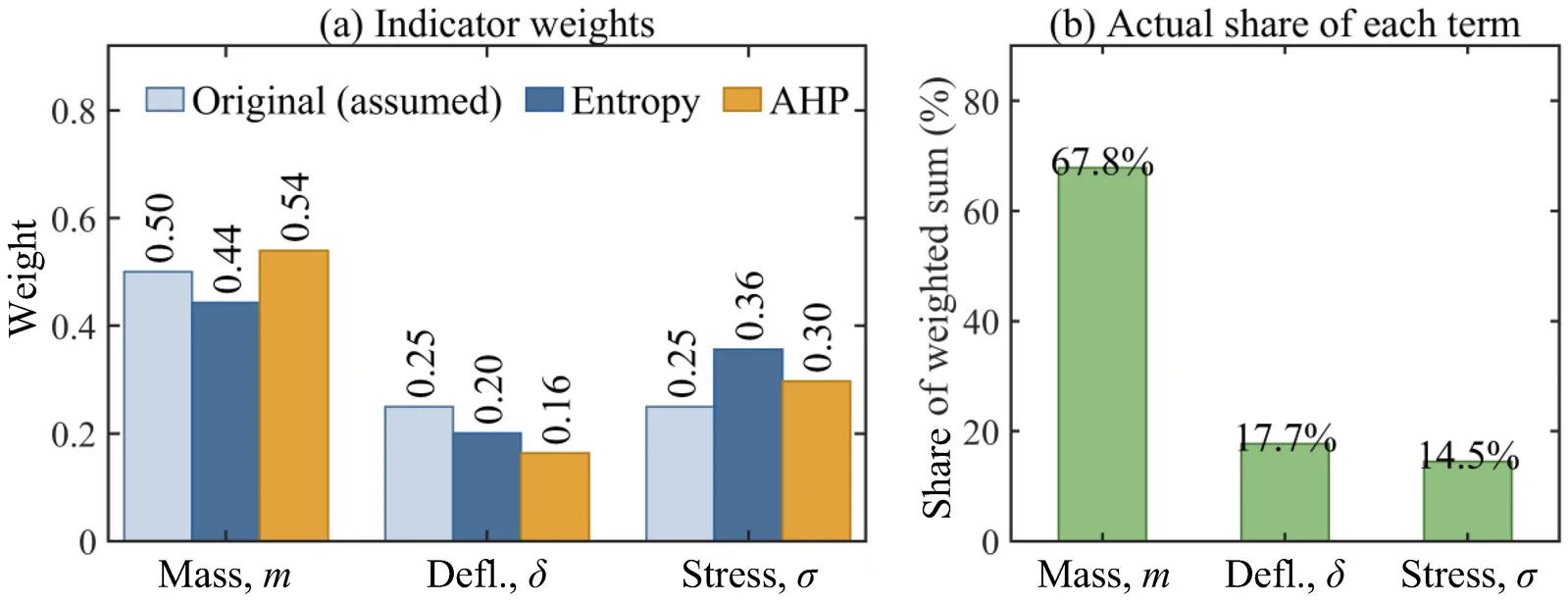
Weighting systems: (**a**) assumed, entropy and AHP weights; (**b**) actual share of each term in the original weighted sum.

The defect of direct weighting, quantified. If the raw values of the three criteria are added directly with nominal weights of 0.5/0.25/0.25, as in the earlier work, the mean contributions of the mass, deformation and stress terms to the weighted sum are 58.66, 15.33 and 12.51 out of a total of 86.50—that is, 67.8%, 17.7% and 14.5%. Because the three criteria carry different dimensions and span two to four orders of magnitude, the nominal weights are not the weights that act: a nominal “half of the decision for mass” behaves as nearly “68% for mass”, while the deformation criterion, nominally weighted at a quarter, is effectively annihilated. The effect is systematic and invisible in the arithmetic of the weighted sum; a comparison of the assumed weights, the two weighting systems and the actual shares are shown in Figure 7.

Constraint screening precedes ranking. The three requirements of Equation (2) are applied first, on the nominal wall. For the design case, the load for Equation (1) lies between *W* = 47.20 and 48.15 kN across the thirty-six solved tube schemes and is 47.08 kN for the recommended scheme, the value quoted in Section 3.10; that scheme is not one of the thirty-six, because it carries the eight 5 mm bars of Equation (16) rather than the 20 mm set the family was solved with, and each scheme contributes three times its own mass to the equation. With the cocooning substrate included, 28 of them pass and the screen removes eight. The worst of the eight is Scheme 4, 159.2 MPa, that is, *SF_y_* = 1.48, a stability safety factor of 1.44 and a deflection utilisation of 26.0%. The same scheme at the deepest operating depth of 60 mm, read on the flat-plan axis in Table 3, results in 20.8 MPa, *SF_y_* = 11.3 and a deflection utilisation of 3.4%; for the water the cone actually holds, it is 18.0 MPa and *SF_y_* = 13.1 on the higher reading of the load scaling and 2.8 MPa and 85 on the other. Read on one basis throughout, the design-case stress is more than eight times the operating value, and every figure in this paper states which level and which reading it is on. This is itself a result, and it reverses the reading of the design space. At the operating depth, the screening requirement separates nothing: every one of the 36 schemes satisfies it with at least a factor of six in hand. At the credible accidental level, it separates eight, all of them at the slender end—the 20 × 20 mm four-tube scheme and seven of the nine three-tube schemes, the two survivors of the three-tube family being the 30 × 30 and 35 × 35 mm ones. Among the survivors, the choice is then settled by the objective and by manufacturability. The weighting analysis below, therefore, has a definite question to answer: whether the preference ranking used in this field lands on a scheme that is also sound on the objective—which it does not. The screen becomes binding at larger operating depths, and Section 3.9 shows that there it determines the answer.

Ranking within the feasible set. The ranking is computed from the relative closeness of Equations (4)–(7) under the three weight vectors of Equations (8) and (9), and it is reported in Table 8. The entropy weights are *w*^E = (0.4433, 0.2004, 0.3563)ᵀ, the AHP weights are *w*^A = (0.5396, 0.1634, 0.2970)ᵀ with *λ_max_* = 3.0092, CI = 0.0046 and CR = 0.0079, and the combined weights at α = 0.5 are *w* = (0.4915, 0.1819, 0.3266)ᵀ. The three paths are reported side by side in Table 8, with gray relational analysis as an independent method. Two observations are worth recording.

First, the entropy method, which knows nothing about engineering intent, orders the criteria exactly as the judgement does: mass first, stress second, and deformation last. The agreement is not a coincidence of small numbers—the mass values span 105 to 138 kg, the stress values 29 to 87 MPa and the deformation values 0.50 to 1.34 mm, and the entropy weight is a monotone function of that dispersion—but it is also not a proof, because dispersion would rank a criterion first even if it were irrelevant to the decision. It is reported as support, not as validation.

Second, Scheme 5 (eight 25 × 25 × 2.5 mm tubes) is first under all three weighting paths, and the leading group is stable: under the combined weights, the top eight are Schemes 5, 9, 1, 6, 13, 14, 10 and 15, and the entropy ranking replaces only Scheme 15 by Scheme 29. Gray relational analysis, however, puts Scheme 9 first and Scheme 5 second. Its leading eight—9, 5, 11, 15, 6, 16, 19 and 1 (Table 8)—share only four schemes with the TOPSIS leading eight, and the reason is the distinguishing coefficient rather than the weights: with ρ = 0.5 the grey relational grade compresses the high-quality end of every criterion, so schemes of middling mass but low stress (11, 15, 19) rise above schemes that TOPSIS rewards for their mass (13, 14, 10). The two methods agree that the leading pair is {5, 9} and disagree about their order and about the tail of the list; since the pair differ by 2.9 kg of steel, the disagreement that has a bearing on the design is of no engineering consequence, and it is reported rather than resolved by selecting the method that provides the desired answer.

### 3.5. Robustness of the Selection

Sweeping the combination coefficient, α, is the weakest of the available sensitivity tests, because it perturbs one weight while leaving the judgement matrix, the data, the criteria and the alternatives untouched. Six perturbations are, therefore, examined; they are collected in Table 9 and discussed in turn.

**(1) Preference uncertainty: enumeration of the AHP judgement.** Each of the three upper-triangular entries was allowed any of the 17 Saaty-scale values, yielding 4913 matrices, of which 1087 (22.1%) satisfy CR ≤ 0.10. Over the admissible set, the top-ranked scheme is Scheme 5 in 643 cases and Scheme 9 in 444, and the rank of Scheme 5 is one or two in every admissible case. That statement depends on the combination coefficient, and the dependence is reported rather than left implicit: entering the same matrices as a pure AHP weight, with the entropy term removed, makes Scheme 9 lead in 548 cases against 295 for Scheme 5, places Scheme 5 outside the top two in 221 cases and as low as rank 18 in 2 of them (Table 9). The combined weight of Equation (9) is, therefore, not a device for averaging two opinions; it is what removes the tail in which preference alone displaces the reference scheme. The 78% of matrices that fail the consistency test are reported so that the reader can see how narrow the admissible region is.

**(2) Data uncertainty: leave-one-scheme-out entropy weights.** Re-estimating the entropy weights 36 times produces *w*_1_ ∈ [0.3082, 0.4609], *w*_2_ ∈ [0.1777, 0.2191] and *w*_3_ ∈ [0.3339, 0.5060], with Scheme 5 leading in 34 runs and Scheme 9 in the other two. The weights are, therefore, not controlled by a single influential alternative, which is not automatic at this sample size; the widest interval, *w*_3_, is ±20.5% about the midpoint of its range, against ±19.9% for *w*_1_ and ±10.4% for *w*_2_. A perturbation of that size is material for a close pair, and the two leaders are not close: they differ by 2.89 kg of mass and 3.47 MPa of stress, far more than the weight spread, so the ranking is not weight-limited.

**(3) Criterion-set uncertainty.** Deleting one criterion at a time produces the leading triples {9, 5, 29} without mass, {5, 9, 1} without deformation and {15, 19, 6} without stress; the rank of Scheme 5 is 2, 1 and 15 respectively. The first two perturbations are benign. The third is not, and the reason is instructive: stress is the criterion on which the small sections score worst, so deleting it automatically favours a larger section. In the present procedure that trade-off is already decided by the screen of Equation (2), which is why a criterion set without stress is inconsistent with the stated requirements; the perturbation is, nevertheless, reported as the weakest point of the robustness of the preference ranking, and it is one reason the recommendation is stated as a minimum-mass choice within the feasible set rather than as the output of the ranking.

**(4) Alternative-set uncertainty.** Removing the four lightest schemes, the four heaviest, the four most highly stressed, or one complete edge-length level (20 or 60 mm, 32 schemes remaining) changes the leader in three of the five cases, always to Scheme 9, and the rank of Scheme 5 is one or two in all five. The impression created by the α sweep—that Scheme 5 leads by a wide margin—is, therefore, not an artefact of the extreme alternatives. The largest single effect comes from removing the four lightest: the mass criterion then loses much of its dispersion, the entropy weight of mass falls, and the ranking shifts towards the heavier but less stressed sections. This is a property of entropy weighting rather than of the design, and it is the quantitative argument for combining it with a judgement weight instead of using it alone.

**Figure 8:**
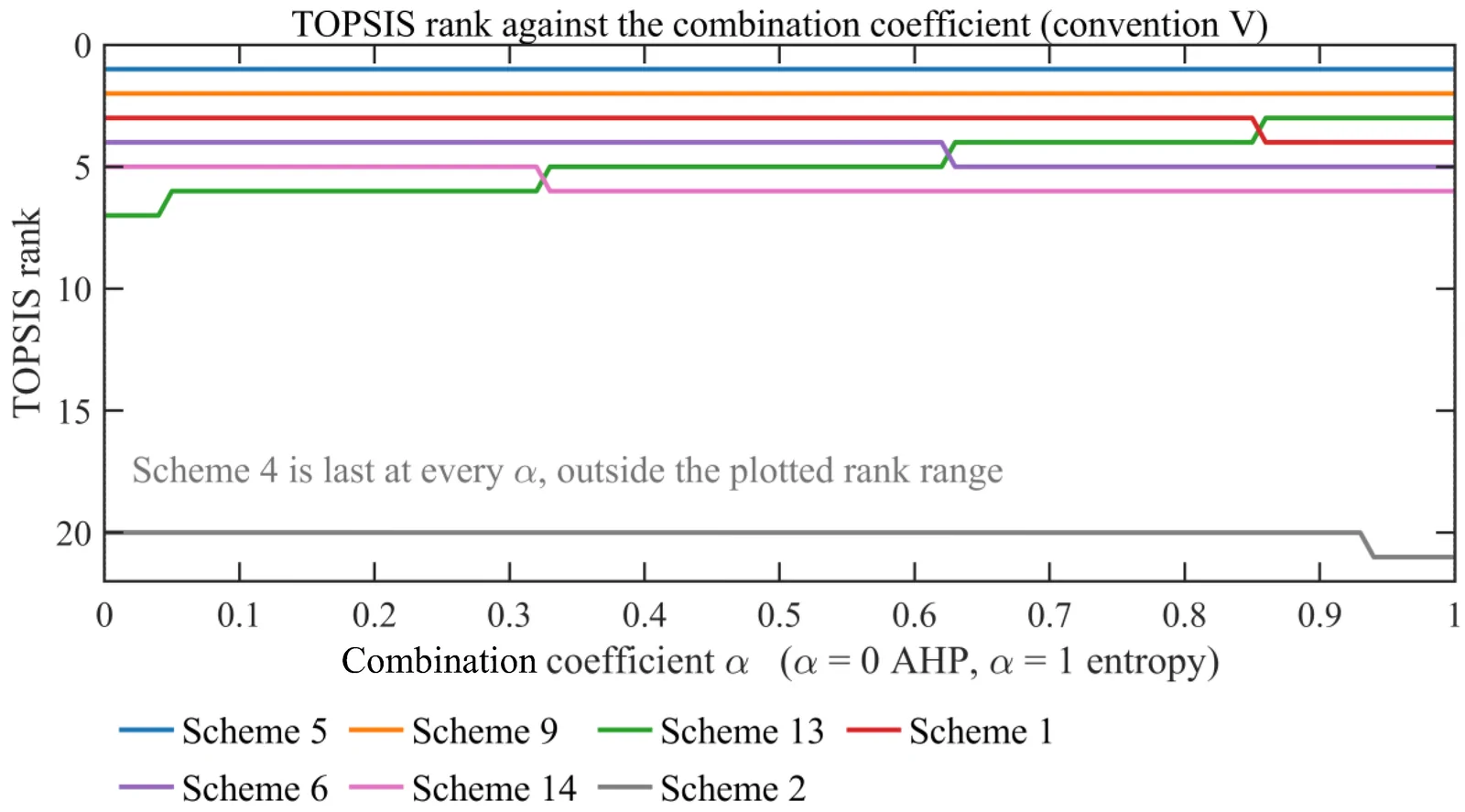
Variation in the TOPSIS ranks with the combination coefficient, α, under convention V (vector normalisation with a cost-type ideal): the mass-minimising Scheme 4 is last at every α and lies outside the plotted rank range. Under convention R, the same sweep moves Scheme 5 to ranks 10–14 and Scheme 4 to 25–32.

**(5) Combination coefficient.** Sweeping α over [0, 1] in 101 steps yields Scheme 5 as the optimum at every step—the two weighting systems agree on the leader over the whole range, which is exactly why this sweep alone is not a sufficient sensitivity analysis (Figure 8).

**Figure 9:**
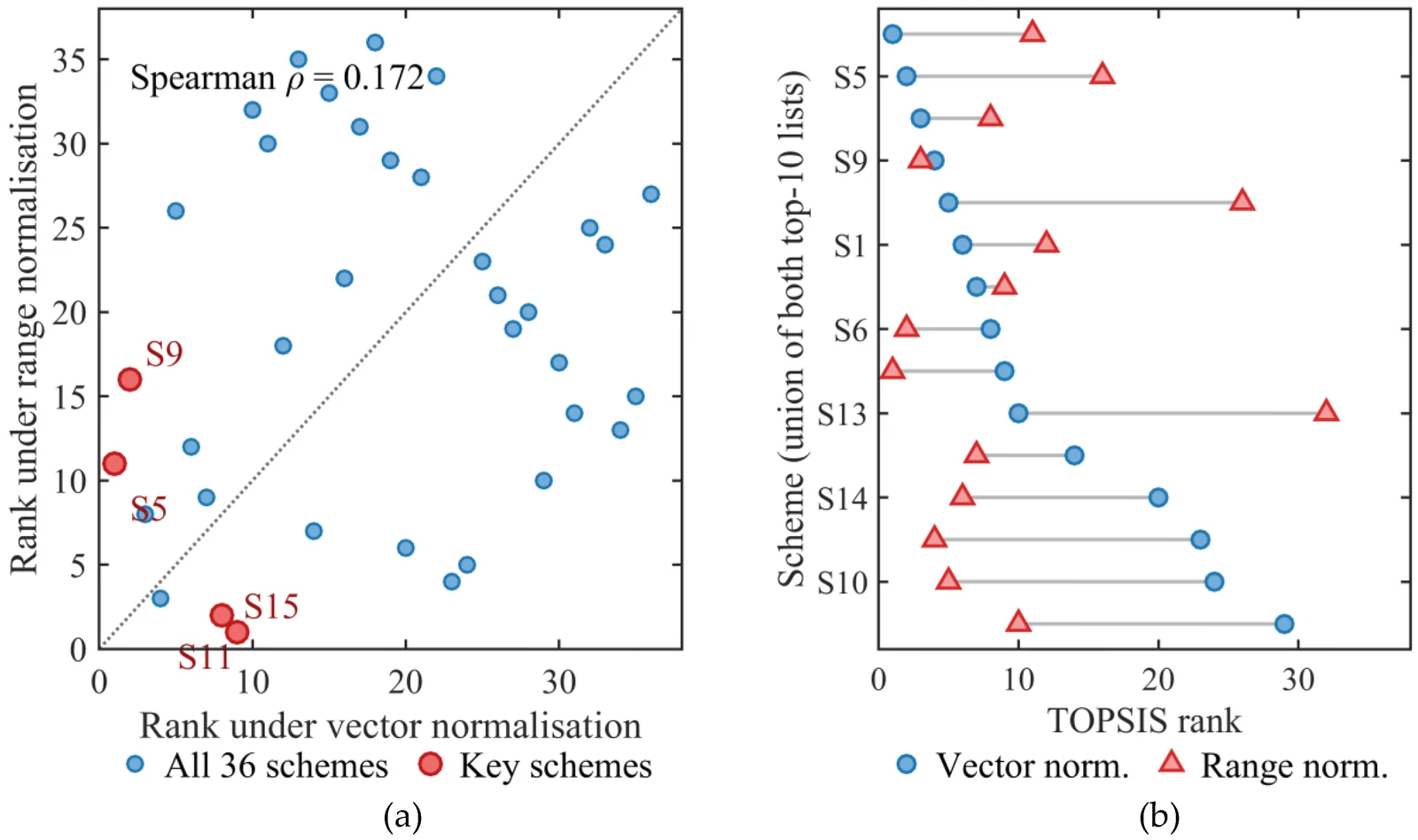
Sensitivity of the TOPSIS ranking to the normalisation convention: (**a**) rank–rank scatter under vector and range normalisation; (**b**) dumbbell chart comparing the two conventions for the union of the two top-10 lists.

**(6) Normalisation convention.** Recomputing the ranking under convention R in Section 2.6(4)—range normalisation with a benefit-type ideal—instead of convention V changes the leading group almost completely: the top five become Schemes 11, 15, 6, 16 and 7 against 5, 9, 1, 6 and 13, the two top-ten lists share only five schemes, Scheme 5 moves from rank 1 to 11, and the Spearman rank correlation is only **ρ = 0.172** (Figure 9, Table A2). This is reported as a negative result: the ranking of a discrete design set by TOPSIS is not convention-invariant, and a study reporting a single convention is reporting a choice it has not disclosed. The cause is identifiable: range normalisation maps the lightest and the heaviest scheme onto 0 and 1, whatever the spacing between them, so the ideal distances are set by the two extremes and the interior is compressed, whereas vector normalisation retains the relative magnitudes and is far less sensitive to a single extreme alternative. Convention V is adopted here because it does not discard magnitude information.

**Consequence for the present study.** The six perturbations show that the **preference** ranking of this design set is robust to the judgement and to the data and **not** robust to the criterion set, the alternative set or the normalisation convention. The decision in Section 3.7 and Section 3.10 is, therefore, not presented as the output of a ranking but as the minimum-mass choice inside the feasibility set, the stated objective in Section 2.6(1), with the ranking used only to establish that the choice is also preference-defensible under the declared convention. Mandatory requirements are not traded against one another, the stated objective decides, and the preference analysis is corroboration with its own uncertainty attached.

### 3.6. Evaluation of the Round-Bar Subsystem

Across the 17 round-bar design points, the mass ranges from 111.32 to 118.04 kg, the maximum total deformation from 50.42 × 10^−2^ to 117.57 × 10^−2^ mm and the maximum equivalent stress from 32.63 to 41.62 MPa. The entropy weights are (0.3222, 0.3339, and 0.3439)ᵀ, that is, almost equal but no longer ordered in the same way as in the tube subsystem: here, the deformation range (67.15 × 10^−2^ mm) is far wider than the mass range (6.72 kg), so the deformation criterion has, by far, the widest spread, although the three weights differ by no more than 0.0217 and the stress criterion is marginally the largest. The same mechanism that made the entropy weights agree with the engineering intent in one design set makes them disagree with it in another, and this is a concrete demonstration of why an entropy weight is a property of the sample and not of the criterion.

The consequence is visible in the outcome: under these weights and under the vector normalisation with a cost-type ideal used for the tube subsystem above, TOPSIS selects Scheme 13 (20 mm × 8); under range normalisation with a benefit-type ideal, the same weights select Scheme 2 (5 mm × 6) instead. The earlier study selected Scheme 1 (5 mm × 8) on empirical weights. The trade-off is direct—Scheme 13 results in 118.04 kg, 0.5042 mm and 32.63 MPa, whereas Scheme 1 yields 111.75 kg, 0.5497 mm and 34.43 MPa—so the entropy method is buying a 5.2% reduction in stress, with a 5.6% increase in mass, precisely the opposite of the declared objective. A data-driven weighting is not automatically the right weighting, and a cost-dominated engineering objective can be overturned by it. This is the empirical justification for the combined weighting adopted here, and it is the second piece of evidence that the decision must be stated in terms of the objective rather than read off a ranking.

Mechanically, the subsystem is a weak lever. In the mass–stress plane, the non-dominated schemes are 2, 3, 4, 6 and 13, together with the control configuration 17 (no bars); Scheme 1 is not on that two-dimensional front, although it is non-dominated once deformation is included as a third objective. The reason is that the bar diameter is saturated: across the 17 schemes, going from a 5 mm to a 20 mm bar—a sixteen-fold increase in area—reduces the maximum total deformation from 54.97 × 10^−2^ to 50.42 × 10^−2^ mm, that is, by only 8.3%, whereas reducing the bar **number** from eight to two raises it from 54.97 × 10^−2^ to 80.04 × 10^−2^ mm, by 45.6%. The bars, therefore, act through the number of restraints they provide and not through their own bending stiffness, which is negligible at this diameter. This is the same conclusion as in the tube subsystem, where the number of load paths rather than the section governed the stress, and it is the mechanism behind the connection effect analysed in Section 3.10(2).

One methodological gap of the earlier work is worth restating here. After fixing the square tubes at 25 × 25 mm and *n* = 8, the round bars were optimised **on their own**, so the two subsystems were never evaluated jointly, and the interaction between them was never examined. Section 3.7 quantifies what that cost.

### 3.7. Overall Selection and the Pareto Front

**Figure 10:**
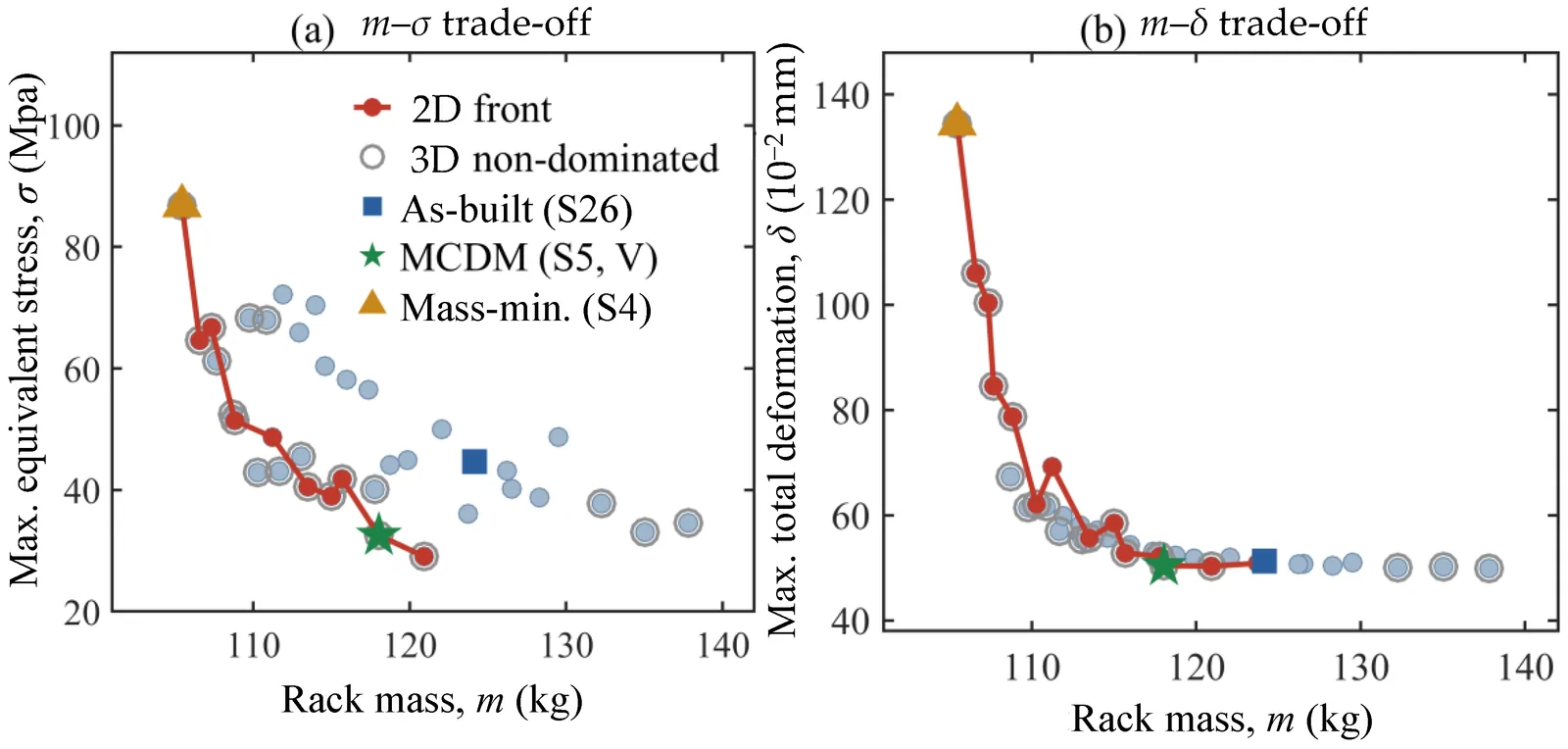
Pareto fronts: (**a**) mass–stress; (**b**) mass–deformation. The solid line in each panel is that panel’s two-objective front; hollow circles mark the schemes that are non-dominated when mass, deformation and stress are considered together. Scheme 5 is the TOPSIS optimum under convention V and ranks 11th under convention R; Scheme 4 is the mass minimum.

Ranking the 36 tube schemes under the combined weights and extracting the three-objective non-dominated set (*m*, *δ* and *σ* all minimised) yields 20 non-dominated schemes; both Scheme 5 and Scheme 4 lie on the front, whose projections onto the mass–stress and mass–deformation planes are shown in Figure 10. The optima obtained under different decision rules are collected in Table 10.

Two things are apparent from Table 10. First, the preference-based rules and the mass-based rule disagree in a way that has nothing to do with the weighting: the ranking rules all place an eight-tube configuration first, whereas minimum mass selects a three-tube one. The ranking is not indifferent to mass—the entropy weight of the mass is the largest of the three—but it is a compromise, and a compromise cannot return the mass optimum of a set in which mass and stress trade off monotonically. If the objective is the minimum mass, the decision must be stated as the minimum mass, with the ranking reported as a corroborating analysis; this is the principal correction the present study makes to the earlier work and why the objective is stated explicitly in Section 2.6(1).

The second is that mass minimisation alone is not a complete instruction either, because the lightest scheme is also the least stiff and the most stressed. This is where the mandatory constraints do their work. Applied to all 36 joint configurations of the design case with the substrate included and the strength checks on the 235 MPa basis in Section 2.1, the screen of Equation (2) admits 101.34 kg as the lightest. The recommended scheme is, therefore, the lightest joint configuration in the entire design space for which every criterion is satisfied at the credible accidental level—18.4% below the baseline and 9.3% below the earlier final design—while the absolute minimum, 99.21 kg, is retained as a *predicted candidate*, because in the design case, it fails both criteria (1.48 and 1.44) and, unlike the recommendation, its stress rests on an extrapolated connection factor. Both masses belong to joint tube-plus-bar configurations and are not members of the 36 finite element schemes in Table A1.

The mass reduction potential of the structural form is summarised in Table 11.

Two conclusions follow. The 9.98% achieved in the earlier work is just over half of the 18.4% the same subsystem now delivers, for two identified reasons: the evaluation criterion was a compromise that could not return the mass optimum of the set, and the two subsystems were optimised sequentially rather than jointly. In addition, the remaining 74.9% of the mass lies in the frame, which no section-level optimisation can reach, so the route to a substantially lighter rack runs through the topology of the mesh frame and not through the size of its columns.

### 3.8. Stability, Serviceability and Corrosion-Allowance Checks

**Figure 11:**
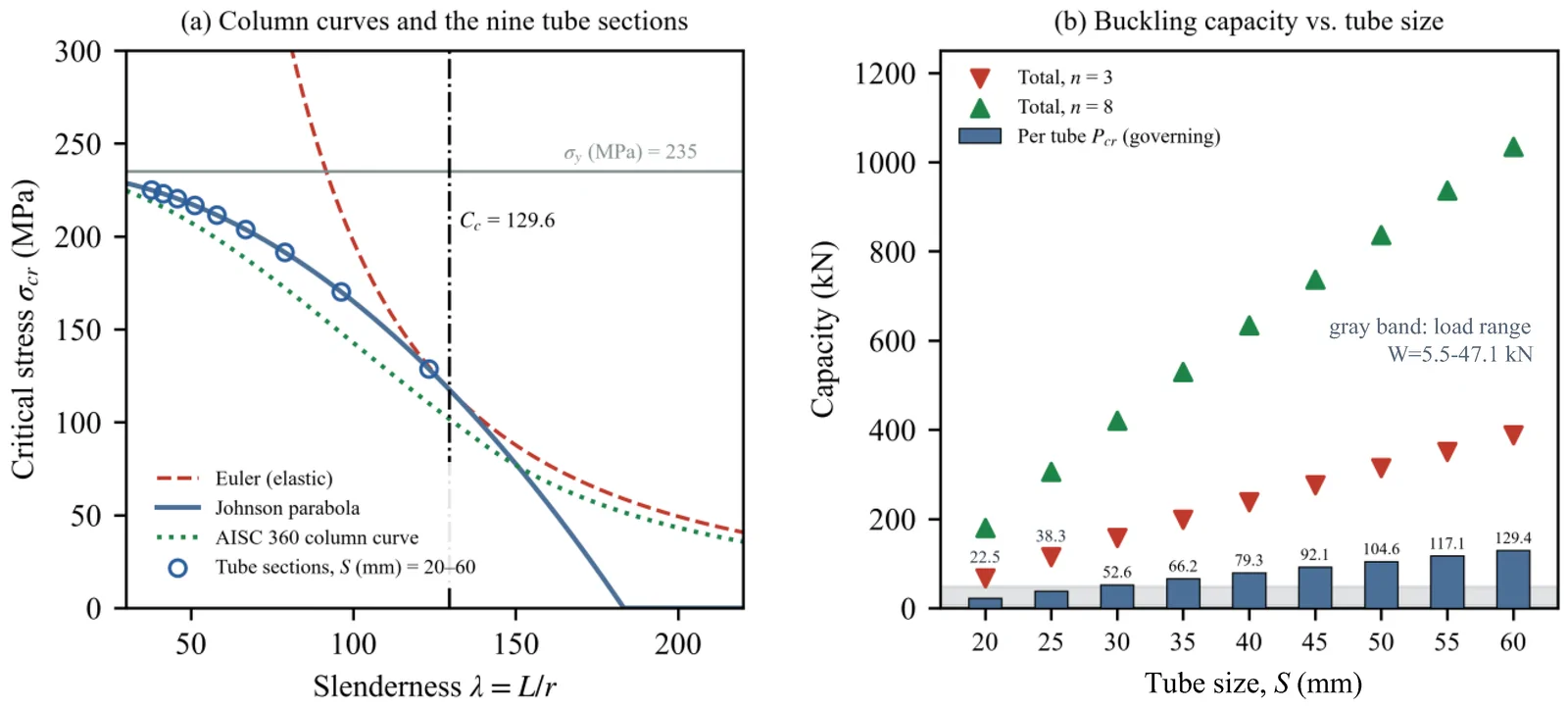
Buckling checks on the 235 MPa basis in Section 2.1: (**a**) Euler, Johnson and AISC 360 column curves with the nine tube sections, whose slendernesses all fall below the elastic–inelastic transition; (**b**) per-tube buckling capacity (bars) and total capacity for three and eight tubes (markers) against tube size; the gray band spans the load range considered here, from the operating range (5.5–6.1 kN) to the design case (47.1 kN).

**(1) Buckling regime and critical capacity.** The section properties of the nine tube sizes computed from Equation (12), and the critical stresses from Equation (13), are given in Table 12; the three column curves and the positions of the nine sections are shown in Figure 11.

The methodological conclusion is unambiguous: All nine sections are intermediate columns. Their slenderness ratios run from 37.9 to 123.3, below *C_c_* = 129.61, so none may be checked with the Euler formula. Euler would overstate the critical stress of the 25 × 25 mm section by 25.1% (212.85 against 170.14 MPa) and its critical load from 38.28 to 47.89 kN, an overstatement no subsequent safety factor recovers. The AISC 360 curve returns 148.04 MPa for the same section, lower still because it embodies residual stress and initial crookedness, and is retained as a lower design bound. The 20 × 20 mm section sits closest to the transition—its slenderness is 95.1% of *C_c_*—and is the only one for which the grade of steel still matters at all (Section 4.3).

Two assumptions carry a quantified sensitivity. The effective length factor: the members are welded to a stiff annular ring above and to the base ring over the 890 mm height, so *K* = 1.0, the pinned–pinned value, is a lower bound on the restraint actually available; *P_cr_* of the 20 × 20 mm section rises from 22.51 kN at *K* = 1.0 to 27.68 kN at 0.85 and 32.00 kN at 0.70, so up to 42% of the stability capacity of the slenderest section is withheld deliberately, and a measured rotational stiffness is the single most valuable addition to this work. The recommendation is far less affected—52.64 to 58.75 kN for the 30 × 30 mm section, 11.6%—because it is the stockier member. The axial-force distribution: the load is taken as shared equally, *W*/*n*; at 1.2 times the mean, the stability safety factor of three 20 × 20 mm tubes falls from 1.44 to 1.20 and that of the recommended three 30 × 30 mm tubes from 3.35 to 2.80, the latter still above the criterion and the former already below it. The recommendation is, therefore, unchanged by the bound and the lightest section is not, which is why equal sharing is accepted with its bound stated.

**(2) The governing limit state and the ratio that decides it.** For any scheme, the ratio of the two safety factors defined in Equation (2) is

(17) $$\frac{SF_{y}}{SF_{b}}=\frac{\sigma_{y}W_{d}}{\sigma_{d}\Sigma P_{cr}}$$

where *σ_d_* is the peak stress produced by the load, *W_d_*. At a fixed load case, this is an identity, and it is what decides which criterion governs: the ratio is below unity wherever strength is the tighter of the two. It is not independent of the depth at which it is evaluated, because *W_d_* carries the pans, substrate and rack in full at every depth, whereas *σ_d_*, on the scaling in Section 2.3, is the water term alone: the dead load is half of W_d_ at 60 mm on the flat-plan axis in Section 2.3 and 11% of it in the design case, and for the slender three-tube schemes the ratio falls from 1.10 to 0.62 between the two—stability governs at the operating depth and strength at the accident, which follows from the basis and not from the criteria. On a consistent basis, with the dead load carried at its own mass in both quantities, the same ratio is 0.69 and 0.67, 3% apart. The identity is, therefore, used to order schemes at one load case, and every statement below about the governing criterion is made in the design case, where the screen of Equation (2) is applied. Across the 36 schemes in Table A1, it lies between 0.170 and 1.027 there—below one for 34 of them and just above it for the two 20 × 20 mm schemes with the fewest tubes (1.002 and 1.027)—so strength governs the design space, except at its slenderest corner, where stability takes over, which is the corner where the chart in Section 3.9 breaks first. The conclusion is robust to the one quantity in it that is not converged: local refinement at the stress concentration can only raise the peak stress, which lowers *SF_y_* and with it the ratio of Equation (17), so the discretisation error works in the direction that strengthens this conclusion rather than the one that would overturn it.

The practical value of the ratio is that it compares the two criteria on one load case rather than on two margins that respond differently to depth. It also locates the mass that can be removed: reducing from eight 25 × 25 mm to three 20 × 20 mm tubes retains 22.1% of the buckling capacity (306.2 to 67.5 kN) but 37.6% of the strength margin (3.94 to 1.48), so stability capacity is the more fragile quantity under design reduction—which is why the corrosion allowance, the attacking capacity rather than the load, is expressed as a level in Section 3.10(5).

**Figure 12:**
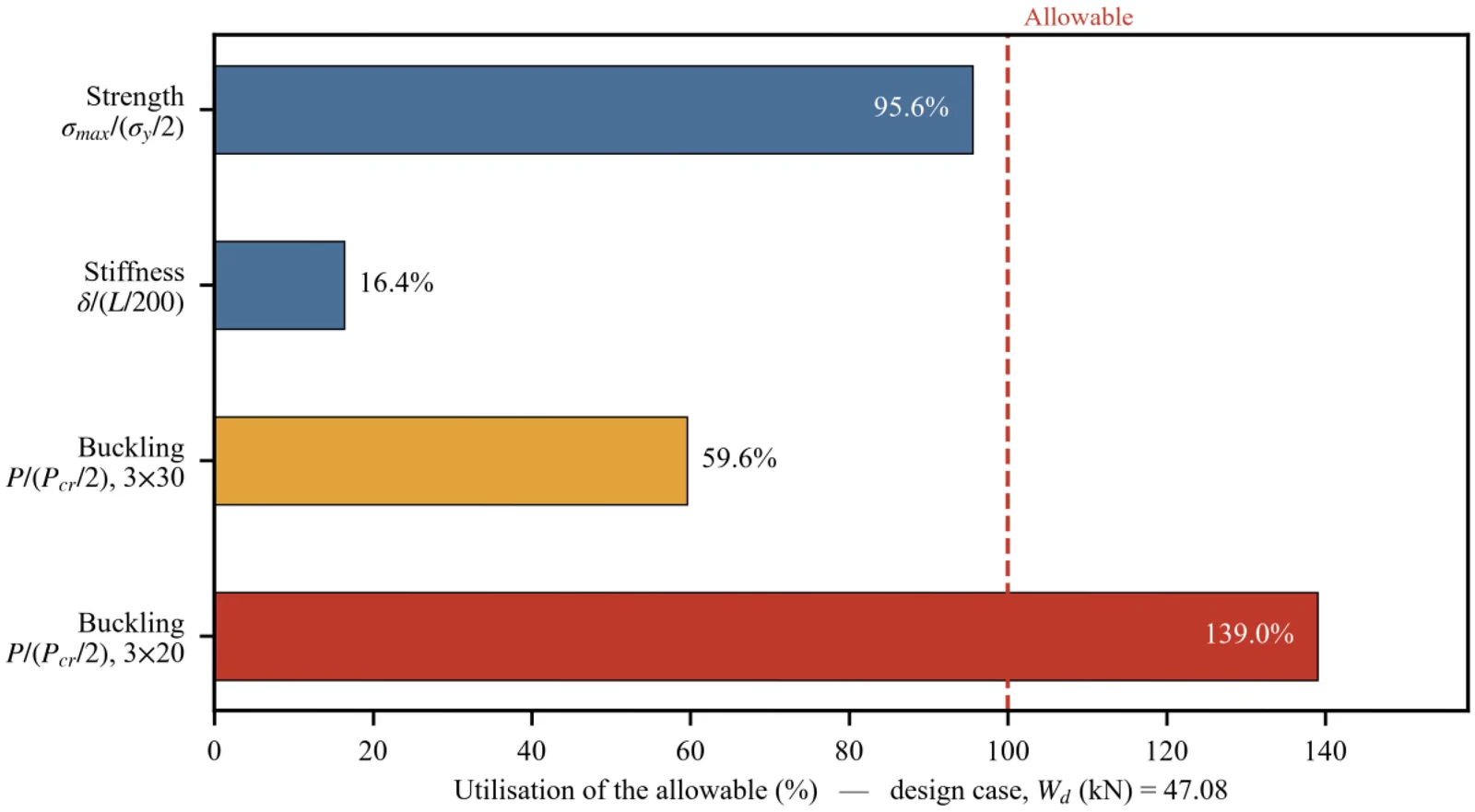
Utilisation of each criterion in the design case in Section 2.3, expressed against the allowable of that criterion on the 235 MPa nominal minimum: The peak stress of the recommended scheme against *σ_y_*/2, its deflection against *L*/200, and the per-column buckling load of the recommended three 30 × 30 mm tubes and of the lighter three 20 × 20 mm ones against *P_cr_*/2. A bar is, therefore, admissible below 100%, which is the dashed line; the candidate’s buckling bar crosses it at 139%.

**(3) Strength and stiffness utilisation and the meaning of a sub-millimetre deflection.** After optimisation the recommended scheme reaches a maximum equivalent stress of 112.3 MPa in the design case (Section 3.10), a yield safety factor of 2.09 and a material utilisation of 47.8%; against an allowable stress of *σ_y_*/2 = 117.5 MPa, the reserve is 4.6%. Its maximum total deformation is 1.603 mm, 16.4% of the *L*/200 = 9.80 mm limit and 32.7% of the stricter *L*/400 limit. The utilisation ratios are compared in Figure 12.

The governing quantity is the deflection relative to the water level, not the deflection itself. What must be uniform in a tiered vessel is the water depth across a pan and between tiers, which is what Section 2.2 requires and the welfare argument in Section 1.3 rests on. The most flexible scheme, the predicted candidate, deflects 0.33 mm at the deepest operating depth and 2.55 mm in the design case—0.55% of the water depth at both, because the deflection in this scaling is carried by the water alone and the depth measures the same water. The larger is a quarter of the *L*/200 limit and the smaller a thirtieth, so the water film stays uniform to within 1% of its depth on any scheme in the design space, and the *L*/200 limit is retained as a conventional screen and does not govern.

**(4) Corrosion allowance, and its effect on all three limit states.** Section 2.8(4) sets out the exposure classification, the 10-year design life, the unprotected loss rate of 0.05–0.15 mm·a^−1^ and the reason the allowance is not optional; those assumptions are not repeated here, and what is reported is their structural consequence. Hot-dip galvanising is treated as mandatory.

What the coating life does and does not establish. A 65–85 μm coating losing zinc at 3–10 μm·a^−1^ protects for 6.5–28 years, so the range is a set of conditions and not a property, and the 10-year design life sits inside it but not inside its lower part. The statement is conditional, and the conditions are stated as maintenance requirements in Section 4.5(7) rather than as assumptions: at least 65 μm on every member; annual inspection of the splash zone at the pan rim and of the weld toes; repair of any breach at the inspection that finds it; and re-coating before year 10 if the coupon rate of step (v) exceeds 6.5 μm·a^−1^. Without them, the unprotected rate of 0.05–0.15 mm·a^−1^ returns at the first breach, and the design life is not supported.

Strength, stability and stiffness on the corroded wall. Reducing the wall to t_eff_ = 1.75 mm, a one-sided loss of 0.75 mm, and recomputing the section properties from Equation (12) produces a reduction that is section-dependent but identical for the two quantities that matter: for 25 × 25 × 2.5 mm, the second moment of area and the section modulus retain 76.7% (critical load of 73.9% and bending stress up 30.3%); for 20 × 20 × 2.5 mm, 78.5% (77.2%, up 27.4%); and for 30 × 30 × 2.5 mm, 75.6% (72.7%, up 32.4%). Applied to the recommended scheme, the corroded wall raises its design-case stress from 112.3 to 148.7 MPa—SF_y_ from 2.09 to 1.58—and its deflection from 1.603 to 2.121 mm, 21.6% of the L/200 limit. The accidental level is an accidental action and the allowance a service-life of one, so the two are not normally combined; the consequence of combining them is that at the end of life, the rack covers a water level of 364 mm rather than 460 mm, which Section 3.10(5) states as the end-of-life admissible level. The stability margin does not survive it in the design case (3.35 → 2.44), while the stiffness does (16.4% → 21.6% of the allowance), which is why Section 3.10(5) states the allowance as a reduction in the admissible level rather than as a reduction in the section. Stated as a level, the effect is section specific and, therefore, worth quoting on the lightest one: the level at which three 20 × 20 mm tubes are broken by stability falls from 315 mm on the nominal wall to 230 mm on the corroded wall, a loss of 27.0%, whereas the level at which the recommended section is broken by strength falls from 481 to 364 mm. That ordering is what makes the interplay of the two limit states usable: the slender section is the one the allowance removes first, and it is removed by stability, which is the criterion the allowance attacks hardest.

The galvanising mass, member group by member group. The zinc fraction is not one number. For a tube wall coated on both faces, it is the plate fraction of Equation (14), 5.82% at *t* = 2.5 mm and independent of the section; for a solid bar coated on its cylindrical surface, it is the bar fraction of the same equation, 5.82% at *d* = 5 mm, 2.91% at 10 mm, 1.94% at 15 mm and 1.46% at 20 mm. Table A4 resolves the two reference designs on that basis: the coating adds 6.93 kg to the 124.14 kg baseline (5.58%) and 5.90 kg to the 101.34 kg recommendation (5.82%), so the delivered masses are 131.07 and 107.24 kg and the reduction is 18.2% coated against 18.4% bare. The masses quoted in this paper are bare-steel masses, and applying the plate value to the 20 mm bars would overstate the baseline coating by 0.30 kg. The coating is excluded from the load-bearing section, which is why the corrosion check is made on the reduced wall.

### 3.9. Admissible Water Level and Why the Operating Depth Does Not Select the Design

Section 1.3 identified the operating depth as the process variable that husbandry fixes and the structure cannot choose, and Section 2.3 made the chain quantitative. Evaluating it at the corrected load model changes the conclusion in a way that is worth stating plainly: over the declared operating range, the water is so shallow that no section in the design space is close to a limit. At 60 mm, the deepest operating depth, the three pans hold 73.5 kg of water and the recommended scheme carries 6.09 kN. On the reading in which the dead load is carried at its own mass—the higher of the two at this level, because the extrapolation from the archived solution runs downward—that load reaches 12.7 MPa and 0.18 mm, standing at *SF_y_* = 18.5, *SF_b_* = 25.9 and 1.8% of the deflection allowance; scaling the whole solution by the water ratio, which is the reading tabulated in Section 3.10(3), yields 1.9 MPa and *SF_y_* = 121. The lightest standard section, three 20 × 20 × 2.5 mm tubes, is at 18.0 MPa and *SF_y_* = 13.1 on the higher reading. The flat-plan axis in Table 3 carries 7.5 times that water at the same nominal level and is the heavier of the two readings—10.8 kN, 14.6 MPa and *SF_y_* = 16.1 for the recommended scheme against 11.3 for the lightest, which is the operating column in Section 3.10(3)—and even that reading is still more than eight times the criterion. A rack sized by its operating depth would, therefore, be sized by nothing.

**Figure 13:**
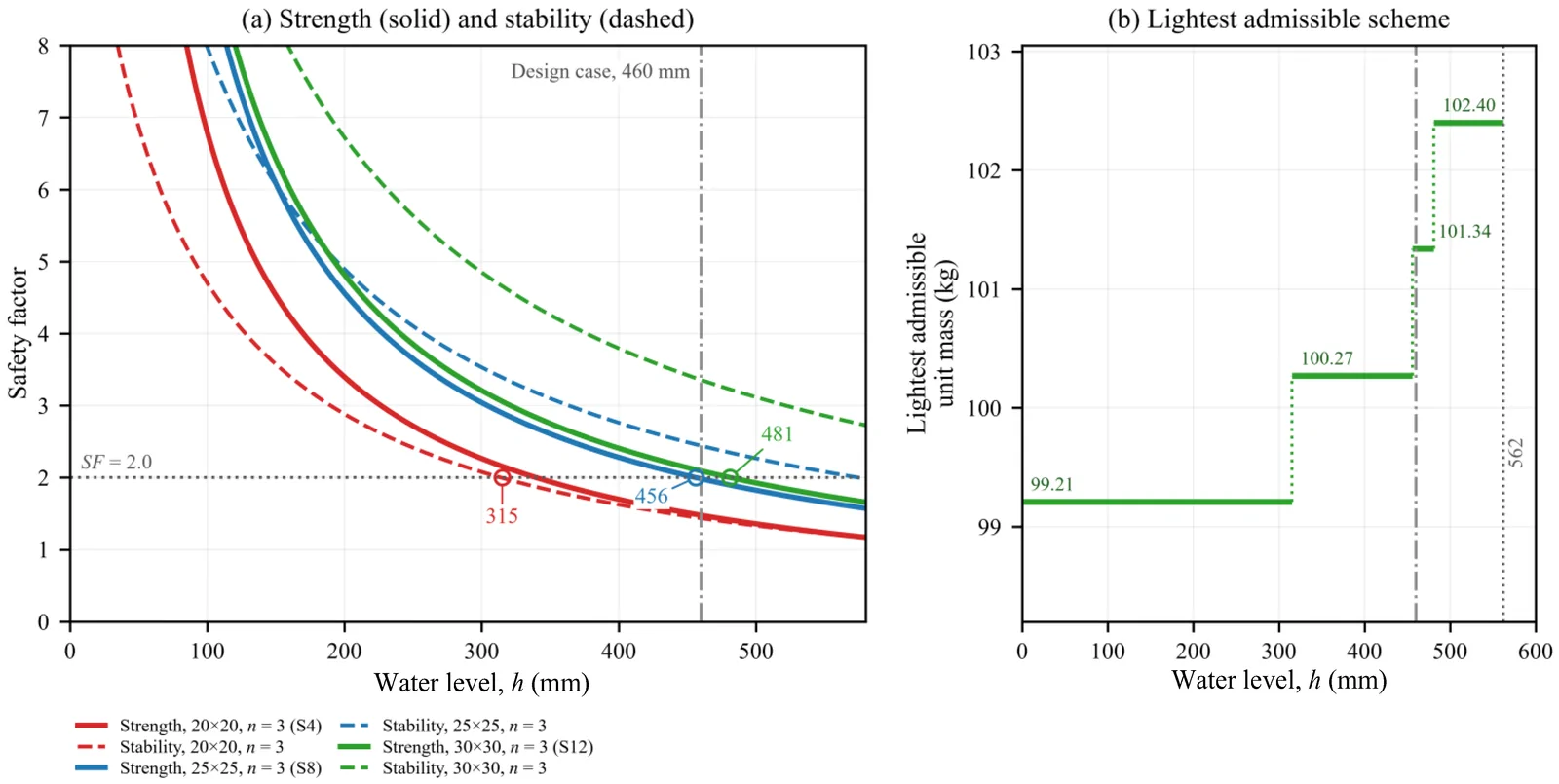
Admissible water level: (**a**) strength (solid) and stability (dashed) safety factors of the three three-tube schemes against the water level, with the SF = 2.0 criterion, the three levels at which a scheme is broken (315 mm for three 20 × 20 × 2.5 mm tubes, by stability; 456 mm for three 25 × 25 × 2.5 mm tubes and 481 mm for three 30 × 30 × 2.5 mm tubes, both by strength) and the design case of 460 mm; (**b**) mass of the lightest admissible standard-section scheme at each level, over the range in which the three-tube family supplies it. Every curve is computed from Equation (1), with the three pans and the envelope capacity in Section 2.3, 1417.3 L, on the 235 MPa basis in Section 2.1.

What selects the design is the credible accidental level in Section 2.3—the pan standing full to its rim—and a useful way to present that is not as one load case but as the admissible water level of each scheme: the highest level on that same flat-plan axis at which it still satisfies *SF_y_* ≥ 2.0 and *SF_b_* ≥ 2.0. That quantity is monotone in mass, is what an operator can measure (a level, not a safety factor), and reduces to the design case by reading it at 460 mm. Table 13 and Figure 13 present it, and the step structure is simple enough to state in four sentences:

Up to 315 mm, the lightest standard section is admissible. Three 20 × 20 × 2.5 mm tubes with the 5 mm bar set (99.21 kg) reach *SF_y_* = 2.27 and *SF_b_* = 2.08 at 300 mm; the criterion that binds is stability, not strength, and it breaks the section at 315 mm.
From 315 to 456 mm, the admissible scheme is three 25 × 25 × 2.5 mm tubes (100.27 kg), which hold 315 mm at *SF_y_* = 2.90 and *SF_b_* = 3.39 and are broken by strength at 456 mm.
From 456 to 481 mm, it is three 30 × 30 × 2.5 mm tubes (101.34 kg)—the recommended scheme in Section 3.10—which stand in the design case of 460 mm at *SF_y_* = 2.09 and *SF_b_* = 3.35 and are broken by strength at 481 mm.
From 481 to 562 mm, it is three 35 × 35 × 2.5 mm tubes (102.40 kg), and beyond 562 mm the three-tube family is exhausted: the answer moves to four and eight tubes, the highest level any scheme in the family reaches being 1015 mm (eight 30 × 30 mm tubes).

**Table 13:** Admissible water level: The lightest standard-section scheme meeting *SF_y_* ≥ 2.0 and *SF_b_* ≥ 2.0 at each level, on the nominal wall and on the 235 MPa nominal minimum yield strength in Section 2.1. The load follows from Equation (1) with the three pans, the 41.6 kg substrate allowance and three units of the scheme’s own mass; the masses are those of the joint configuration, that is of the tube scheme carrying the eight 5 mm bars of Equation (16), and are, therefore, 6.25 kg below the tabulated mass of the 20 × 20 mm row and 6.34 kg below that of the 30 × 30 mm one in Table A1. The stress and deformation of each scheme are its finite element values scaled by the water-mass ratio in Section 2.3.

Flat-Plan Level h = V/A_in_ (mm, Not Depth)	*W*/kN	Scheme	Tubes	Mass/kg	*SF_y_*	*SF_b_*	*SF_b_*,cor	Feasible (/36)
20	7.11	4	20 × 20, *n* = 3	99.21	33.98	9.49	7.33	36
60 (flat-plan level, not a depth)	10.74	4	20 × 20, *n* = 3	99.21	11.33	6.29	4.86	36
150	18.89	4	20 × 20, *n* = 3	99.21	4.53	3.57	2.76	36
200	23.43	4	20 × 20, *n* = 3	99.21	3.40	2.88	2.23	36
250	27.96	4	20 × 20, *n* = 3	99.21	2.72	2.42	1.87	36
300	32.49	4	20 × 20, *n* = 3	99.21	2.27	2.08	1.61	36
340	36.14	8	25 × 25, *n* = 3	100.27	2.68	3.18	2.35	35
400	41.58	8	25 × 25, *n* = 3	100.27	2.28	2.76	2.04	35
452 (wetted capacity, 1391.5 L)	46.29	8	25 × 25, *n* = 3	100.27	2.02	2.48	1.83	29
456	46.65	8	25 × 25, *n* = 3	100.27	2.00	2.46	1.82	29
460 (design case, envelope 1417.3 L)	47.08	12	30 × 30, *n* = 3	101.34	2.09	3.35	2.44	28
481	48.95	12	30 × 30, *n* = 3	101.34	2.00	3.23	2.35	28
545 (flat-plan level at the rim)	54.78	16	35 × 35, *n* = 3	102.40	2.06	3.63	2.61	24

Note: The first column is not a water depth but the flat-plan level of Equation (1), *h* = *V*/*A_in_*, so the conical bottom makes a stated level larger than the depth holding the same water: the deepest operating depth, 60 mm, holds 24.5 L, which is 8.0 mm here (6.09 kN, Section 3.9), and the row labelled 60 is the level that happens to carry that number, holding 7.5 times the water present at that depth (Section 2.3, Table 3). The 545 mm row is admissible only through the 35 × 35 mm section, and the load there is 16% above the measured envelope of the pan, which is why the design case is that envelope rather than the rim of the flat-plan model. The 452 mm row is the same accident read on the wetted capacity in Section 2.3, 1391.5 L, and is given because it is the level at which the 25 × 25 mm section crosses the criterion; the design case is the envelope, 460 mm, for the reason Section 2.3 provides. The *SF_b_*,cor column applies the corroded-wall critical load in Section 3.8(4)—77.2%, 73.9%, 72.7% and 72.1% of the nominal value for the 20 × 20, 25 × 25, 30 × 30 and 35 × 35 mm sections—and is reported for the end-of-life level in Section 3.10(5); it is not a screening column, and the *Feasible* count is on the nominal wall. The substrate allowance is included throughout at 41.6 kg.

The design case of 460 mm falls in the third band, 2.13 kg above the section that would suffice at 300 mm and 21 mm below the level at which the recommended section is broken. That is, the whole of the mass penalty the accidental case costs: 2.1% of the rack mass buys 166 mm of the admissible level—the 481 mm break level against the 315 mm one—while the design case itself sits 145 mm above the level the lighter section would have carried. The distance to the lower edge of the band is where the capacity definition in Section 2.3 enters, and it is small. On the envelope the design case is 4 mm above the 456 mm at which three 25 × 25 mm tubes are broken, so those tubes stand at *SF_y_* = 1.98, and the screen removes them; on the wetted capacity, the same case is 4 mm below that boundary, and the same tubes stand at 2.02 and are admitted. The two definitions straddle the boundary, so the 30 × 30 mm section is chosen to clear the criterion on the heavier of the two defensible capacity definitions, not on the lighter one; Section 3.10(4) carries both sets of numbers and prices the difference, and it also shows that the same section clears the criterion on the conservative reading of the load scaling.

### 3.10. Limit-State Checks of the Recommended Scheme in the Design Case

**(1) The two configurations and the design case.** The **recommended scheme** is three 30 × 30 × 2.5 mm square tubes with eight 5 mm round bars, 101.34 kg per unit—the lightest joint configuration that satisfies every criterion in the design case on the 235 MPa basis in Section 2.1. The predicted candidate is three 20 × 20 × 2.5 mm tubes with eight 5 mm bars, 99.21 kg, the lightest joint configuration of all, and the three 25 × 25 × 2.5 mm configuration that the 250 MPa reference recommended, 100.27 kg, is carried in Table 10 and Section 3.9 as the scheme that basis would have admitted. The design case is all three pans filled to their rims, 3 × 1417.3 L, with the substrate and three units of each scheme’s own mass: *W_d_* = 47.08 kN for the recommended scheme and 47.01 kN for the candidate. There is no separate overload case: a 1.5× factor on an accidental level would stack a second accident on the first, and the design case is already the credible maximum.

**(2) How the stress and deformation of each scheme are obtained. In both configurations, the tube frame is directly solved and the assembled rack is not.** For the recommended scheme, the tube geometry is Scheme 12 in Table A1 (*S* = 30 mm, *n* = 3), solved with the pan at 300 mm; its stress and its deflection are scaled to the design case by the water-mass ratio of 1.739 in Section 2.3 and by the two connection factors that the bars introduce, measured on the solved pairs, *k_σ_* = 1.055 for the stress and *k_δ_* = 1.090 for the deflection, so that neither quantity is quoted on the as-solved 20 mm bar set. Both are ratios of the two rows in Table A3—*k_σ_* = 34.43/32.63 = 1.055 and *k_δ_* = 54.97/50.42 = 1.090, Schemes 1 and 13, which differ only in the bar set, 5 mm against 20 mm, eight bars in both, at one tube scheme and one load. The candidate takes the same route from Scheme 4. Neither bar configuration is itself in the 53 solved models, and the paper says so rather than implying otherwise: the tubes are solved, the bars are attached through a factor calibrated on the solved pairs, and the sensitivity of the recommendation to that factor is quantified in Section 4.5(3). An independently coded beam model of the same rack, in which the bars are represented as beam elements rather than as the restraints they are, returns 1.003 for the same factor—which is the demonstration that the effect is not a bending-stiffness effect of the bars at all, and the reason the factor is measured on the solved family instead.

**(3) The design case.** Table 14 presents both configurations in the design case and at the deepest operating depth for reference.

**(4) What the design case decides and why the margin and not the section is the open item.** At 460 mm, the recommendation passes on the nominal wall with *SF_y_* = 2.09 and *SF_b_* = 3.35, and the candidate does not pass either criterion—1.48 and 1.44. The separation between the two configurations is decided by the design case itself rather than by an overload applied to it, and it is wider than the 2.1% mass difference suggests: 2.13 kg of steel is the difference between a scheme admissible at the rim of the pan and one that is not. The extra factor a two-level format would need is not required to separate them.

No single one of the two carries this choice, and that is what makes it a choice rather than a coincidence. Two judgements, each independent and each defensible in either direction, move the 25 × 25 mm section across the criterion: the capacity definition, on which it stands at 2.02 (wetted) and 1.98 (envelope), and the load scaling, on which it stands at 2.14 (dead load at its own mass) and 1.98 (the upper-bound stress in Section 2.3). It clears 2.0 only where both are read in its favour—2.02 and 2.14—and falls below on the conservative reading of either. A third reading is available and is not offered: on the 250 MPa of the earlier design the same tubes stand at 2.11, but that basis is 6.4% above the minimum Q235 is specified to deliver and is used for no check here (Section 2.1); a paper that quoted 250 MPa here and 235 MPa there would be choosing its section by choosing its basis. The 30 × 30 mm section clears the criterion on the conservative reading of both judgements at once, at 2.09 and at 2.26 on the scaling alone. The section is, therefore, the lightest one that is admissible on the conservative reading of both load-side judgements, not the one that wins a comparison by 0.9%, and the price of that robustness is 1.07 kg, 1.1% of the rack mass.

The capacity definition and what it does and does not decide. On the wetted capacity, 1391.5 L, the design case is 452 mm and *W_d_* = 46.3 kN, and the numbers become: three 30 × 30 mm tubes at *SF_y_* = 2.13 and *SF_b_* = 3.41; three 25 × 25 mm tubes at 2.02 and 2.48; three 35 × 35 mm tubes at 2.49 and 4.29. The 25 × 25 mm section, therefore, clears the criterion on the wetted capacity by 1.0% and fails it on the envelope by 0.9%, and the recommendation of 30 × 30 mm rests on the envelope, which Section 2.3 adopts for the reason stated there. That is a judgement, and it is priced: a fill test returning a capacity nearer 1391 L than 1417 L would make the answer three 25 × 25 mm tubes at 100.27 kg on a margin of 1.0%—an eighth of the connection-factor band—so the lighter section is admissible in arithmetic and not in margin. The section that clears the band on either definition is 35 × 35 mm, 2.44 and 2.49, the fallback costed below.

The strength margin is narrower than the connection-factor band, and that is the one thing this analysis cannot close. The substitution factor *k_σ_* = 1.055 is measured within the eight-bar family and transferred to the three-tube configuration by the argument in Section 3.10(2); across bar diameters at fixed count the deformation of the same rack varies by 8.3% (Section 3.6), and taking that whole span as a half-width results in a ±8.3% band on the transferred factor. It is a sensitivity interval, not a prediction-error bound: it assumes that the error of extrapolating out of the family is no larger than the largest variation within it, and no independent evidence establishes that assumption. On *SF_y_* = 2.09, the band is [1.92, 2.27]; its lower end is below the criterion, and the margin of 4.6% is narrower than the band it has to cover. Three considerations make the same *relative* band defensible on the stress: the stress comes from the same measurements by the same substitution and no separate stress-error estimate exists; *k_σ_* is closer to unity than *k_δ_* = 1.090, so a proportional band is, if anything, conservative for it; and the band is applied to the *design* stress and not to a local peak. Two outcomes are consistent with step (i) in Section 4.5, and both are costed: a recovered factor within 4.6% of 1.055 leaves the 30 × 30 mm section at 101.34 kg, and a factor outside it replaces them with three 35 × 35 × 2.5 mm tubes at 102.40 kg, *SF_y_* = 2.44 and a band lower end of 2.24, at 1.06 kg or 1.0%. The uncertainty is, therefore, bounded at 1.0% of the rack mass. On the candidate, whose margin is 1.48, the same band spans [1.35, 1.60], below the criterion at both ends.

The load-scaling interval in Section 2.3 runs the other way and is independent of the band. At its favourable end, *SF_y_* = 2.26, the band becomes [2.07, 2.45] and clears the criterion at both ends, while 25 × 25 mm reaches 2.14 and its band, [1.96, 2.32], still does not. The section is, therefore, 30 × 30 mm on either reading of the scaling, and 25 × 25 mm only at the favourable point value of that reading, where its own band still crosses the criterion: the choice is not a computation that the 0.9% margin carries, nor a judgement that overrides the numbers; it is the lightest section that clears the criterion on the conservative reading of the two load-side judgements this paper offers—the capacity definition and the treatment of the dead load in the scaling—and not on the connection-factor band, which is a sensitivity interval whose value is still to be measured rather than an input whose uncertainty is known. That band is carried by step (i) in Section 4.5, and it is the reason the *margin*, and not the *section*, is described as provisional: the section is established, the margin it carries is not. The cost of that rule is 1.07 kg.

**(5) End of life.** The accidental case is checked on the nominal wall, for the reason Section 3.8(4) presents, an accidental action and a ten-year service allowance are not normally combined, and combining them here would demand a section that no standard tube supplies. What the allowance does, instead, is to lower the admissible level. With the 1.75 mm wall, the recommended scheme reaches *SF_y_* = 2.0 at 364 mm and *SF_b_* = 2.0 at 575 mm, so strength governs end of life, as it does when new, and the admissible level falls from 460 mm to 364 mm over the ten-year life—6.1 times the deepest operating point on the same flat-plan axis. A pan-full accident in year 10 would be carried at *SF_y_* = 1.58 and *SF_b_* = 2.44, so the end-of-life margin on strength does not reach the criterion; the requirement that follows is not a heavier section but the coating and inspection regime in Section 4.5(7), because a section heavy enough to carry the accident on the corroded wall would cost more mass than the ten-year saving it protects.

**(6) What would change these numbers.** Three levers and their order is not the one a load-based reading would provide. The connection factor is the largest, because it is the only one that can move the governing margin across the criterion: at ±8.3%, it spans 1.92–2.27 against a required 2.0. The effective length factor is second and, for this section, small: *P_cr_* of the 30 × 30 mm tube rises from 52.64 kN at *K* = 1.0 to 55.96 kN at 0.85 and 58.75 kN at 0.70, so *SF_b_* moves from 3.35 to 3.57 and 3.74, 11.6%, and no section changes—against 42% on the 20 × 20 mm section. The axial-force distribution is third: at 1.2 times, the mean column load, *SF_b_*, falls from 3.35 to 2.80, well above the criterion. None of the three changes the section except the first, which is why step (i) in Section 4.5, and not a refinement of the load model, stands between this analysis and a released design.

## 4. Discussion

### 4.1. Where the Mass Is and What That Implies for Lightweight Design

The decomposition in Section 3.3 locates the mass, and the ceiling it imposes is a property of the structural form rather than of the search: the three-ring mesh frame is three quarters of the rack mass, and no refinement of the tubes and bars can reduce the total by more than about a fifth. The general relevance of this result is that in a multi-member load-bearing system the benefit of lightweight design depends first of all on whether the optimisation addresses the members that carry the mass, and not on the sophistication of the search algorithm applied to the members that do not. An attribution of the mass before the parametric study begins costs almost nothing—the decomposition used here needs only a set of mass outputs that the finite element analysis produces anyway—and it prevents a large computational effort from being spent on a substructure whose influence on the objective is bounded in advance. It is recommended as a standard preliminary step in this class of design procedure.

There is a second practical reading of the same result. In the present rack, the frame is excluded from the design space *because* it is a welded assembly that cannot sensibly be re-sectioned, which is a fabrication argument and not a structural one. The decomposition turns that argument into a price: 74.9% of the steel is locked behind a manufacturing decision. The next step in reducing the mass of this family of equipment is, therefore, a change in the topology of the frame—a trussed ring instead of a solid welded ring or a mesh deck that is detailed to act as a plate in bending—rather than a further exploration of column sections. The same logic applies to the design of similar tiered rearing furniture, where the practical constraint on change is almost always the fabrication route rather than the analysis.

### 4.2. The Governing Limit State and the Role of the Operating Water Depth

The most transferable finding of this study concerns not the rack but the way the requirement is stated. The operating water depth is decided by the animals—the cocooning substrate must lie above the water line, which forces a shallow pan to be operated shallowly—and it is, therefore, not a free structural parameter. Once that is recognised, the structural requirement becomes a function of a single process variable that the producer controls, and the answer takes the form of a chart rather than of a single design: up to 315 mm of water, the lightest standard section is admissible at 99.21 kg, beyond that the answer steps up discretely in increments of about 1.1 kg—100.27 kg to 456 mm, 101.34 kg to 481 mm, and 102.40 kg to 562 mm—and above 562 mm, it moves to configurations with more tubes (Figure 13, Table 13). A design chart indexed on the husbandry variable is a more useful deliverable than a single optimum, because it tells the producer what each extra centimetre of water costs in steel, and because the depth will, in practice, be chosen for the animals and not for the structure.

Two secondary findings sharpen the same point. First, which criterion governs is a property of the scheme and not of the assumed load case, and the quantity that carries it is the ratio *SF_y_*/*SF_b_* = *σ_y_W_d_*/(*σ_d_* Σ*P_cr_*), evaluated at one load case: in the design case, it lies between 0.170 and 1.027 over the thirty-six schemes in Table A1—below unity for 34 of them and just above it for the two 20 × 20 mm schemes with the fewest tubes—so strength governs all but the slenderest corner of the design space. The ratio moves between depths, because the dead load does not scale with the water (Section 3.8(2)); it does not move between schemes, and a design study that reports a “governing criterion”, which changes with the assumed load case, is reporting an artefact of that case. Second, stability capacity is, nevertheless, the more fragile quantity: reducing the design from eight 25 × 25 mm tubes to three 20 × 20 mm tubes retains 22.1% of the buckling capacity but 37.6% of the strength margin, and the corrosion allowance removes a further quarter of the buckling capacity. The two statements are consistent—the smaller margin can belong to the less sensitive quantity—and both are needed, which is why they are reported together rather than as one “governing constraint”.

The methodological result with the widest application concerns the normalisation convention. The ranking of a discrete design set by TOPSIS is not invariant to the normalisation convention: under vector normalisation with a cost-type ideal, the best scheme is the one that a mass-dominated objective would select, whereas under range normalisation with a benefit-type ideal, the ranking moves substantially (Spearman *ρ* = 0.172, Scheme 5 from first to eleventh, only five schemes common to the two top-ten lists). Both conventions are standard, and each is defensible in isolation, so the choice between them has to be made explicitly and reported. Beyond that specific result, the sensitivity study, as a whole, shows that a ranking can be robust to the preference judgement (4913 enumerated judgement matrices) and to the data (36 leave-one-out re-estimations) and still be fragile to the criterion set and the alternative set. The safe procedure is to state the objective, apply the mandatory constraints first, and let the ranking corroborate the choice rather than make it—which is what Section 3.4, Section 3.5 and Section 3.7 do here.

### 4.3. Comparison with Existing Studies and with Established Practice

Published lightweight studies. Tian et al. [16] optimised a 40 m deep-sea floating-raft cage with a genetic algorithm and reported a 17.98% mass reduction at constant strength; Zhang et al. [17] showed that a spatial grid cage distributes stress uniformly with low steel consumption; Wang et al. [18] used response surfaces to trade displacement against volume in a rotatable cage; and the platform studies of Li et al. [19] and Shen et al. [20] established that the supporting structure and not the net can govern the design. The present case achieves 18.4% within the tube-and-bar subsystem, the same order of magnitude, but by a different route: discrete combinatorial selection among standard sections rather than continuous optimisation of one subsystem and the finding that even a complete enumeration of that subsystem is bounded by the mass of the frame it cannot touch. That is a general caution for the field, and it is consistent with Zhang et al. [17]: once section-level optimisation saturates, changing the form of the structure is the only remaining route.

Established engineering practice. The rack examined here is a cold-formed thin-wall welded steel structure protected by hot-dip galvanising—the ordinary construction of agricultural and storage racking—and the checks that decide it are the ordinary ones: the intermediate-column regime, the effective length of a welded compression member, the distribution of a gravity load between nominally identical columns, and a corrosion allowance taken from the exposure classification. The industrial literature reaches the same place from the opposite direction. Deng et al. [27] showed for storage racks that load *distribution* between uprights, rather than the upright section, drives capacity; Mohan et al. [31] showed for web-stiffened cold-formed built-up columns that local–global interaction can consume capacity well below the yield stress; Jakubovičová et al. [28] found that load-bearing performance has to be traded against cost efficiency; and the coating-durability literature [32] establishes that what survives a service life of condensation and immersion is not the nominal section. Each is an industrial confirmation of a check the earlier design of this rack had omitted.

The same argument has a quantitative form. The specific buckling capacity of the nine standard sections in Table 12, at the 890 mm column length used here, spans 16.4–28.7 kN·m/kg (*P_cr_L*/*m*): 16.4 kN·m/kg for 20 × 20 × 2.5 mm and 28.7 kN·m/kg for 60 × 60 × 2.5 mm, against the theoretical asymptote for a stocky Q235 column, *σ_y_*/*ρ* = 29.94 kN·m/kg. Standard thin-wall sections, therefore, retain between half and nearly all of the material-limited capacity in this application—54.7% for the 20 × 20 × 2.5 mm section and 95.7% for the stockiest—and the residual loss comes from the slenderness of the compression members, not from the grade of steel. The 20 × 20 × 2.5 mm section sits at *λ* = 123.3, which is 95.1% of the transition value, *C_c_*, and there the two column curves are within 0.9% of each other (128.63 MPa from the Johnson parabola against 129.80 MPa from the Euler formula), with d*σ_cr_*/d*σ_y_* = 1 − 2*σ_y_λ*^2^/(4π^2^*E*) = 0.095—so it is the only section in the list whose capacity still responds materially to the grade of the steel, and it is 5% from the point where even that response disappears. The yield strength at which that section crosses into the elastic regime is 2π^2^*E*/*λ*^2^ = 259.6 MPa, so raising the grade from Q235 to Q355 leaves its buckling capacity at 129.8 MPa: the substitution buys nothing. The design is, therefore, insensitive to material substitution and highly sensitive to the number and effective length of the columns, which is the conclusion the industrial racking literature reaches from the load-sharing side.

Two points define the scope of this comparison. First, medicinal-leech breeding racks have no published structural data: no commercial product documents its section schedule, its span and its load case together, so the comparison is made at the level of structural principle and standard section capacity, and closing that evidence gap is part of what this paper contributes. Second, the baseline is the author’s own earlier unpublished work on this rack, identified as such in Section 2.4 and not a third party’s published result; the values quoted are reproduced from its finite element solutions, listed in full in Table A1 and Table A3.

Methodological comparison. Jiang et al. [21] used entropy-weighted TOPSIS to select from a Pareto set generated by NSGA-II, and the same recipe recurs throughout mechanical lightweight design [22,23,24,25,26,29,30]. Two additions are made here. First, entropy weighting used alone can invert a cost-dominated objective when the dispersion structure of the criteria is unbalanced: in the round-bar subsystem in Section 3.6 the entropy weights make the deformation criterion dominant and the method recommends a scheme 5.6% heavier than the one it replaces, in exchange for a 5.2% stress reduction—the opposite of the stated objective. Second, the combination coefficient, α, is not a sufficient sensitivity analysis: sweeping it over its whole range leaves this selection unchanged, while deleting a single criterion or changing the normalisation convention moves the leading scheme, so a study that reports only the α sweep is reporting the perturbation that happens not to matter.

Finally, the response-surface part of the method defines a limit that applies to this class of study. The second-order surface reproduces the mass almost exactly (*R*^2^ = 0.999973), describes deformation usefully (*R*^2^ = 0.8797), and reaches only *R*^2^ = 0.7316 (*R^2^_pred_* = 0.5939) for the peak stress, because the peak stress is a local quantity set by a geometric discontinuity and by the load path into the columns. A surrogate model, therefore, has to be validated quantity by quantity, and a response it predicts poorly is one it must not be used to generate—the rule adopted in Section 2.7(3), and the reason every stress value quoted here comes from a finite element solution.

### 4.4. Husbandry Implications for the Design of Leech-Rearing Equipment

The procedure developed here was derived from the animals’ requirements, and three of its inputs are husbandry inputs in a strict sense: the operating depth is fixed by cocooning behaviour, the moist substrate layer above the water line is a load rather than a decoration, and the uniformity of tier geometry is a husbandry outcome rather than an aesthetic one. What that yields for the practice of leech farming, and the boundary within which the structural results apply, is stated here.

What the study establishes. (1) The cocooning substrate is a real structural load and not a bookkeeping entry: the 41.6 kg allowance adds 7.2% to the load carried on the water the cone holds at the deepest operating depth, 3.9% on the flat-plan axis in Table 3, and 0.9% at the accidental level, larger than the 2.1% mass difference that separates the recommended configuration from the lightest one, so a model that treats water as dead weight and the substrate as nothing omits a term of the same order as the decision it is making. (2) A cocooning station held above the water line fixes the operating depth, and the depth fixes the mass, so the chart in Section 3.9 lets a producer read the structural consequence of a husbandry decision directly; the lightest standard section remains adequate up to 315 mm on the load axis, more than five times the load at the deepest operating depth the species needs, which is why the accidental level rather than the operating one is what the chart is read against. (3) Stiffness, expressed as the deflection-to-depth ratio, stays within 1% of the water depth across the whole range: the most flexible scheme considered deflects 0.33 mm at the deepest operating depth and 2.55 mm in the design case, 0.55% of the depth in both cases, because the deflection in this scaling is carried by the water alone and the depth measures the same water. A deeper pan, therefore, does not degrade the relative uniformity of the water film, and a producer choosing among these schemes is choosing cost and reserve rather than the conditions experienced by the animals. (4) Because the rack is the largest single steel consumer of the facility, the commercial benefit of lightweighting accrues to the producer who applies it.

What the structural result delivers. The deliverable is the structural input to a husbandry comparison: a load case containing the husbandry variables the earlier design omitted, a structure specified against them, and a depth-indexed chart of admissible rack mass expressed in the variable in which the husbandry decision is actually made. Husbandry terms enter throughout as requirements the structure satisfies and as loads it carries, both represented in the load case: “uniform conditions across tiers” is the deflection-to-depth ratio in Section 3.8(3), and “a wetted cocooning substrate” is the 41.6 kg allowance of Equation (1). The animal response is the next study—whether a lighter, stiffer, uniformly loaded rack produces measurably better growth, survival or cocoon yield in *Hirudo nipponia* or *Whitmania pigra* requires a controlled comparison with the animals as the experimental unit—and this paper supplies the structure and the load case such a comparison needs.

The species and production model to which this applies. The load case is that of Chinese indoor multi-tier production of *Whitmania pigra* and *Hirudo nipponia*: blood-fed *H. nipponia* and snail-fed *W. pigra* (Section 1.3), a room held at 22–28 °C, pans operated at 20–60 mm of water with the cocooning substrate above the water line, and a stocking density set by the operator. The title names those species because the number the whole analysis turns on—the operating depth—is a husbandry decision and not a structural one, and it is not the same decision elsewhere.

Mediterranean and European production runs principally on *Hirudo verbana* and *H. medicinalis*, both listed on CITES Appendix II and both used clinically in reconstructive surgery [39,40]; the animals traded as *H. medicinalis* are frequently not that species at all [39]. Their husbandry differs in the three inputs that set this load case. Broodstock are held at 21–24 °C rather than 22–28 °C; cocoons are deposited in cocopeat held at about 40% humidity with the water column roughly 100 mm deep, as reported for aquarium culture [41], rather than in a 25 mm litter layer above 20–60 mm of water; and the medium itself must be screened for ionic content, because substrates reaching 9.47 and 10.14 dS·m^−1^ killed every broodstock animal within four days, while unamended peats at 1.19 and 3.69 dS·m^−1^ resulted in normal cocoon output [35], and the composition of the blood meal alone changes the reproductive performance [42]. None of those differences is a refinement of the present result. A 100 mm operating depth is 67% deeper than the deepest level this case specifies, and it changes the husbandry requirement rather than the section: because the design case is the rim of the vessel and not the operating depth, the European case has to be re-entered in Table 13 with its own pan geometry and its own substrate load—on which entry the answer is a different section from the Chinese one—and the chart returns that answer without being rederived. The chart is parameterised by load, so it can be re-entered for another species once its depth and its substrate are known; it cannot be extrapolated to one. The numeric conclusions are, therefore, stated for the two species and the one production model named in the title, and the transferable contribution is the procedure and the form of the chart rather than its entries.

Two further inputs are bounding values, each with a defined route to measurement. No stocking-density ceiling is fixed in this study: the ceiling is an operator input (Section 2.1), and the operator’s density figure, supplied with a mean body mass, enters Equation (1) as the added biomass term whose position Section 4.5(4) specifies, after which the rack is dimensioned against the load Equation (1) then returns. What the recirculating-systems literature cited in Section 1.3 supports is the *welfare* argument for keeping the density below a ceiling, not the load, and no ceiling value is quoted from it here. The environmental conditions used to classify the corrosion exposure—22–28 °C, above 80% relative humidity, splashing at each water exchange—are the rearing-room inputs listed in Table 2, and step (v) of the verification programme replaces them with a measured corrosion rate.

### 4.5. Verification Programme and Acceptance Criteria for Manufacture

The verification programme falls into three groups—the source data, the model and the service assumptions. Each is listed with the action that closes it and the acceptance criterion at which it closes; steps (i)–(vi) at the end of the section give the protocol itself.

**(1) The source data are a full factorial without replication, and the interaction is established independently of it.** The 53 solutions reproduce a full factorial exactly, which is what makes the design space completely enumerated rather than sampled. With no replicate there is no pure-error estimate, so the residual of the additive analysis of variance pools interaction, lack of fit and noise; the interaction is, therefore, established by the two assumption-light routes in Section 2.7(2)—the stratified simple-effect ratio, 9.0 between the *n* = 8 and *n* = 3 columns, and the nested comparison in which the interaction term is added to a second-order surface that already contains both curvature terms, which lowers the unexplained sum of squares from 29.08% to 12.03% of the total (*F*(1, 31) = 43.9, *p* < 10^−6^). A replicate at a subset of the design points, or a three-level central composite design, would additionally make the interaction testable in the variance-components sense.

**(2) The mesh check covers the deformation only, and the stress concentration has its own protocol.** One refinement step was performed, for the maximum total deformation, and it is **one step and not an independence study**: the refined mesh changed that quantity by 0.30572%, which bounds its discretisation error at that level but does not demonstrate convergence. The peak equivalent stress sits at a geometric discontinuity in the mesh deck, where the solution is more sensitive to element size, and step (ii) refines the mesh locally there with its acceptance criterion; until that is done, the peak stress serves as a comparative indicator across the 36 schemes solved on one mesh. The contact parameters (μ = 0.2, rough floor contact), the equal-sharing assumption and *K* = 1.0 are the three model assumptions bounded here and tested in steps (i)–(iv): *K* alone accounts for up to 42% of the buckling capacity, and 1.2-fold unequal sharing costs 17% of the stability safety factor.

**(3) The round bars act as connection and restraint, and the solid-contact model is the test that resolves them.** Section 3.10(2) establishes this from the solved family: the substitution factor for deformation is 1.090 there, while an independently coded beam model of the same rack returns 1.003, so a beam idealisation cannot represent what the bars do. Neither configuration is independent of that effect: the 5 mm bar set is absent from the 53 solved models in both, it enters strength and stiffness in both through the same pair of substitution coefficients, *k_σ_* and *k_δ_*, and its mass enters both through Equation (16). The protocol below, therefore, re-analyses **both** configurations, and its acceptance criterion bears on both.

The protocol that closes items (2) and (3), in the order in which it should be executed, is as follows.

(i) Solid-contact model. Re-analyse the recommended and the candidate configuration with three-dimensional solid elements, the pan–rack interface represented as a frictional contact with μ measured on the actual glass-fibre-reinforced plastic and steel surfaces, and the pan bottom represented by its true footprint rather than as a concentrated load. This step carries the governing acceptance criterion of the study. *k_σ_* and *k_δ_* are to be recovered on the recommended configuration itself, at the tube number actually recommended and not only in the eight-tube family in which they were obtained, and the strength safety factor recomputed from the recovered *k_σ_* must remain at or above 2.0 in the design case on the 235 MPa basis. At the nominal factor, the margin is 4.6% and the ±8.3% band takes the lower end to 1.92, so this measurement, and no other step of the programme, decides whether three 30 × 30 mm tubes are admissible. The secondary criteria are that the deformation and the peak stress agree with the values used here within the ±8.3% band—itself a *deformation* spread whose transfer to the stress is the judgement declared in Section 3.10(4)—and that *k_δ_* agrees with the 1.090 of the bar family to within 15%, so that the 1.003 of the beam model stands as the failure of that idealisation and not as the lower end of an acceptance interval. The same re-solution returns the split of the peak stress between water and dead load, which is the other end of the interval in Section 2.3: at the mass share the design-case stress is 104.2 MPa, *SF_y_* = 2.26, and the band of the recommended section becomes [2.07, 2.45].

(ii) Stress convergence. Refine the mesh locally at the peak-stress location through at least three successive doublings of the local element density and report the change in the peak value; the acceptance criterion is that the peak stress used in the strength check changes by less than 5%.

(iii) Rotational restraint of the welded connection. Either measure the joint rotational stiffness on a representative welded assembly, or extract it from the solid model of (i) once it is validated, and re-run the stability check with the resulting *K*; the acceptance criterion is that the stability safety factor remains above 2.0 in the design case on the nominal wall and above 2.0 at the reduced level in Section 3.10(5) on the corroded one.

(iv) Static proof load test. Apply the design-case load to a prototype with the substrate allowance represented and compare the measured deflection with the computed value; the acceptance criterion is in agreement with the global deflection within 15%, the accuracy reached by the independently coded beam model in Section 3.10(2) on the calibration set.

(v) Service-environment exposure. Place galvanised coupons in the rearing room and measure the mass loss over 12 months to replace the C3 classification, which is currently inferred from the room conditions, with a measured corrosion rate; and measure the bulk density and the electrical conductivity of the medium actually intended, since the first sets the load of Equation (1) and the second decides whether the animals survive on it [35]. The acceptance criteria are a rate below 6.5 μm·a^−1^ and a density within 20% of the 600 kg·m^−3^ adopted in Table 2. For the medium itself, the target is an electrical conductivity at or below 3.69 dS·m^−1^, the highest value in [35] at which normal survival and cocoon output were recorded—but it is stated here as a provisional ceiling taken from one species on one substrate comparison, not as an acceptance criterion for the other two. The value that matters is species- and medium-specific, and the measurement is requested so that it can replace the borrowed figure rather than confirm it.

(vi) Load-basis re-solution. Re-run the 53 finite element models with the as-built pan geometry in the design case of Equation (1)—the rim capacity, the substrate allowance and the scheme’s own mass—so that the scaling in Section 2.3, one flat-bottomed pan of 1.86 m inner diameter at 300 mm, is replaced by a solution of the pan actually installed. This step should be executed first, even though it is listed last, because it removes the last extrapolation from the strength margins. The acceptance criterion is that the corrected strength safety factor of the recommended scheme remains at or above 2.0 in the design case. The capacity definition is closed at the same time and by measurement: fill one pan to its rim and determine the volume from the mass of water admitted or from a calibrated flow meter, and measure the laminate thickness at the rim, at mid-barrel and at the cone. The criterion is in agreement with the 1417.3 L envelope and the 4.6 mm wall to within 2%; a wetted capacity within 2% of 1391.5 L moves the design case to 452 mm and admits the 100.27 kg section, and the recommendation is then restated on that entry in Table 13.

**(4) The analysed load case is the gravity case, with the remaining actions enumerated as additions to *W*.** Gravity is represented in full: hydrostatic pressure at the pan-full design case, the dead weight of the pans and the substrate, and the rack self-weight. Wind, seismic action, maintenance and tray-handling live loads, erection and lifting, and the dynamic effect of rapid water-level change lie outside it, as do feeding loads—blood or gastropod prey, a residue that must be flushed promptly, and a mineral detritus that settles in the pan—which Section 2.2 identifies as both a load and a cleaning requirement. Quantifying them needs the feeding schedule, a husbandry input the producer holds and this study does not. Each action listed here is a separate addition to *W*, and the biomass term enters at the same place.

**(5) The design space is discrete and low-dimensional, and its ceiling is known.** Wall thickness was fixed at 2.5 mm, the frame held outside the design space, the two subsystems combined at the level of the final configuration, and the bar family spans four diameters and four counts at one tube configuration. The bound in Section 3.3—that this subsystem cannot change the total by more than about 25%, and that the lightest admissible section realises 18.4% of the baseline, nearly three quarters of that ceiling—makes the first two restrictions immaterial to the result and identifies the third as the one that matters. A continuous-variable formulation over the frame topology, with the deck represented as a stiffened plate, is the natural next study, and Section 4.1 states the two topology changes that would act on it.

**(6) Fatigue sits outside the analysed load spectrum.** Loading and unloading, cleaning and water-level cycling impose repeated action, and the welded joints are the likely location of fatigue damage. At the 47.8% strength utilisation reached in the design case, with the corrosion allowance carried as a reduction in the admissible level, fatigue is unlikely to govern under a load that is a single accidental excursion rather than a cycle; a fatigue check on the welded details, with the fracture assessment that would accompany it, is the natural extension of the proof-load test of step (iv).

**(7) The service assumptions are declared, costed, and made conditions of release.** The 10-year design life, corrosivity category C3, room conditions and the 41.6 kg substrate allowance are the assumptions listed in Table 2 with their basis. Each enters at a stated place: the first three through the corrosion allowance, the last through the load of Equation (1). The galvanising allowance is 6.93 kg on the baseline and 5.90 kg on the recommendation (Table A4); it is carried as a separate allowance and **not** inside the bare-steel masses, and including it changes the reduction from 18.4% to 18.2%, so omitting it would understate the delivered rack by 5.5% of its coated mass, 5.82% of the bare one.

Four conditions attach to the design life, and they are requirements on the delivered structure rather than assumptions about it: a coating of at least 65 μm on every member including the bars; annual inspection of the splash zone at the pan rim and of the weld toes; repair of any coating breach at the inspection that finds it; and re-coating before year 10 if the coupon rate of step (v) exceeds 6.5 μm·a^−1^. Two results carry the paper independently of the programme: the mass decomposition and its bounds—25.1% arithmetic, 20.1% buildable but inadmissible, 18.4% realised—follow from the finite element masses themselves, and the chart follows from Equation (1), the section properties and the standard section capacities. Both are reproducible from Table A1 and Table A3.

### 4.6. What This Paper Delivers and What It Does Not

The qualifications above are numerous enough to obscure the deliverable, so it is stated once, in one place.

**Delivered and usable as it stands.** (i) The mass decomposition of the baseline and the bounds that follow from it—a 25.1% arithmetic ceiling, 20.1% for the lightest buildable combination and 18.4% realised—none of which needs a load basis, because every term is a finite element mass. (ii) The ratio that decides which criterion governs, *SF_y_*/*SF_b_*, which is a property of the section at one load case and is quoted in the design case. (iii) The shape of the depth response—one step at each standard section and the depth at which each boundary is reached—because it is generated by the discreteness of the section list and by the load model of Equation (1), the second of which is a physical quantity and not a recovered one.

**Delivered and conditional.** (iv) The load basis of the finite element results: The free surface is recovered from the pressure contour and the scaling from the water-mass ratio (Section 2.3), so the margins are derived and not banded; what remains conditional is the as-built pan geometry and the bar set, steps (vi) and (i). (v) The recommendation itself—three 30 × 30 × 2.5 mm tubes with eight 5 mm bars at 101.34 kg—which is provisional in two stated senses and no others: its bar set is transferred by substitution rather than solved in place (Section 3.10(2)), and its strength margin of 4.6% is narrower than the ±8.3% band on that substitution, so the *section* is established by the checks above while the *margin* it carries is not; and the section 1.1% lighter clears the criterion only where the capacity definition and the load scaling are both read in its favour (Section 3.10(4)), which is the second reason the recommendation is stated as the minimum among those admissible on the conservative reading of each. Step (i) in Section 4.5 resolves both, and if it resolves the second unfavourably the fallback is one standard section up, at a cost of 1.0% of the rack mass (Section 3.10(4)).

**Not delivered and not claimed.** An experimental verification of any of the above; a fatigue assessment (Section 4.5(6)); a cost or fabrication study; and any statement about the animals themselves, no animal having been used or measured here (conclusion 9). A reader who needs one of these should not read this paper as supplying it.

## 5. Conclusions

A lightweight design procedure combining parametric finite element analysis (FEA), response surface and variance analysis, constraint-first multi-criteria decision making, and stability, serviceability and corrosion checks has been developed and applied to a three-tier annular industrialised breeding rack for *Whitmania pigra* and *Hirudo nipponia*. The load case was derived from the husbandry requirements of the animals rather than assumed, and the outcome is expressed as a function of the operating water depth. The conclusions are as follows.

**(1) The husbandry requirements of the species can be translated into a structural load case, and doing so changes the design.** Six of the nine requirements in Section 1.3 became explicit structural inputs (Table 2); feeding, the density ceiling and the handling loads are declared as not quantified (Section 4.5(4)). Two are decisive: the operating depth is fixed by the requirement that the substrate lie above the water line, and the substrate is itself a load—41.6 kg, 7.2% of the load carried on the water the cone holds at the deepest operating depth and 0.9% at the accidental level—absent from the earlier model.

**(2) The mass reduction achievable within the optimised subsystem is bounded by where the mass is 25.1% arithmetic ceiling, 20.1% buildable and inadmissible, and 18.4% realised.** The three-ring welded mesh frame is 92.97 kg, 74.9% of the 124.14 kg baseline; six square tubes of 24.41 kg and eight round bars of 6.77 kg. Equation (16) reproduces thirteen held-out schemes to within 0.047% and back-calculates a tube length of 905 mm against the 890 mm design value. The 9.98% of the earlier work is just over half of the 18.4% now delivered, and further progress requires changing the topology of the frame rather than the size of the columns.

**(3) The objective must be stated, the constraints must precede the ranking, and the ranking must corroborate with its own uncertainty.** Adding mass, deformation and stress with nominal weights of 0.5/0.25/0.25 makes the actual shares 67.8%, 17.7% and 14.5%; the correct order is to apply *SF_y_* ≥ 2, *SF_b_* ≥ 2 and *δ* ≤ *L*/200 first and then minimise the mass. So constrained, the recommendation is three 30 × 30 × 2.5 mm tubes with eight 5 mm bars, 101.34 kg, 18.4% below the baseline, at *SF_y_* = 2.09, *SF_b_* = 3.35 and 16.4% of the deflection allowance. The absolute minimum, 99.21 kg on three 20 × 20 mm tubes, fails both criteria (1.48 and 1.44) and is carried as a predicted candidate. The 1.1% to the 100.27 kg section is a choice, not a margin: that section clears the criterion only where the pan capacity and the load scaling are both read in its favour (Section 3.10(4)).

**(4) The sensitivity of a ranking must be established from several directions, and the normalisation convention decides it.** Over 4913 enumerated AHP matrices, 1087 of them consistent, and 36 leave-one-out entropy re-estimations, the reference scheme ranks first or second everywhere and first in 34 of the 36 runs; as a pure AHP weight, it falls as low as rank 18. Deleting the stress criterion moves it to fifteenth, and range normalisation moves it from first to eleventh, with a Spearman correlation of *ρ* = 0.172. The ranking is not convention-invariant, and the α sweep alone, which changes nothing, is not a sufficient sensitivity analysis.

**(5) Strength governs the design space, except at its slenderest corner, and stability capacity is the quantity most sensitive to reduction and to corrosion.** The ratio *SF_y_*/*SF_b_* is an identity at a fixed load case: in the design case, it lies between 0.170 and 1.027 over the 36 schemes, below one for 34 of them and just above it for the two 20 × 20 mm schemes with the fewest tubes. It is not invariant across depths, because the dead load does not scale with the water (Section 3.8(2)), so the governing criterion is quoted in the design case. All nine sections are intermediate columns, their slenderness of 37.9–123.3 below *C_c_* = 129.61, the Euler formula overstating the 25 × 25 mm capacity by 25.1%; the corrosion allowance removes 27.3% of the 30 × 30 mm buckling capacity, and because the lightest section is the stability-controlled one, it decides which section is admissible at all.

**(6) The operating depth does not select the design; the credible accidental level does.** Over 20–60 mm, no section is near a limit—at 60 mm it stands at *SF_y_* = 16.1 and *SF_b_* = 14.6 on the flat-plan axis, and at 18.5 and 26 on the water the cone holds—and what selects it is the pan standing full to its rim, 3 × 1417.3 L, 460 mm, 47.1 kN. The chart is read as the level at which each scheme is broken: 315 mm for the lightest section by stability, 456 mm for 25 × 25 mm and 481 mm for the recommended 30 × 30 mm by strength, 562 mm for 35 × 35 mm. The design case sits 21 mm inside that limit and within 4 mm of the lower edge of its own band, which is the whole of the mass the accident costs (2.13 kg, 2.1%) and the reason four millimetres of water separate 101.34 kg from 100.27 kg.

**(7) The chart and the verification protocol are the two deliverables that carry this work into manufacture.** The recommended 101.34 kg configuration has its tubes solved and its bar set carried by substitution, so it is a **provisional recommendation** and not a verified design: *SF_y_* = 2.09, *SF_b_* = 3.35, 16.4% of the deflection allowance. Its strength margin of 4.6% is narrower than the ±8.3% band on that substitution, so the lower end of the band lies at 1.92, and the margin is the one quantity the analysis cannot close; step (i) in Section 4.5 closes it and returns the load-scaling interval in Section 2.3 with it, whose favourable end would put the same band at [2.07, 2.45]. The fallback—one section up, 1.0% more mass—is costed in Section 3.10(4).

**(8) The load basis is derived from the records rather than reconstructed.** The pressure contour yields 0.00294 MPa, a 300 mm water column, so the free surface and the modelled pan are both known; the results are scaled by the water ratio 1417.3/815.2 = 1.739, which is exact for the hydrostatic part and yields an upper bound on the design-case stress—112.3 MPa and *SF_y_* = 2.09, against 104.2 MPa and 2.26 with the dead load carried at its own mass. **Every** strength margin here is the lower end of an interval 7.8% wide (112.3/104.2 = 1.078). The as-built pan geometry and the bar set remain outstanding, as steps (vi) and (i) of the protocol.

**(9) The scope is two species and one production model, and the title says so.** Mediterranean and European production of *Hirudo verbana* and *H. medicinalis* differs in all three inputs that set the load: broodstock at 21–24 °C, a cocopeat bed at about 40% humidity under a 100 mm column [41], and a medium screened for ionic content, as well as density [35]. A 100 mm column alone would not move the design case, which is the capacity of the vessel and not the depth the animals are kept at; the European model is a different vessel, and its capacity is a different entry in Table 13, which is why the chart is re-entered rather than read across. No animal was used or measured here.

## Figures and Tables

**Table 1:** Material properties.

Material	Density/(kg·mm^−3^)	Young’s Modulus/MPa	Poisson’s Ratio	Bulk Modulus/MPa	Shear Modulus/MPa
Structural steel (rack)	7.85 × 10^−6^	2 × 10^5^	0.30	1.6667 × 10^5^	76,923
Concrete (floor)	2.3 × 10^−6^	3 × 10^4^	0.18	15,625	12,712
GFRP (water tank)	2.6 × 10^−6^	7300	0.22	4345	2992

**Table 4:** Finite element model and solver settings.

Item	Setting
Geometry source	Parametric 3D model (SolidWorks)
Analysis type	Static structural (ANSYS Workbench)
Element type	Tetrahedral
Mesh size/mm	Tank 50; rack 20; concrete floor 100
Nodes/elements	184,693/94,468
Mesh refinement check (one step), maximum total deformation	0.30572% (one refinement step)
Mesh refinement check, maximum equivalent stress	Not established; see text
Contact 1 (tank–rack)	Frictional, μ = 0.2
Contact 2 (rack–floor)	Rough
Boundary condition	Concrete floor fixed
Load	Hydrostatic pressure to the rim of the modelled pan, 300 mm; free surface read from the contour legend (Section 2.3)
Outputs	Maximum total deformation, *δ*; maximum von Mises stress, *σ*

**Table 5:** Two-factor analysis of variance results.

Response	Source	SS	df	MS	*F*	*p*	Contribution/%
Mass, *m*	*S* (edge length)	834.72	8	104.34	22.42	2.36 × 10^−9^	34.16
	*n* (tube number)	1496.91	3	498.97	107.20	4.91 × 10^−14^	61.26
	Residual	111.72	24	4.65	—	—	4.57
	Total	2443.35	35	—	—	—	100
Max. deformation, *δ*	*S* (edge length)	5173.48	8	646.68	5.08	8.91 × 10^−4^	43.93
	*n* (tube number)	3549.11	3	1183.04	9.29	2.93 × 10^−4^	30.13
	Residual	3055.38	24	127.31	—	—	25.94
	Total	11,777.96	35	—	—	—	100
Max. stress, *σ*	*S* (edge length)	1071.98	8	134.00	5.41	5.88 × 10^−4^	16.52
	*n* (tube number)	4822.95	3	1607.65	64.86	1.17 × 10^−11^	74.32
	Residual	594.86	24	24.79	—	—	9.17
	Total	6489.78	35	—	—	—	100

Note: *F* and *p* refer to the additive model of Equation (10) only. Because the experiment is a single-replicate full factorial, there is no pure-error estimate, so the residual mean square mixes interaction, lack of fit and noise; the two *F* tests establish the main effects against that mixture and say nothing about the residual itself.

**Table 6:** Second-order response surface regression coefficients.

Response	*β*_0_	*β*_1_ (*a*)	*β*_2_ (*b*)	*β*_11_ (*a*^2^)	*β*_22_ (*b*^2^)	*β*_12_ (*ab*)	*R* ^2^	*R* ^2^ *_pred_*
*m*/kg	117.380	7.459	8.399	−0.115	−0.021	3.553	0.99997	0.99996
*δ*/10^−2^ mm	49.142	−15.378	−13.778	16.546	8.962	15.061	0.8797	0.7945
*σ*/MPa	43.928	−0.899	−15.849	3.757	7.733	1.305	0.7316	0.5939

Note: Coded variables *a* = (*S* − 40)/20 and *b* = (*n* − 5.25)/2.5. *R*^2^*_pred_* is the leave-one-out cross-validated value. The variance contributions quoted in this paper are marginal (Type III-style) sums of squares, with each term tested with all other terms present. For deformation, the marginal and sequential decompositions coincide; for stress, the higher-order terms are not significant under a marginal decomposition, and the stress surface is used only through its main effect of the tube number, which carries 65.97% of the total sum of squares of this second-order fit under a sequential decomposition (the 74.32% in Table 5 is the same effect’s share in the additive two-factor model).

**Table 7:** Hold-out validation of Equation (16) on the thirteen round-bar schemes that took no part in its calibration.

Scheme (Table A3)	*d*/mm	*k*	Model/kg	Tabulated/kg	Deviation/%
2	5	6	111.627	111.640	−0.012
3	5	4	111.521	111.530	−0.008
4	5	2	111.415	111.430	−0.014
6	10	6	112.579	112.600	−0.019
7	10	4	112.156	112.180	−0.022
8	10	2	111.732	111.750	−0.016
10	15	6	114.167	114.220	−0.047
11	15	4	113.214	113.250	−0.032
12	15	2	112.262	112.290	−0.025
14	20	6	116.389	116.360	+0.025
15	20	4	114.696	114.680	+0.014
16	20	2	113.002	113.000	+0.002
17	—	0	111.309	111.320	−0.010

Note: maximum absolute deviation of 0.047%; median of 0.016%.

**Table 8:** Weights, leading schemes and the position of the reference scheme under five decision rules.

Decision Rule	Weights (*m*, *δ*, *σ*)	Leading Eight Schemes	Rank of Scheme 5
AHP-weighted TOPSIS (α = 0)	0.5396, 0.1634, 0.2970	5, 9, 1, 6, 14, 10, 13, 15	1
Entropy-weighted TOPSIS (α = 1)	0.4433, 0.2004, 0.3563	5, 9, 13, 1, 6, 14, 10, 29	1
Combined weighting, α = 0.5	0.4915, 0.1819, 0.3266	5, 9, 1, 6, 13, 14, 10, 15	1
Gray relational analysis (combined weights)	0.4915, 0.1819, 0.3266	9, 5, 11, 15, 6, 16, 19, 1	2
Range normalisation, convention R	0.4915, 0.1819, 0.3266	11, 15, 6, 16, 7, 2, 19, 1	11

Note: Weights are given as (*w_m_*, *w_δ_*, and *w_σ_*). The complete relative-closeness values and the ranks of all 36 schemes under both normalisation conventions are given in Table A2.

**Table 9:** Summary of the six sensitivity perturbations applied to the selection.

Source	Perturbation	Extent	Optimum Scheme	Rank of Scheme 5
Preference, combined weight	enumeration of the AHP judgement matrix, α = 0.5	4913 matrices; 1087 with CR ≤ 0.10	5 (643 cases), 9 (444 cases)	1 or 2 in all admissible cases
Preference, AHP-only weight	the same 1087 matrices, entropy weight removed	1087 admissible matrices	9 (548 cases), 5 (295), 11 (105), 15 (84), 10 (23), 6 (21), 19 (11)	1 in 295 cases, 2 in 571, 3–5 in 77, 6–18 in 144; worst rank 18
Data	leave-one-scheme-out entropy weights	36 runs	5 (34 runs), 9 (2 runs)	1 or 2
Criterion set	delete one criterion in turn	3 runs	9/5/15	2/1/15
Alternative set	delete 4 schemes, or one edge-length level	5 runs, *n* = 32	5 in 2 runs, 9 in 3 runs	1 or 2
Combination coefficient	α ∈ [0, 1], 101 steps	101 runs	5 in all runs	1
Normalisation convention	convention R against convention V	2 runs	11 against 5	11 against 1 (Spearman *ρ* = 0.172)

Note: under “delete one criterion” the optima are listed in the order in which the criteria were deleted (mass, deformation, and stress); the corresponding leading triples are {9, 5, 29} without mass, {5, 9, 1} without deformation and {15, 19, 6} without stress (Section 3.5(3)), the entry in the table being the first of each triple.

**Table 10:** Optimum scheme under different decision rules.

Decision Rule	Optimum	Square Tubes	Mass/kg	*δ*/10^−2^ mm	*σ*/MPa
AHP-weighted TOPSIS	Scheme 5	25 × 25, *n* = 8	118.04	50.42	32.52
Entropy-weighted TOPSIS	Scheme 5	25 × 25, *n* = 8	118.04	50.42	32.52
Combined-weighted TOPSIS (α = 0.5)	Scheme 5	25 × 25, *n* = 8	118.04	50.42	32.52
Gray relational analysis	Scheme 9	30 × 30, *n* = 8	120.93	50.31	29.05
Range normalisation, convention R	Scheme 11	30 × 30, *n* = 4	110.29	62.08	42.88
Minimum mass, constraints not applied	Scheme 4	20 × 20, *n* = 3	105.46	134.40	86.78
Recommended: minimum mass with every criterion satisfied in the design case	Scheme 12 with 5 mm bars	30 × 30, *n* = 3	101.34	84.57 †	61.24 †
Recommended on the 250 MPa reference of the earlier design; fails at 235 MPa	Scheme 8 with 5 mm bars	25 × 25, *n* = 3	100.27	106.06 †	64.63 †
Absolute minimum mass (predicted candidate)	Scheme 4 with 5 mm bars	20 × 20, *n* = 3	99.21	134.40 †	86.78 †
Baseline (earlier initial design)	Scheme 26	50 × 50, *n* = 6	124.14	51.30	44.67

Note: The deformation and stress of the two joint configurations are the solved finite element values of the corresponding tube scheme at the reference load, *W*_0_, that is, of the tube frame carrying its standard eight 20 mm round bars (†), whereas their mass follows from Equation (16) and is the mass with the eight 5 mm bars. On the 5 mm bar set that the recommendation actually carries, the substitution factor in Section 3.10(2) and the water-mass scaling in Section 2.3 yield 1.603 mm and 112.3 MPa for the recommended scheme and 2.547 mm and 159.2 MPa for the candidate; it is those values, not the † ones, that the checks in Section 3.10 use. The two stress entries for the recommended scheme, 61.24 and 112.3 MPa, differ by the bar factor *k_σ_* = 1.055 and the scaling 1417.3/815.2 = 1.739; they are the same solution read at two points, not two solutions of different objects. Both configurations are checked against every criterion in Section 3.10.

**Table 11:** Mass reduction potential within the tube-and-bar subsystem.

Configuration	Mass/kg	Reduction vs. Baseline/%	Reduction vs. Earlier Final Design/%	Basis
Baseline (50 × 50, *n* = 6; 20 mm bars × 8)	124.14	—	—	finite element
Earlier final design (25 × 25, *n* = 8; 5 mm bars × 8)	111.75	9.98	—	finite element
Lightest tube scheme (20 × 20, *n* = 3; standard 20 mm bar set × 8)	105.46	15.05	5.63	finite element
Recommended (30 × 30, *n* = 3; 5 mm bars × 8)	101.34	18.37	9.32	finite element + Equation (16)
Predicted candidate (20 × 20, *n* = 3; 5 mm bars × 8)	99.21	20.08	11.22	Equation (16) + measured connection factor
Lower bound, bars omitted	98.78	20.43	11.60	Equation (16)

Note: The two joint masses are the values of Equation (16); the equivalent figures obtained by substituting the bar sets directly into the tabulated masses are 101.33 kg and 99.11 kg, differences of −0.01% and −0.10%, respectively. The earlier final design is taken here as the 25 × 25 mm, *n* = 8 tube scheme, with its 5 mm bar set (111.75 kg); the 20 mm bar set of the same tube scheme (Scheme 13 in Table A3, 118.04 kg) is the configuration selected in the round-bar study of the earlier work and is **not** the mass against which the reductions in the last two columns are computed.

**Table 12:** Buckling checks for the square tubes (wall thickness = 2.5 mm, length *L* = 890 mm, and *K* = 1.0).

*S*/mm	*A*/mm^2^	*I*/mm^4^	*r*/mm	*λ*	Euler *σ_cr_*/MPa	Johnson *σ_cr_*/MPa	AISC *F_cr_*/MPa	*P_cr_*/kN
20	175.0	9114.6	7.22	123.32	129.80	128.63	110.14	22.51
25	225.0	19,218.8	9.24	96.30	212.85	170.14	148.04	38.28
30	275.0	34,947.9	11.27	78.95	316.68	191.40	172.26	52.64
35	325.0	57,552.1	13.31	66.88	441.30	203.71	188.05	66.21
40	375.0	88,281.2	15.34	58.01	586.58	211.46	198.72	79.30
45	425.0	128,385.4	17.38	51.21	752.70	216.66	206.21	92.08
50	475.0	179,114.6	19.42	45.83	939.79	220.31	211.65	104.65
55	525.0	241,718.8	21.46	41.48	1147.23	222.97	215.69	117.06
60	575.0	317,447.9	23.50	37.88	1375.66	224.96	218.78	129.35

Note: The elastic–inelastic transition slenderness is *C_c_* = √(2π^2^*E*/*σ_y_*) = 129.61 on the 235 MPa basis, so 20 × 20 mm is the only section within 10% of the transition.

**Table 14:** Limit-state checks of the recommended scheme (three 30 × 30 × 2.5 mm tubes, 101.34 kg) and the predicted candidate (three 20 × 20 × 2.5 mm tubes, 99.21 kg), at the accidental design case in Section 2.3, 1417.3 L per pan, and at the deepest operating depth read on the flat-plan axis. All entries on the nominal wall; the corroded rows use the 1.75 mm wall in Section 3.8(4).

Check	Recommended, 60 mm Flat-Plan	Recommended, Design Case	Candidate, 60 mm Flat-Plan	Candidate, Design Case	Criterion
Total load *W*/kN	10.80	47.08	10.74	47.01	—
Deformation *δ*/mm	0.209	1.603	0.332	2.547	≤9.80
Peak stress *σ*/MPa	14.6	112.3	20.8	159.2	—
*SF_y_* = *σ_y_*/*σ*	16.1	2.09	11.3	1.48	≥2.0
*SF_y_*, corroded wall	12.1	1.58	8.9	1.16	≥2.0
*SF_b_* = Σ*P_cr_*/*W*	14.6	3.35	6.29	1.44	≥2.0
*SF_b_*, corroded wall	10.6	2.44	4.86	1.11	≥2.0
Deflection *δ*/(*L*/200)	2.1%	16.4%	3.4%	26.0%	≤100%
Column force/kN	3.60	15.69	3.58	15.67	—
Column buckling *P*/*P_cr_*	0.07	0.30	0.16	0.70	—

Note: Σ*P_cr_* = 3 × 52.64 = 157.9 kN for the recommended scheme and 3 × 22.51 = 67.53 kN for the predicted candidate, the tubes of one tier carrying the whole-machine load in Section 2.8(2). The stress and the deflection both carry the water-mass scaling in Section 2.3 and the measured connection factors *k_σ_* = 1.055 and *k_δ_* = 1.090, so the two columns describe the same 5 mm bar set, and neither is quoted on the as-solved 20 mm set. The corroded rows use *t_eff_* = 1.75 mm, with the peak stress and the deflection raised by the ratio of section moduli (1.324 for the 30 × 30 mm section, 1.274 for 20 × 20 mm) and the critical load reduced by the ratio of second moments of area (72.7% and 77.2%). *SF_y_* is *σ_y_*/*σ* on *σ_y_* = 235 MPa. It is quoted at the upper end of the load-scaling interval in Section 2.3, in which the dead load is carried with the water; carrying it at its own mass yields 104.2 MPa and *SF_y_* = 2.26 for the recommended scheme and 147.6 MPa and 1.59 for the candidate. The design-case columns quote the upper end of the load-scaling interval and the 60 mm columns the lower end, because the extrapolation from the archived solution runs upward to the design case and downward to the operating depth: the design-case columns quote the higher of the two readings and the 60 mm columns the lower, the other end of each 60 mm column being provided in this footnote. The two 60 mm columns are computed on the flat-plan axis, which carries 7.5 times the water the conical bottom holds at that depth (Section 2.3), so they are the heavier of the two readings of the operating condition; on the water the cone actually carries, the load is 6.09 kN, and Section 3.9 quotes the stress there at both ends of the load-scaling interval, 1.9 and 12.7 MPa. They also scale the design case by the water ratio alone, and because the dead load does not shrink with the water, that extrapolation understates the stress there: on this axis, the operating stress lies between the 14.6 MPa tabulated and 23.2 MPa, that is, an *SF_y_* between 16.1 and 10.1 and *SF_b_* = 14.6 on either value. The pair is quoted to show that the operating depth is not a design condition, not to be compared across depths.

## Data Availability

The finite element data set analysed in this study (36 square-tube and 17 round-bar design points) is provided in full in Table A1 and Table A3, and the derived multi-criteria results—weights, relative closeness and ranks under both normalisation conventions—in Table A2. Every number quoted in this paper can be recomputed from those tables together with Equations (1), (2), (12) and (13), which is the form in which the data underlying the results are made available. The analysis-of-variance and response-surface scripts, and the input files of the 53 solved models, are available from the corresponding author upon reasonable request.

## Abbreviations

The following abbreviations are used in this manuscript:

AHP	Analytic hierarchy process
ANOVA	Analysis of variance
AISC	American Institute of Steel Construction
CI	Consistency index of the AHP judgement matrix
CR	Consistency ratio of the AHP judgement matrix
FEA	Finite element analysis
GFRP	Glass-fibre-reinforced plastic
GB	Chinese national standard (Guobiao)
TOPSIS	Technique for order preference by similarity to an ideal solution

## Appendix A. Finite Element Results for the 36 Square-Tube Design Schemes

**Table A1:** Design schemes and finite element results (*S*, edge length; *n*, tube number; *m*, mass; *δ*, maximum total deformation; and *σ*, maximum equivalent stress).

Scheme	*S*/mm	*n*	*m*/kg	*δ*/10^−2^ mm	*σ*/MPa	Scheme	*S*/mm	*n*	*m*/kg	*δ*/10^−2^ mm	*σ*/MPa
1	20	8	115.01	58.48	38.98	19	40	4	113.07	55.38	45.51
2	20	6	111.24	69.23	48.67	20	40	3	109.77	61.46	68.31
3	20	4	107.35	100.38	66.74	21	45	8	129.51	51.01	48.71
4	20	3	105.46	134.40	86.78	22	45	6	122.06	51.97	49.98
5	25	8	118.04	50.42	32.52	23	45	4	114.60	55.69	60.38
6	25	6	113.50	55.66	40.50	24	45	3	110.87	61.67	67.97
7	25	4	108.83	78.72	51.44	25	50	8	132.26	50.04	37.77
8	25	3	106.59	106.06	64.63	26 *	50	6	124.14	51.30	44.67
9	30	8	120.93	50.31	29.05	27	50	4	115.98	54.35	58.14
10	30	6	115.69	52.81	41.80	28	50	3	111.91	59.88	72.18
11	30	4	110.29	62.08	42.88	29	55	8	135.04	50.21	33.04
12	30	3	107.68	84.57	61.24	30	55	6	126.22	50.69	43.16
13	35	8	123.74	50.88	36.06	31	55	4	117.37	53.09	56.48
14	35	6	117.78	52.20	40.09	32	55	3	112.95	58.08	65.91
15	35	4	111.68	56.97	43.07	33	60	8	137.81	49.90	34.57
16	35	3	108.70	67.33	52.45	34	60	6	128.30	50.40	38.75
17	40	8	126.53	50.77	40.17	35	60	4	118.75	52.34	44.10
18	40	6	119.87	51.79	44.91	36	60	3	113.99	57.14	70.41

* Scheme 26 is the original (baseline) design.

**Table A2:** Relative closeness, *C_i_*, and rank of all 36 square-tube schemes under the two normalisation conventions in Section 2.6 (combined weights, α = 0.5).

Scheme	*S*/mm	*n*	Convention V: *C_i_*	Rank	Convention R: *C_i_*	Rank
1	20	8	0.8305	3	0.7495	8
2	20	6	0.7015	20	0.7644	6
3	20	4	0.4232	35	0.6666	15
4	20	3	0.2366	36	0.5679	27
5	25	8	0.8819	1	0.7081	11
6	25	6	0.8244	4	0.7769	3
7	25	4	0.6460	24	0.7685	5
8	25	3	0.4296	34	0.6774	13
9	30	8	0.8722	2	0.6584	16
10	30	6	0.8052	7	0.7270	9
11	30	4	0.7912	9	0.8222	1
12	30	3	0.5242	29	0.7112	10
13	35	8	0.8202	5	0.5881	26
14	35	6	0.8160	6	0.6904	12
15	35	4	0.7966	8	0.7998	2
16	35	3	0.6672	23	0.7752	4
17	40	8	0.7719	11	0.5263	30
18	40	6	0.7556	16	0.6298	22
19	40	4	0.7661	14	0.7609	7
20	40	3	0.5145	31	0.6667	14
21	45	8	0.6801	22	0.4460	34
22	45	6	0.6928	21	0.5660	28
23	45	4	0.5941	27	0.6500	19
24	45	3	0.5147	30	0.6560	17
25	50	8	0.7585	15	0.4547	33
26	50	6	0.7412	19	0.5499	29
27	50	4	0.6171	26	0.6395	21
28	50	3	0.4816	33	0.6213	24
29	55	8	0.7669	13	0.4420	35
30	55	6	0.7470	17	0.5202	31
31	55	4	0.6339	25	0.6239	23
32	55	3	0.5380	28	0.6427	20
33	60	8	0.7449	18	0.4133	36
34	60	6	0.7738	10	0.5040	32
35	60	4	0.7682	12	0.6551	18
36	60	3	0.4986	32	0.6041	25

Note: Convention V is vector normalisation with a cost-type positive ideal, and convention R is range normalisation with a benefit-type positive ideal. The leading scheme is Scheme 5 under convention V and Scheme 11 under convention R; the Spearman rank correlation between the two orderings is *ρ* = 0.172.

## Appendix B. Round-Bar Design Schemes and Results

**Table A3:** Round-bar design schemes (square tubes fixed at 25 × 25 × 2.5 mm, *n* = 8).

Scheme	*d*/mm	*k*	*m*/kg	*δ*/10^−2^ mm	*σ*/MPa
1	5	8	111.75	54.97	34.43
2	5	6	111.64	57.36	33.32
3	5	4	111.53	61.91	34.60
4	5	2	111.43	80.04	39.42
5	10	8	113.03	53.29	35.15
6	10	6	112.60	56.56	32.79
7	10	4	112.18	59.42	35.56
8	10	2	111.75	74.01	39.04
9	15	8	115.18	50.53	35.22
10	15	6	114.22	54.79	33.43
11	15	4	113.25	58.83	35.03
12	15	2	112.29	72.93	36.96
13	20	8	118.04	50.42	32.63
14	20	6	116.36	53.72	34.83
15	20	4	114.68	55.75	36.83
16	20	2	113.00	72.51	37.88
17	—	0	111.32	117.57	41.62

Note: Scheme 17 is the control configuration without round bars. Scheme 13 is the final configuration selected in the original study and is the same configuration as Scheme 5 in Table A1; the two tables are separate finite element tabulations of that configuration and agree exactly on mass (118.04 kg) and on maximum deformation (50.42 × 10^−2^ mm), while its peak equivalent stress is 32.63 MPa here and 32.52 MPa there—a difference of 0.11 MPa, or 0.34%. Section 3.6 quotes the value given in this table; the discrepancy is confined to this single entry and is not resolved here. The choice between the two values changes no conclusion, and the sensitivity is stated rather than left implicit: taking 32.52 MPa would yield *k_σ_* = 34.43/32.52 = 1.059 in place of 1.055, which moves the recommended scheme’s design stress from 112.3 to 112.8 MPa and its *SF_y_* in the design case from 2.09 to 2.08. Scheme 1 is the round-bar configuration re-selected in this work on a mass-optimal basis.

## Appendix C. Load Basis and Coating Mass Audit

Section 2.3 recovers the applied load from the pressure contour—a free surface at 300 mm on a pan modelled at 2.7172 m^2^, carrying 815.2 kg of water—and the design case is the measured rim capacity of the pan, 1417.3 L. The scaling between them, 1.739, is, therefore, derived rather than assumed. Two quantities are still the largest levers in the analysis and are quoted in Section 3.8(1): the effective length factor, *K*, whose reduction from 1.0 to 0.70 raises the *P_cr_* of the 25 × 25 mm section from 38.28 to 45.7 kN, and the axial-force distribution, 1.2 times the mean lowering *SF_b_* in the design case from 3.35 to 2.80. Both are sensitivities of the analysis, not of the load case, and both err on the safe side at the value adopted.

**Table A4:** Galvanising mass, member group by member group, with *t_Zn_* = 80 μm and *ρ_Zn_* = 7140 kg·m^−3^. The rule is the plate fraction in Section 2.8(4) for a plate or a tube wall coated on both faces and the bar fraction of that section for a solid bar coated on its cylindrical surface.

Member Group	Bare Mass/kg	Coating Rule	Zinc Mass/kg	Share of Group
**Baseline, 124.14 kg**				
Three-ring mesh frame	92.97	plate, *t* = 2.5 mm	5.41	5.82%
Six 50 × 50 × 2.5 mm tubes	24.41	plate, *t* = 2.5 mm	1.42	5.82%
Eight 20 mm round bars	6.77	bar, *d* = 20 mm	0.10	1.46%
Total	124.14	—	6.93	5.58%
Delivered mass	131.07	—	—	—
**Recommended scheme, 101.34 kg**				
Three-ring mesh frame	92.97	plate, *t* = 2.5 mm	5.41	5.82%
Three 30 × 30 × 2.5 mm tubes	7.94	plate, *t* = 2.5 mm	0.46	5.82%
Eight 5 mm round bars	0.42	bar, *d* = 5 mm	0.02	5.82%
Total	101.34	—	5.90	5.82%
Delivered mass	107.24	—	—	—

Note: The 5.82% figure is the plate value at *t* = 2.5 mm, and it coincides with the bar value at *d* = 5 mm, because a 5 mm solid bar has the same surface-to-volume ratio as a 2.5 mm plate coated on both faces (4/*d* = 2/*t*). Applying it uniformly is exact for the recommended scheme and overstates the baseline by 0.30 kg, or 0.24%, because the baseline carries 20 mm bars. On the coated basis, the reduction from the baseline is 18.2% instead of 18.4%.
